# The Hotdog fold: wrapping up a superfamily of thioesterases and dehydratases

**DOI:** 10.1186/1471-2105-5-109

**Published:** 2004-08-12

**Authors:** Shane C Dillon, Alex Bateman

**Affiliations:** 1 The Wellcome Trust Sanger Institute, Wellcome Trust Genome Campus, Hinxton, Cambridgeshire, CB10 1SA, UK

## Abstract

**Background:**

The Hotdog fold was initially identified in the structure of *Escherichia coli *FabA and subsequently in 4-hydroxybenzoyl-CoA thioesterase from *Pseudomonas *sp. strain CBS. Since that time structural determinations have shown a number of other apparently unrelated proteins also share the Hotdog fold.

**Results:**

Using sequence analysis we unify a large superfamily of HotDog domains. Membership includes numerous prokaryotic, archaeal and eukaryotic proteins involved in several related, but distinct, catalytic activities, from metabolic roles such as thioester hydrolysis in fatty acid metabolism, to degradation of phenylacetic acid and the environmental pollutant 4-chlorobenzoate. The superfamily also includes FapR, a non-catalytic bacterial homologue that is involved in transcriptional regulation of fatty acid biosynthesis.

We have defined 17 subfamilies, with some characterisation. Operon analysis has revealed numerous HotDog domain-containing proteins to be fusion proteins, where two genes, once separate but adjacent open-reading frames, have been fused into one open-reading frame to give a protein with two functional domains. Finally we have generated a Hidden Markov Model library from our analysis, which can be used as a tool for predicting the occurrence of HotDog domains in any protein sequence.

**Conclusions:**

The HotDog domain is both an ancient and ubiquitous motif, with members found in the three branches of life.

## Background

**Figure 1:**
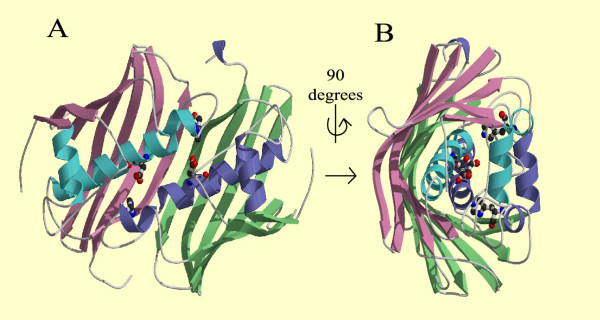
**The structure of the active HotDog domain dimer. **(A) A ribbon representation of the *Escherichia coli *FabA dimer (PDB code: 1MKB), viewed along the dyad axis. Each 171-residue subunit contains a Hotdog fold/ domain, consisting of a seven-stranded antiparallel b-sheet 'bun', coloured magenta and green, and a five-turn a-helical 'sausage' coloured blue and purple in the respective subunits. The Hotdog fold is best observed in Figure B. There are two independent active sites located between the dimers, the active site residues of His^70 ^from one subunit and Asp^84 ^from the other subunit, represented as a *ball-and-stick model *with CPK colouring (carbon, black; hydrogen, white; oxygen, red; nitrogen, blue), constitute the potential reactive protein groups in the active sites [1]. (B) A view of FabA rotated 90° along the dyad axis. The figures were generated with MOLSCRIPT [69] and rendered with RASTER3D [70].

We have found the HotDog domain, as we suggest calling the Hotdog fold, to be widespread in eukaryotes, bacteria, and archaea and to be involved in a range of cellular processes, from thioester hydrolysis, to phenylacetic acid degradation and transcriptional regulation of fatty acid biosynthesis. We present the superfamily and its functional subfamilies here. The Hotdog fold was first observed in the structure of *Escherichia coli *β-hydroxydecanoyl thiol ester dehydratase (FabA), where Leesong *et al*. noticed that each subunit of this dimeric enzyme contained a mixed α + β 'hot dog' fold [1]. They described the seven-stranded antiparallel β-sheet as the 'bun', which wraps around a five-turn α-helical 'sausage', see Figure 1. This characteristic fold has been found in a number of other enzymes, including: 4-hydroxybenzoyl-CoA thioesterase (4HBT) from *Pseudomonas *sp. strain CBS-3 [2] and *Arthrobacter *sp. strain SU [3], a novel gentisyl-CoA thioesterase from *Bacillus halodurans *[4] and in *Escherichia coli *thioesterase II [5].

## Results and Discussion

Although several proteins are now known to contain a Hotdog fold from structural analysis it has not to our knowledge been demonstrated that these proteins can be related to each other by sequence similarity. We have attempted to unify these structurally related proteins using a sequence analysis approach. Using sequence analysis means that we will identify additional proteins that are likely to contain a Hotdog fold. We have used the PSI-BLAST program [6] and used a representative of each Hotdog fold of known structure as a query against Swiss-Prot and TrEMBL protein database [7]. We used the sequences of the following PDB entries: 1C8U [5], 1IQ6 [8], 1LO7 [9], 1MKB [1], 1NJK [10], 1O0I [11] and 1PSU [12]. These searches have uncovered many novel members of this superfamily as well as finding links between the known structures with a Hotdog fold (see Table 1 and Additional file 1 ).

The Pfam database [13] contains a Thioesterase superfamily with 697 members, each member containing a 4HBT domain (accession: PF03061) corresponding to the HotDog domain. The SCOP database [14] contains a thioesterase/thiol ester dehydrase-isomerase superfamily, divided into 5 families, namely the 4HBT-like, beta-hydroxydecanoyl thiol ester dehydrase, Thioesterase II (TesB), MaoC dehydratase and PaaI/YdiI-like families.

**Figure 2:**
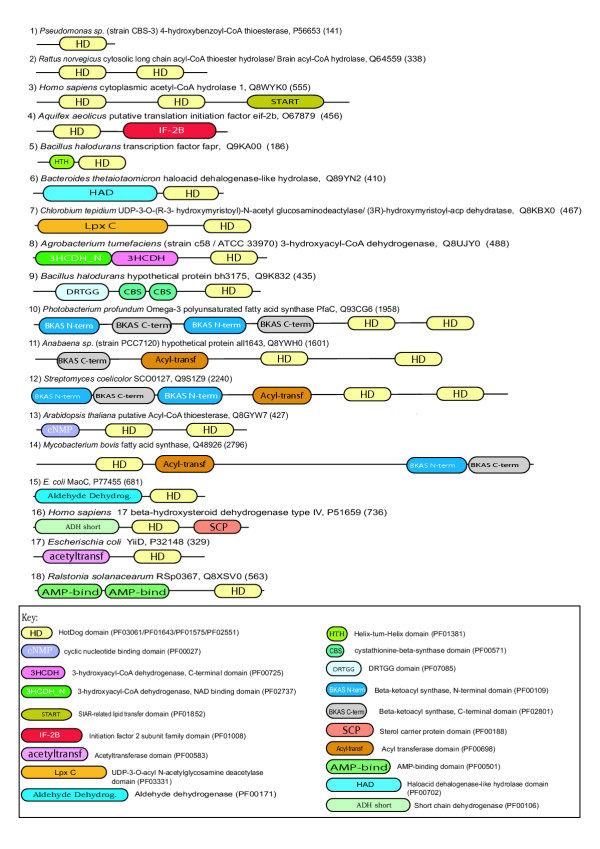
**A schematic Figure showing the various domain organizations of proteins with a HotDog domain**. For each distinct architecture we show an example protein, the species it has come from and its length is shown in parentheses. The key identifies all the domains in the Figure and also includes the Pfam accession number or numbers describing each domain.

Our searches have found a total of 1357 proteins (see Additional file 2 ) to be related to the known structures of HotDog domain proteins. We took these proteins and clustered them using single linkage clustering to define subfamilies with common functions. This clustering puts 1293 (95%) of the sequences into 85 clusters (see Additional file 3 ). The HotDog domain is found to be associated with a wide range of other domains. The various domain architectures are shown schematically in Figure 2. We describe the 17 subfamilies (Table 1) that have some experimental characterisation, below. The 17 subfamilies contain 909 proteins or 67 % of the total number of HotDog domain proteins. 384 (28 %) proteins cluster into the remaining groups, which contain predominantly hypothetical proteins or proteins that have no known function. They are not discussed here but we hope that our analysis may help in identifying functions for these proteins. Finally we have generated a Hidden Markov Model (HMM) library by concatenating together the HotDog domain sequences of the 85 clusters generated in our analysis (see Additional file 4 ). This library can be used in conjunction with the HMMER program [15] to search for HotDog domain(s) in any protein of interest.

### Acyl-CoA thioesterase subfamily

The largest subfamily represents over a hundred acyl-CoA thioesterases that are widespread throughout the prokaryotic kingdom, with members also found in eukaryotes. This group of enzymes catalyze the hydrolysis of acyl-CoA thioesters to free fatty acids and coenzyme A (CoA-SH.) [16]. The subfamily includes thioesterases with activity towards medium and long chain acyl-CoAs (medium chain acyl-CoA hydrolase and cytosolic long-chain acyl-CoA hydrolase/brain acyl-CoA hydrolase (BACH) respectively) and also cytoplasmic acetyl-CoA hydrolase (CACH), which hydrolyzes acetyl-CoA to acetate and CoA-SH. Brown-fat-inducible thioesterase (BFIT), a cold-induced protein found in brown adipose tissue (BAT) [17] is also included in this group. Both BFIT and CACH possess a StAR-related lipid-transfer (START) domain [18] that is involved in lipid binding, consistent with the role of BFIT and CACH in lipid metabolism. Duplication of the HotDog domain and recruitment of the START domain seems to be a mammalian innovation.

### FabZ like dehydratase subfamily

Members of this subfamily are found in a wide range of bacteria and sporadically in eukaryotes. In *E. coli *the products of the fab operon catalyze the four sequential reactions necessary for each round of fatty acid elongation [19]. The third step in each cycle of fatty acid elongation involves the dehydration of the β-hydroxyacyl-ACP protein intermediate by β-hydroxyacyl-[acyl carrier protein] dehydratase (FabZ) to give *trans*-2-decenoyl-ACP. FabZ is effective at dehydrating both short-chain and long chain saturated and unsaturated pathway intermediates.

This subfamily also contains a dehydratase component of the coronafacic acid (CFA) biosynthetic cluster encoded by the cfa2 gene [20,21]. CFA is the polyketide constituent of a phytotoxin called coronatine, which is a virulence factor of *Pseudomonas syringae*, a plant pathogen that causes disease in many agriculturally important plants [20].

### MaoC dehydratase-like subfamily

The *mao*C gene exists as an operon with the *maoA *gene in *E. coli *and is an enoyl-CoA hydratase involved in supplying (R)-3-hydroxyacyl-CoA from the fatty acid oxidation pathway to polyhydroxyalkanoate (PHA) biosynthetic pathways in *fadB *mutant *E. coli *strains. It was identified through its homology to *P. aeruginosa *(R)-specific enoyl-CoA hydratase (PhaJ1) [22]. PHAs are polyesters of (R)-hydroxyalkanoic acids, synthesized by numerous bacteria as an intracellular carbon and energy storage material in times of excess carbon sources [23], with intermediates of fatty acid metabolism such as enoyl-CoA, (S)-3-hydroxyacyl-CoA, and 3-ketoacyl-CoA acting as precursors for PHA biosynthesis [22]. The crystal structure of the (R)-specific enoyl-CoA hydratase (phaJ) from the *Aeromonas caviae *has shown that this enzyme also contains a Hotdog fold/domain [8]. The *E. coli *MaoC C-terminal HotDog domain is most likely responsible for its enoyl-CoA hydratase actvity. MaoC also contains an N-terminal short-chain dehydrogenase domain, involved in catalysing dehydrogenation of a variety of aliphatic and aromatic aldehydes using NADP as a cofactor. This subfamily also includes the human 17 β-hydroxysteroid dehydrogenase (17 β HSD) type 4, one of four different human 17 β HSDs that catalyze the redox reactions at position C17 of steroid molecules, one of the final steps in androgen and estrogen biosynthesis [24,25]. We also include a NodN-like sub-subfamily here that is found in another cluster containing several other MaoC proteins. *Rhizobium *and related species form nodules on the roots of their legume hosts, a symbiotic process that requires production of Nod factors, which are signal molecules involved in root hair deformation and meristematic cell division [26]. The nodulation gene products, including NodN, are involved in producing the Nod factors, however the role played by NodN is unclear.

### YbgC-like subfamily

This subfamily contains a large number of proteins about which very little is known except for the YbgC protein. The YbgC protein of the *tol-pal *cluster in the gamma-proteobacterium *Haemophilus influenzae *[27] has been shown to catalyze the hydrolysis of short-chain aliphatic acyl-CoA thioesters. The *tol-pal *cluster is present in many Gram-negative bacteria and is important for the maintenance of cell envelope integrity [28] and this operon is well conserved across gram-negative bacteria. Therefore we hypothesize that uncharacterized members of this subfamily are thioesterases.

The Asp^17 ^residue is conserved in YbgC from *Haemophilus influenzae *and *Pseudomonas aeruginosa*, along with the backbone amide NH of Tyr^24^, suggestive of a nucleophilic attack mechanism very similar to the *Pseudomonas *sp. strain CBS-3 thioesterase mechanism discussed below in the 4HBT class I section.

### FabA-like subfamily

The dehydration of the β-hydroxyacyl-ACP protein intermediate during the third step in each cycle of fatty acid elongation can be catalyzed by β-hydroxydecanoyl-ACP dehydratase/isomerase (FabA), as well as by FabZ, to give *trans*-2-decenoyl-ACP. FabA is uniquely able to isomerise trans-2-decenoyl-ACP to cis-3-decenoyl ACP, initiating unsaturated fatty acid biosynthesis [19] and is specific for acyl ACPs of 9–11 carbons in length.

Polyketides are a large and structurally diverse class of natural products, produced mainly by soil-dwelling bacteria such as *Pseudomonas *spp. and *Streptomyces *spp. They include clinically useful drugs such as the antibiotic erythromycin A and the immunosuppressants FK506 and rapamycin. The biosythesis of polyketides is very similar to that of fatty acids [21] and polyketide synthases (PKSs) have been classified as type I or type II according to fatty acid synthase (FAS) similarity. Most bacteria and plants use a highly conserved type II FAS system, which uses a distinct enzyme for each reaction. This is in contrast to the mammalian type I system (also used by fungi and some mycobacteria), which uses one multifunctional polypeptide to catalyze all reactions [29,30]. The HotDog domain is found in type II fatty acid synthesis in bacteria (FabA/FabZ), but also in a small number of bacterial polyketide synthases that are of the type I, being composed of several modules [31] such as β keto-acyl synthases and omega-3 polyunsaturated fatty acid synthase (PfaC). The marine bacteria *Shewanella *sp. SCRC-2738, *Moritella marina *strain MP-1 and *Photobacterium profundum *strain SS9 contain an eicosapentaenoic acid (EPA) biosynthetic cluster (*pfaA-D*), responsible for the synthesis of this omega-3 polunsaturated fatty acid (PUFA), [32,33]. The PfaC protein contains two HotDog domains (see Figure 2 for the domain organisation found in *P. profundum*), which are also found in the eukaryotic marine protist, *Schizochytrium*, suggesting that the PUFA synthetic cluster has undergone lateral gene transfer [32].

This subfamily also includes several fatty acid synthase proteins from bacteria, such as *Mycobacterium bovis *fatty acid synthase. This multifunctional protein is capable of catalysing de novo synthesis and chain elongation of fatty acids [34] and has a very similar domain architecture to the polyunsaturated fatty acid synthases, as it contains an acyl-transferase, β-keto acyl synthase N and C-terminal domains (see Figure 2).

The catalytic residues of FabA's bifunctional active site are His^70 ^and Asp^84^, His^70 ^is conserved in FabZ dehydratase, but Asp^84 ^is replaced with Glutamate. This replacement may be responsible for FabZ's inability to catalyze the isomerization reaction [1].

### Fat subfamily Acyl-ACP thioesterases

In plants, fatty acid synthesis occurs in the stroma of plastids, where the acyl chains are bound to the acyl carrier protein (ACP) during extension cycles [35]. Acyl-ACP thioesterases terminate fatty acid synthesis in plants by hydrolysing the thioester bond existing between an acyl moiety and the ACP [36]. In higher plants acyl-ACP thioesterases have been classified into two gene classes, *fatA *and *fatB*, based on sequence similarity and substrate specificities [37,38]. *Arabidopsis *FatA displays highest activity towards oleoyl-ACP whereas *Arabidopsis *FatB is most active towards palmitoyl-ACP [37]. This subfamily contains both FatA and FatB members [35]. The proteins in this subfamily range in length from 240 to 400 amino acids and therefore we hypothesized that they might contain two HotDog domains, located at the N and C teminal halves. By splitting the sequence of proteins from this subfamily into an N-terminal half and C-terminal half we were readily able to detect the relationship to other subfamilies using PSI-blast with query proteins such as Q899Q1 and Q42714, confirming our hypothesis.

### TesB-like subfamily

This subfamily contains the *E. coli *medium chain length acyl-CoA thioesterase II [5] encoded by the *tesB *gene [38], which is a close homolog of the human thioesterase II (hTE) enzyme. hTE catalyzes the hydrolysis of palmitoyl-CoA to CoA and palmitate and was identified as a human T cell protein that binds to the myristoylated HIV-1 Nef protein, correlating with Nef-mediated CD4 down regulation [39]. hTE could regulate targeting of the cytoplasmic Nef protein to the plasma membrane, which is dependent on a lipid modification, i.e. a myristoylation anchor and recombinant hTE shows maximal activity with myristoyl-CoA [39]. However further studies have shown that hTE localizes to peroxisomes [40,41], dependent on a C-terminal peroxisomal targeting sequence, SKL, and coexpression of Nef and hTE results in relocation of Nef to peroxisomes, so the role of Nef and hTE during HIV infection remains unsolved.

The catalytic site of *E. coli *thioesterase II was identified by site directed mutagenesis and involves a hydrogen-bonded triad of Asp^204^, Thr ^228^, and Gln ^278^, which synergistically activate a water molecule for nucleophilic attack of the carbonyl thioester carbon of medium chain length acyl-CoA substrates [5]. This is a novel reaction mechanism for a thioesterase and differs from the nucleophilic mechanisms used by β-hydroxydecanoyl dehydratase and 4HBT thioesterase in both *Pseudomonas *and *Arthrobacter *discussed below. This subfamily is found in bacteria and eukaryotes.

### 4HBT class II subfamily

This subfamily includes 4-hydroxybenzoyl CoA thioesterase (4HBT) from *Arthrobacter *sp. strains SU and TM1 encoded by the *fcbC *gene [3]. The *Pseudomonas *thioesterase uses the Asp^17 ^residue to mediate the hydrolysis reaction as discussed below in the 4HBT class I section. Gln^58 ^from *Arthrobacter *corresponds to the Asp^17 ^residue in *Pseudomonas *but inspection of the *Arthrobacter *strain SU active site has revealed the catalytic base (or nucleophile) to be Glu^73^, on the opposite side of the substrate binding pocket to Asp^17^[3]. Also the *Pseudomonas *thioesterase dimers form a tetramer with their long α-helices facing inwards, in contrast to *Arthrobacter *thioesterase where the dimers form a tetramer with their long α-helices facing outwards [3]. In *Pseudomonas *and *Arthrobacter *thioesterases, the 4-hydroxyphenacyl moieties are positioned in such an orientation that the thioester C = O interacts with the α-helical N-terminus by means of hydrogen bonding to a backbone amide NH, on Tyr^24 ^in *Pseudomonas *and Gly^65 ^in *Arthrobacter*, and it is this contact that results in polarization of the C = O for nucleophilic attack [3]. While the structure of *Arthrobacter *sp. strain SU thioesterase displays a similar Hotdog-fold topology to the 4HBT class I *Pseudomonas *enzyme, the enzymes differ at the level of catalytic platform, CoA binding site and quaternary structure [3,42]. This is not an unexpected finding as Todd *et al. *have found that 12 of the 31 superfamilies they analyzed displayed positional variation for residues playing equivalent catalytic roles [43].

A surprising inclusion in this subfamily is the ComA2 protein from *Bacillus subtilis*. ComA is a response regulator and transcription factor [44] that together with the histidine kinase, ComP, constitutes a two-component signal transduction system required for the development of competence. The *com*A locus is composed of two ORFs. ComA2 is cotranscribed with ComA1, which is required for competence while ComA2 is not [45], and so the role of the HotDog domain in this protein remains a mystery.

### PaaI subfamily

The phenylacetic acid (PA) catabolic pathway in *E. coli *has been characterised and found to contain 14 genes, allowing catabolism of this aromatic compound into likely Krebs cycle intermediates [46]. The *paa *operon in *E. coli *encodes PaaI, which is probably a thioesterase involved in the catabolism of PA. The catabolism of phenylacetic acid (PA) in *E. coli *begins with an activation step where Phenylacetyl-CoA ligase, PaaK, converts phenylacetate into Phenylacetyl-CoA. 4-chlorobenzoate-CoA ligase catalyzes a similar reaction at the first step of the 4-chlorobenzoate-degradation pathway. The thioesterase, PaaI, may be involved in a reaction similar to the last step in the degradation of 4-chlorobenzoate (see 4HBT class I below), however this remains to be demonstrated.

### FapR subfamily

This small subfamily is restricted to firmicutes. FapR is a highly conserved transcriptional regulator found in many gram-positive organisms, including all species of *Bacillus *[47]. It controls expression of genes involved in type II fatty acid and phospholipid biosynthesis, by binding to a consensus promoter sequence of the fap regulon and acting as a negative regulator. Malonyl-CoA, an intermediate in the lipid biosynthetic pathway, controls FapR. The HotDog domain has likely retained its substrate specificity for malonyl-CoA, but appears to have lost its catalytic ability, in common with the ligand binding domain of other transcriptional regulators. FapR contains a helix-turn-helix motif at the N-terminus (see Figure 2), which is similar to the DeoR transcriptional regulator family (data not shown), consistent with its role as a DNA binding protein.

### 4HBT class I subfamily

The crystal structure of 4HBT from the soil-inhabiting bacterium *Pseudomonas *sp. strain CBS-3 [2] has helped define the HotDog domain. A lot of attention has been focused on this microorganism because of its ability to survive on 4-chlorobenzoate (4CBA) as its only source of carbon [48]. 4CBA is a by-product of microbial degradation of industrial pollutants such as DDT and polychlorinated biphenyl herbicides [49] and this strain of *Pseudomonas *may be used as a bioremediation agent for degrading 4CBA. *Pseudomonas *sp. strain CBS-3 contains an *fcb *operon responsible for hydrolytic dechlorination of 4CBA, with 4CBA-CoA ligase (FcbA), 4CBA-CoA dehalogenase (FcbB), and 4HBT (FcbC) catalyzing sequential reactions that result in the degradation of 4CBA to 4-hydroxybenzoate. The thioesterase catalyzes the third step in the degradation pathway, which is the hydrolysis of the 4-hydroxybenzoyl-CoA thioester moiety to give 4-hydroxybenzoate and CoA [50].

4HBT from *Pseudomonas *sp. strain DJ-12 [51] is also found in this subfamily. The organization of the *fcb *operon in strain DJ-12 is different from that observed in strain CBS-3. The *fcb *genes are organised as B-A-C in both strains but strain DJ-12 has three ORFs between A and C called T1, T2, and T3 that are unique to this strain. These three genes are similar to the C4-dicarboxylate transport system in *Rhodobacter capsulatus*, suggesting that they may encode membrane proteins involved in the uptake of 4CBA [51]. This is in contrast to the gene organisation observed in the 4HBT class II, where *Arthrobacter *sp. strain SU and strain TM1 have an A-B-C order [51]. There is a duplication of the cluster in strain SU, where it is found on a plasmid, whereas only one copy exists in strain TM1, where it is located chromosomally. Both operons contain a T gene located at the end of the cluster, possibly involved in 4CBA uptake.

*Bacillus halodurans *C-125 contains a gene called BH1999, encoding a novel gentisyl-CoA thioesterase, which catalyzes the hydrolysis of gentisyl-CoA (2,5-dihydroxybenzoyl-CoA)[4,52] to yield gentisate (2,5 dihydroxybenzoate). BH1999 is found in a gentisate oxidation pathway gene cluster in *B. halodurans*. Gentisate has been implicated as an intermediate in the degradation of several industrial aromatic compounds [4].

Gentisyl-CoA thioesterase and 4HBT from *Pseudomonas *perform different physiological functions but remain in the same subfamily because they are highly related. The active site residues Asp^16 ^and Asp^31 ^of gentisyl-CoA thioesterase align with Asp^17 ^and Asp^32 ^of 4HBT. These are crucial residues that are proposed to function in nucleophilic catalysis and substrate binding respectively. Loss of Asp^17 ^in the *Pseudomonas *enzyme effectively halts catalysis, while loss of the corresponding Asp^16 ^residue to the *Bacillus halodurans *enzyme only reduces its catalytic rate by 230-fold, perhaps indicating that the hydrolysis reaction does not proceed through an Asp^16^-mediated nucleophilic attack mechanism previously proposed for Asp^17 ^[53,4]. Asp^17 ^in *Pseudomonas *strain CBS-3 has been suggested to participate in nucleophilic catalysis rather than general base catalysis based on the following observations. The Asp^17 ^carboxylate is located at a distance of 3.2 Å from the substrate C = O thioester bond, its aligned trajectory and the absence of a water molecule near the reaction centre are all suggestive of a role for Asp^17 ^as a catalytic nucleophile [9,53]. Asp^32 ^in Pseudomonas interacts with the benzoyl OH of 4-hydroxybenzoyl-CoA [9] and perhaps Asp^31 ^plays a similar role.

### Other subfamilies/ members

In the above sections we have described the 11 subfamilies that have some functional characterization. In this section we describe the other 6 subfamilies that have no functional characterization, except they are associated with other domains or have been structurally characterized.

The CBS associated subfamily contains the hypothetical protein BH3175 from *Bacillus halodurans*. The BH3175 protein contains two homologous copies of the CBS domain [54]. Scott *et al*. have recently shown that tandem pairs of CBS domains act as sensors of cellular energy status by binding AMP, ATP, or S-adenosyl methionine and mutations in CBS domains impair this binding in several hereditary disorders [55]. Although we do not know the substrate or activity of this subfamily of the HotDog superfamily, we can suggest that this step is regulated in an energy dependent manner by the CBS domains.

3-hydroxyacyl-CoA dehydrogenase is an enzyme involved in fatty acid metabolism, catalyzing the reduction of 3-hydroxyacyl-CoA to 3-oxoacyl-CoA [56]. The hydroxyacyl-CoA dehydrogenase-associated subfamily includes 3-hydroxyacyl-CoA dehydrogenase from *Agrobacterium tumefaciens *strain C58, which contains a HotDog domain at its C-terminus and the two domains (3HCDH_N and 3HCDH) associated with 3-hydroxyacyl-CoA dehydrogenase activity are located at the N-terminus and central portion of this protein. The combination of activities may allow substrate to be passed from one domain to the next.

Other subfamilies in the superfamily include the YiiD protein from *E. coli*, where an acetyltransferase domain is fused. The human mesenchymal stem cell protein DSCD75 and its counterpart in mouse also contain a HotDog domain. A Structural proteomics project has shown that the conserved hypothetical *E. coli *protein YbaW contains a Hotdog fold [10]. Finally the *Ralstonia solanacearum *hypothetical protein RSp0367, containing a HotDog domain and two AMP-binding domains, found in proteins involved in ATP-dependent covalent binding of AMP to their substrate, is a member of another subfamily.

### Domain fusion events

**Figure 3:**
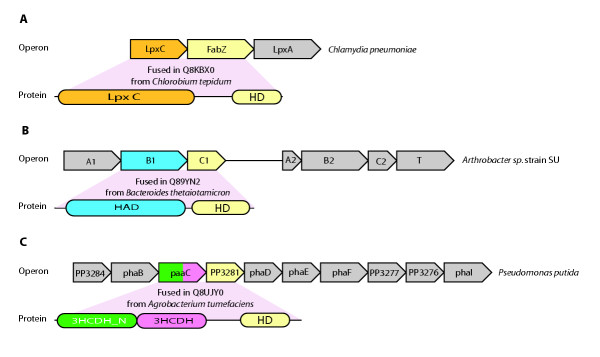
**Rosetta fusion proteins in the HotDog domain superfamily. **For each fusion event we show an example operon containing the two proteins separate and an example of the fused rosetta protein.

It has been shown that proteins that are functionally linked are occasionally found to be fused in various genomes. These fusion proteins have been termed Rosetta proteins [57,58] and can be used to predict the functional linkages of proteins with each other. The HotDog domain superfamily contains several rosetta proteins where the fused proteins are also found unfused in other genomes. In these cases they are adjacent to each other in known operons. The examples found in the HotDog superfamily are shown in Figure 3 and are described briefly here.

Within the FabZ subfamily the LpxC deacetylase domain (UDP-3-O-acyl N-acetylglucosamine deacetylase) is fused to the FabZ-like HotDog domain in *Chlorobium tepidium *(see Figure 3a). LpxC catalyzes the N-deacetylation of UDP-3-O-acyl N-acetylglucosamine deacetylase, the second and committed step in the biosynthesis of lipid A, which anchors lipopolysaccharide (LPS) in the outer membranes of most gram-negative bacteria [59]. The unfused proteins are found adjacent in operons from several species of chlamydia and cyanobacteria.

In the 4HBT class II subfamily we observed the order of the operon is ligase(A)-dehalogenase(B)-thioesterase(C). In *Bacteroides thetaiotaomicron *there is a Rosetta protein that contans a haloacid dehalogenase-like hydrolase domain (see Figure 3b). This domain architecture is similar to the *fcb *operon structure in *Arthrobacter*, with a dehalogenase-like hydrolase (HAD) domain and a HotDog domain (see Figure 3) i.e. it represents a fusion of the *fcbB *and *fcbC *gene products to form a novel protein in *B. thetaiotaomicron*.

The final domain fusion is in the 3-hydroxyacyl-CoA dehydrogenase from *Agrobacterium tumefaciens *strain C58, which possesses the HotDog domain, 3HCDH_N domain (3-hydroxyacyl-CoA dehydrogenase, NAD binding) and 3HCDH (3-hydroxyacyl-CoA dehydrogenase, C-terminal domain) domain (see Figure 3c). This may represent a fusion of the PaaC and PP3281 proteins in the gamma-proteobacterium *Pseudomonas putida *2440 phenylacetic acid degradation operon.

These fusion events suggest that the domain fusion process can occur in a simple scheme with two distinct phases. Firstly, two proteins are recombined into adjacent positions in an operon. Secondly, the two genes are then fused by a process of mutation that removes the stop codon at the end of the first gene and maintains reading frame through the second gene [60,61].

### Sequence motifs

The MASIA program [62] was used to search for HotDog domain motifs in the aligned sequences of the 17 subfamilies. The various motifs are found in Additional file 5 . It must also be noted that the PROSITE database release 18.29 [63] contains a consensus sequence motif (PS01328), called the 4-hydroxybenzoyl-CoA thioesterase family active site, and this is found in 29 Swiss-Prot, TrEMBL and TrEMBL-NEW entries cross-referenced with PS01328. This consensus pattern, [QR]-[IV]-x(4)-[TC]-**D**-x(2)-G [IV]-V-x-[HF]-x(2)-[FY], where D is the active site residue, is found in the YbgC-like subfamily and in the 4HBT-I subfamily. 19 of the 29 members are found in the YbgC-like group and 3 in the smaller 4HBT-I group. The remaining 7 proteins are scattered in various clusters consisting of hypothetical or unknown proteins. We have found, using MASIA, that this motif is found in the entire YbgC and 4HBT-I subfamilies, extending the number of proteins containing this motif to 107. We have also identified a HGG motif in the 4HBT-II and PaaI subfamilies. This motif is HGGAS-x-ALA**E **in the 4HBT-II subfamily and HGG-x-IF-x-LA**D **in PaaI members. The active site residue, Glu^73^, is known for 4-hydroxybenzoyl-CoA thioesterase from *Arthrobacter *sp. Strain SU, however the active site for *E. coli *PaaI is not known and we suggest that it is Asp^61 ^in the HGG motif above, which is 100 % conserved in all members of this subfamily (see Additional file 6 ).

## Conclusions

We have defined and analyzed the HotDog domain superfamily and in our analysis of this superfamily we have found 18 different domain architectures and defined 17 subfamiles. We have also investigated the domain organisation and the role that this plays in generating functionally diverse enzymatic and nonenzymatic functions based on the HotDog fold. Domain duplication, domain recruitment and incremental mutation have been key to the evolution of this superfamily. We have also looked at gene context and operon structures and found many examples of fusion proteins, in which the HotDog domain has been fused to another protein to generate functional diversity. The large number of subfamilies we have found, the diverse range of activities these proteins participate in and the taxonomic distribution of the HotDog domain indicates an ancient superfamily that has diverged substantially to fulfil numerous roles in the cell.

Our analysis may help with further experimental investigation of members of this superfamily. Some members of this superfamily, such as the *P. falciparium *FabZ enzyme have been proposed as a target for new anti-malarial drugs [64] as FabZ homologues are not found in humans. Finally our analysis identified hundreds of novel proteins such as human mesenchymal stem cell protein DSCD75 and the *Ralstonia solanacearum *hypothetical protein RSp0367 as probable enzymes potentially involved in lipid metabolism. Given that the large majority of proteins in this family are involved in bacterial lipid metabolism we suggest that the HotDog domain evolved in bacteria first and may then have been transferred to eukaryotes and archaea on several occasions. Since this time duplication and mutation has allowed it to fill a variety of roles.

## Methods

### Sequence analysis

All PSI-BLAST searches were carried out using default inclusion thresholds and searched against the Swiss-Prot and TrEMBL sequence database (SWISS-PROT release 42.12 and TrEMBL release 25.12).

To define subfamilies we clustered the results of an all-against-all search of the 1357 HotDog domain proteins using NCBI BLASTP and single linkage clustering at an E-value of 10^-15^.

### Operon analysis

Gene context/operon analysis was carried out with the GeConT tool (Gene Context Tool) [65] available at the GeConT Home Page [66].

### Domain analysis

Protein domain analysis was carried out using Pfam [13] (release 12.0) available at the Pfam Home Page [67].

### Motif analysis

Consensus motif sequences were identified in the subfamily alignments using the MASIA program [62] available at the MASIA 2.0 Home Page [68].

## Authors' contributions

AB conceived the study and was involved in all stages of the manuscript preparation including figure preparation. SCD conducted the PSI-BLAST searches, prepared the manuscript and some of the figures. Both authors read and approved the final manuscript.

## Supplementary Material

Additional File 1 **A network showing the unification of the HotDog superfamily using PSI-BLAST searches. **Each yellow oval represents a query sequence used to seed a PSI-blast search. We compared each PSI-BLAST output to all the others and connected them with a line if they shared any sequences in common. There are only two connections in the graph that were not made (Image is in EPS format). Click here for file

Additional File 2 **A complete list of the 1357 HotDog domain containing proteins.** Click here for file

Additional File 3 **The HotDog domain superfamily. **This xml-like file contains 1293 (95%) of the HotDog domain containing sequences, grouped into 85 clusters, which permits investigators to immediately identify HotDog domain(s) in their 'unknown' protein of interest and allow them to infer some functionality. Click here for file

Additional File 4 **A HotDog domain HMM library. **This library can be used in conjunction with the HMMER program to search for HotDog domains in any protein sequence. Click here for file

Additional File 5 **A list of motifs identified in each subfamily. **Motifs were identified using the MASIA program [62]. A motif starts when at least 3 of 4 consecutive positions are more than 40% conserved and extend until at least 2 amino acids in a row are less than 40% conserved [71]. Motifs corresponding to PROSITE motif PS01328, [QR]-[IV]-x(4)-[TC]-D-x(2)-G [IV]-V-x-[HF]-x(2)-[FY] are underlined and in bold. Motifs highlighted in red and green are conserved between the respective subfamilies. Click here for file

Additional File 6 **Subfamily alignments. **These alignments were constructed using the MAFFT alignment program [72] and rendered using the CHROMA software package [73]. Known active site residues are indicated below the subfamily alignments. The highly conserved Asp residue in the PaaI subfamily is proposed as an active site residue based on motif similarities between the 4HBT-II subfamily and the PaaI subfamily. Jpred predicted consensus secondary structures are indicated above the alignments [74]. Click here for file

## Figures and Tables

**Table 1:** Classifying the HotDog superfamily into subfamilies.

**Number of Members**	**Phyletic distribution**	**Subfamily Name**	**Representative member(s)**	**Accession Number**	**E.C. number**	**Associated Domain(s)**	**General Function**
135	Proteobacteria : 67	Acyl-CoA thioesterases	Cytosolic long-chain acyl-CoA thioester hydrolase/ Brain acyl-CoA hydrolase	Q64559	3.1.2.2	2 × HotDog	Fatty acid metabolism
	Metazoa: 25						
	Firmicutes: 16						
	Archaea: 9						
	Actinobacteria: 7		Cytoplasmic acetyl-CoA hydrolase (including brown fat inducible thioesterase)	Q8WYK0	3.1.2.1	2 × HotDog, START	Fatty acid metabolism
	Chlamydiae: 4						
	Viridiplantae: 2						
	Fungi: 2						
	Deinococcus-Thermus: 2		Acyl-CoA hydrolase	Q81EE4	3.1.2.20	None	Fatty acid metabolism
	Chlorobi: 2		Probable medium chain acyl-CoA hydrolase	Q7NUH6	3.1.2.19	None	Fatty acid metabolism

130	Proteobacteria: 61	FabZ-like Dehydratases	(3R)-hydroxymyristoyl – (acyl-carrier-protein) dehydratase/ β-hydroxy-acyl ACP dehydratase (FabZ)	P94584	4.2.1.58	Lpx C in Q8KBX0	Fatty acid biosynthesis, lipid A biosynthesis (Q8KBX0)
	Firmicutes: 36						
	Cyanobacteria: 9						
	Chlamydiae: 4		Coronafacic acid (CFA) dehydratase	P72238	4.2.1-	None	CFA biosynthesis
	Alveolata: 4						
	Viridiplantae: 4						
	Planctomycetes: 3						
	Bacteroidetes: 2						
	Fusobacteria: 2						
	Aquificae: 1						
	Chlorobi: 1						
	Deinococcus-Thermus: 1						
	Metazoa: 1						
	Thermotogae: 1						

122	Proteobacteria: 81	MaoC dehydratase-like	(R) specific enoyl-CoA hydratase (phaJ)	Q8KRE2	4.2.1.-	None	PHA biosynthesis
	Firmicutes: 13		PHA synthase (phaC)	O32472			
	Archaea: 11		MaoC protein	P77455		None	PHA biosynthesis
	Actinobacteria: 8		17-beta-hydroxysteroid dehydrogenase	P51659		Aldehyde Dehydrog.	PHA biosynthesis
	Deinococcus-Thermus: 2		type 4			ADH short, SCP	Hormone biosynthesis
	Fusobacteria: 2						
	Spirochaetes: 2						
	Acidobacteria: 1						
	Planctomycetes: 1						
	Viridiplantae: 1						

32	Proteobacteria: 23	NodN-like	Nodulation protein N	P25200		None	Nodule formation
	Actinobacteria: 5						
	Bacteroidetes: 2						
	Deinococcus-Thermus: 1						
	Spirochaetes: 1						

102	Proteobacteria: 70	YbgC-like	YbgC protein	P44679		None	Cell envelope maintenance?
	Firmicutes: 14						
	Cyanobacteria: 6						
	Archaea: 3						
	Actinobacteria: 2						
	Planctomycetes: 2						
	Spirochaetes: 2						
	Aquificae: 1						
	Deinococcus-Thermus: 1						
	Metazoa: 1						

77	Proteobacteria: 36	FabA-like dehydratases/ synthases	β-hydroxydecanoyl ACP dehydratase (FabA)	P18391	4.2.1.60	None	Unsaturated fatty acid biosynthesis
	Actinobacteria: 26		Omega-3 polyunsaturated fatty acid synthase (pfaC)	Q93CG6		2 × HotDog, various numbers of BKAS N-term, BKAS-C-term & Acyl-transf. in Q93CG6, Q8YWH0, and Q9S1Z9	Polyunsaturated fatty acid biosynthesis
	Fungi: 13		Fatty acid synthase	Q48926		Acyl-transf., BKAS-N & BKAS-C	Fatty acid biosynthesis
	Cyanobacteria: 1						
	Stramenopiles: 1						

73	Viridiplantae: 57	Fat subfamily	FatA acyl-ACP-thioesterase	Q43718	3.1.2.14	None	Fatty acid synthesis
	Firmicutes: 15		FatB acyl-ACP-thioesterase	Q41634	3.1.2.14	None	Fatty acid synthesis
	Bacteroidetes: 1						

66	Proteobacteria: 31	TesB-like	*E. coli *acyl-CoA thioesterase II (tesB)	P23911	3.1.2.-	2 × HotDog, cNMP in Q8GYW7	Fatty acid metabolism
	Actinobacteria: 14						
	Metazoa: 14		Human thioesterase II/ Peroxisomal acyl-CoA thioesterase	O15261	3.1.2.2	2 × HotDog	Fatty acid metabolism. Role in HIV infection?
	Viridiplantae: 4						
	Fungi: 3						

59	Proteobacteria: 30	4HBT-II	4-hydroxybenzoyl-CoA thioesterase from *Arthrobacter *sp. strain SU and TM1	Q04416		HAD domain in Q89YN2	4-chlorobenzoate degradation
	Firmicutes: 12						
	Actinobacteria: 11		ComA2 protein	P14205		None	Unknown
	Bacteroidetes: 2						
	Viridiplantae: 2						
	Chlorobi: 1						
	Deinococcus-Thermus: 1						

21	Firmicutes: 21	CBS-associated	Hypothetical protein bh3175	Q9K832		DRTGG, 2 × CBS	Unknown

19	Proteobacteria: 10	PaaI	Phenylacetic acid degradation protein I	P76084		None	Phenylacetic acid metabolism
	Archaea: 5						
	Actinobacteria: 2						
	Bacteroidetes: 1						
	Firmicutes: 1						

14	Proteobacteria: 8	Hydroxyacyl-CoA dehydrogenase-associated	*A. tumefaciens *C58 3-hydroxyacyl-CoA dehydrogenase	Q8UJY0		3HCDH_N, 3HCDH in Q8UJY0 and Q92NF5	Fatty acid metabolism
	Firmicutes: 5						
	Actinobacteria: 1						

13	Proteobacteria: 10	Acetyltransferase	*E. coli *YiiD protein	P32148		acetyltransf	Putative acetyltransferase
	Chlorobi: 1						
	Cyanobacteria: 1						
	Metazoa: 1						

11	Firmicutes: 11	FapR	Transcription factor FapR	Q9KA00		HTH	Transcriptional regulation of fatty acid metabolism

11	Metazoa: 10 Proteobacteria: 1	MSCP	Mesenchymal stem cell protein DSCD75	Q9NYI2		None	Unknown

10	Proteobacteria: 10	YbaW	Hypothetical protein ybaW	P77712		None	Unknown

9	Proteobacteria: 9	AMP-binding subfamily	Hypothetical protein RSp0367	Q8XSV0		2 × AMP-bind	Unknown

5	Proteobacteria: 4 Firmicutes: 1	4HBT-I	4-hydroxybenzoyl-CoA thioesterase from *Pseudomonas *sp. strain CBS-3 and DJ-12	P56653	3.1.2.23	None	4-chlorobenzoate degradation
			Gentisyl-CoA thioesterase	Q9KBC9		None	Degradation of aromatic compounds?

## References

[B1] Leesong M, Henderson BS, Gillig JR, Schwab JM, Smith JL (1996). Structure of a dehydratase-isomerase from the bacterial pathway for biosynthesis of unsaturated fatty acids: two catalytic activities in one active site. Structure.

[B2] Benning MM, Wesenberg G, Liu R, Taylor KL, Dunaway-Mariano D, Holden HM (1998). The three-dimensional structure of 4-hydroxybenzoyl-CoA thioesterase from Pseudomonas sp. Strain CBS-3. J Biol Chem.

[B3] Thoden JB, Zhuang Z, Dunaway-Mariano D, Holden HM (2003). The structure of 4-hydroxybenzoyl-CoA thioesterase from arthrobacter sp. strain SU. J Biol Chem.

[B4] Zhuang Z, Song F, Takami H, Dunaway-Mariano D (2004). The BH1999 protein of Bacillus halodurans C-125 is gentisyl-coenzyme A thioesterase. J Bacteriol.

[B5] Li J, Derewenda U, Dauter Z, Smith S, Derewenda ZS (2000). Crystal structure of the Escherichia coli thioesterase II, a homolog of the human Nef binding enzyme. Nat Struct Biol.

[B6] Altschul SF, Madden TL, Schaffer AA, Zhang J, Zhang Z, Miller W, Lipman DJ (1997). Gapped BLAST and PSI-BLAST: a new generation of protein database search programs. Nucleic Acids Res.

[B7] Boeckmann B, Bairoch A, Apweiler R, Blatter MC, Estreicher A, Gasteiger E, Martin MJ, Michoud K, O'Donovan C, Phan I, Pilbout S, Schneider M (2003). The SWISS-PROT protein knowledgebase and its supplement TrEMBL in 2003. Nucleic Acids Res.

[B8] Hisano T, Tsuge T, Fukui T, Iwata T, Miki K, Doi Y (2003). Crystal structure of the (R)-specific enoyl-CoA hydratase from Aeromonas caviae involved in polyhydroxyalkanoate biosynthesis. J Biol Chem.

[B9] Thoden JB, Holden HM, Zhuang Z, Dunaway-Mariano D (2002). X-ray crystallographic analyses of inhibitor and substrate complexes of wild-type and mutant 4-hydroxybenzoyl-CoA thioesterase. J Biol Chem.

[B10] Yee A, Pardee K, Christendat D, Savchenko A, Edwards AM, Arrowsmith CH (2003). Structural proteomics: toward high-throughput structural biology as a tool in functional genomics. Acc Chem Res.

[B11] Bertone P, Gerstein M (2001). Integrative data mining: the new direction in bioinformatics. IEEE Eng Med Biol Mag.

[B12] Chance MR, Bresnick AR, Burley SK, Jiang JS, Lima CD, Sali A, Almo SC, Bonanno JB, Buglino JA, Boulton S, Chen H, Eswar N, He G, Huang R, Ilyin V, McMahan L, Pieper U, Ray S, Vidal M, Wang LK (2002). Structural genomics: a pipeline for providing structures for the biologist. Protein Sci.

[B13] Bateman A, Coin L, Durbin R, Finn RD, Hollich V, Griffiths-Jones S, Khanna A, Marshall M, Moxon S, Sonnhammer EL, Studholme DJ, Yeats C, Eddy SR (2004). The Pfam protein families database. Nucleic Acids Res.

[B14] Andreeva A, Howorth D, Brenner SE, Hubbard TJ, Chothia C, Murzin AG (2004). SCOP database in 2004: refinements integrate structure and sequence family data. Nucleic Acids Res.

[B15] HMMER: profile HMMs for protein sequence analysis. http://hmmer.wustl.edu.

[B16] Hunt MC, Alexson SE (2002). The role Acyl-CoA thioesterases play in mediating intracellular lipid metabolism. Prog Lipid Res.

[B17] Adams SH, Chui C, Schilbach SL, Yu XX, Goddard AD, Grimaldi JC, Lee J, Dowd P, Colman S, Lewin DA (2001). BFIT, a unique acyl-CoA thioesterase induced in thermogenic brown adipose tissue: cloning, organization of the human gene and assessment of a potential link to obesity. Biochem J.

[B18] Ponting CP, Aravind L (1999). START: a lipid-binding domain in StAR, HD-ZIP and signalling proteins. Trends Biochem Sci.

[B19] Heath RJ, Rock CO (1996). Roles of the FabA and FabZ beta-hydroxyacyl-acyl carrier protein dehydratases in Escherichia coli fatty acid biosynthesis. J Biol Chem.

[B20] Rangaswamy V, Mitchell R, Ullrich M, Bender C (1998). Analysis of genes involved in biosynthesis of coronafacic acid, the polyketide component of the phytotoxin coronatine. J Bacteriol.

[B21] Rangaswamy V, Jiralerspong S, Parry R, Bender CL (1998). Biosynthesis of the Pseudomonas polyketide coronafacic acid requires monofunctional and multifunctional polyketide synthase proteins. Proc Natl Acad Sci U S A.

[B22] Park SJ, Lee SY (2003). Identification and characterization of a new enoyl coenzyme A hydratase involved in biosynthesis of medium-chain-length polyhydroxyalkanoates in recombinant Escherichia coli. J Bacteriol.

[B23] Anderson AJ, Haywood GW, Dawes EA (1990). Biosynthesis and composition of bacterial poly(hydroxyalkanoates). Int J Biol Macromol.

[B24] Penning TM, Bennett MJ, Smith-Hoog S, Schlegel BP, Jez JM, Lewis M (1997). Structure and function of 3 alpha-hydroxysteroid dehydrogenase. Steroids.

[B25] Leenders F, Dolez V, Begue A, Moller G, Gloeckner JC, de Launoit Y, Adamski J (1998). Structure of the gene for the human 17beta-hydroxysteroid dehydrogenase type IV. Mamm Genome.

[B26] Baev N, Schultze M, Barlier I, Ha DC, Virelizier H, Kondorosi E, Kondorosi A (1992). Rhizobium nodM and nodN genes are common nod genes: nodM encodes functions for efficiency of nod signal production and bacteroid maturation. J Bacteriol.

[B27] Zhuang Z, Song F, Martin BM, Dunaway-Mariano D (2002). The YbgC protein encoded by the ybgC gene of the tol-pal gene cluster of Haemophilus influenzae catalyzes acyl-coenzyme A thioester hydrolysis. FEBS Lett.

[B28] Sturgis JN (2001). Organisation and evolution of the tol-pal gene cluster. J Mol Microbiol Biotechnol.

[B29] Smith S (1994). The animal fatty acid synthase: one gene, one polypeptide, seven enzymes. Faseb J.

[B30] Raetz CR, Dowhan W (1990). Biosynthesis and function of phospholipids in Escherichia coli. J Biol Chem.

[B31] Hopwood DA, Sherman DH (1990). Molecular genetics of polyketides and its comparison to fatty acid biosynthesis. Annu Rev Genet.

[B32] Metz JG, Roessler P, Facciotti D, Levering C, Dittrich F, Lassner M, Valentine R, Lardizabal K, Domergue F, Yamada A, Yazawa K, Knauf V, Browse J (2001). Production of polyunsaturated fatty acids by polyketide synthases in both prokaryotes and eukaryotes. Science.

[B33] Allen EE, Bartlett DH (2002). Structure and regulation of the omega-3 polyunsaturated fatty acid synthase genes from the deep-sea bacterium Photobacterium profundum strain SS9. Microbiology.

[B34] Fernandes ND, Kolattukudy PE (1996). Cloning, sequencing and characterization of a fatty acid synthase-encoding gene from Mycobacterium tuberculosis var. bovis BCG. Gene.

[B35] Jones A, Davies HM, Voelker TA (1995). Palmitoyl-acyl carrier protein (ACP) thioesterase and the evolutionary origin of plant acyl-ACP thioesterases. Plant Cell.

[B36] Ohlrogge JB, Jaworski JG (1997). Regulation of Fatty Acid Synthesis. Annu Rev Plant Physiol Plant Mol Biol.

[B37] Salas JJ, Ohlrogge JB (2002). Characterization of substrate specificity of plant FatA and FatB acyl-ACP thioesterases. Arch Biochem Biophys.

[B38] Naggert J, Narasimhan ML, DeVeaux L, Cho H, Randhawa ZI, Cronan JE, Green BN, Smith S (1991). Cloning, sequencing, and characterization of Escherichia coli thioesterase II. J Biol Chem.

[B39] Liu LX, Margottin F, Le Gall S, Schwartz O, Selig L, Benarous R, Benichou S (1997). Binding of HIV-1 Nef to a novel thioesterase enzyme correlates with Nef-mediated CD4 down-regulation. J Biol Chem.

[B40] Jones JM, Nau K, Geraghty MT, Erdmann R, Gould SJ (1999). Identification of peroxisomal acyl-CoA thioesterases in yeast and humans. J Biol Chem.

[B41] Cohen GB, Rangan VS, Chen BK, Smith S, Baltimore D (2000). The human thioesterase II protein binds to a site on HIV-1 Nef critical for CD4 down-regulation. J Biol Chem.

[B42] Zhuang Z, Gartemann KH, Eichenlaub R, Dunaway-Mariano D (2003). Characterization of the 4-hydroxybenzoyl-coenzyme A thioesterase from Arthrobacter sp. strain SU. Appl Environ Microbiol.

[B43] Todd AE, Orengo CA, Thornton JM (2001). Evolution of function in protein superfamilies, from a structural perspective. J Mol Biol.

[B44] Solomon JM, Lazazzera BA, Grossman AD (1996). Purification and characterization of an extracellular peptide factor that affects two different developmental pathways in Bacillus subtilis. Genes Dev.

[B45] Weinrauch Y, Guillen N, Dubnau DA (1989). Sequence and transcription mapping of Bacillus subtilis competence genes comB and comA, one of which is related to a family of bacterial regulatory determinants. J Bacteriol.

[B46] Ferrandez A, Minambres B, Garcia B, Olivera ER, Luengo JM, Garcia JL, Diaz E (1998). Catabolism of phenylacetic acid in Escherichia coli. Characterization of a new aerobic hybrid pathway. J Biol Chem.

[B47] Schujman GE, Paoletti L, Grossman AD, de Mendoza D (2003). FapR, a bacterial transcription factor involved in global regulation of membrane lipid biosynthesis. Dev Cell.

[B48] Klages U, Markus A, Lingens F (1981). Degradation of 4-chlorophenylacetic acid by a Pseudomonas species. J Bacteriol.

[B49] Haggblom MM (1992). Microbial breakdown of halogenated aromatic pesticides and related compounds. FEMS Microbiol Rev.

[B50] Dunaway-Mariano D, Babbitt PC (1994). On the origins and functions of the enzymes of the 4-chlorobenzoate to 4-hydroxybenzoate converting pathway. Biodegradation.

[B51] Chae JC, Kim Y, Kim YC, Zylstra GJ, Kim CK (2000). Genetic structure and functional implication of the fcb gene cluster for hydrolytic dechlorination of 4-chlorobenzoate from Pseudomonas sp. DJ-12. Gene.

[B52] Gescher J, Zaar A, Mohamed M, Schagger H, Fuchs G (2002). Genes coding for a new pathway of aerobic benzoate metabolism in Azoarcus evansii. J Bacteriol.

[B53] Zhuang Z, Song F, Zhang W, Taylor K, Archambault A, Dunaway-Mariano D, Dong J, Carey PR (2002). Kinetic, Raman, NMR, and site-directed mutagenesis studies of the Pseudomonas sp. strain CBS3 4-hydroxybenzoyl-CoA thioesterase active site. Biochemistry.

[B54] Bateman A (1997). The structure of a domain common to archaebacteria and the homocystinuria disease protein. Trends Biochem Sci.

[B55] Scott JW, Hawley SA, Green KA, Anis M, Stewart G, Scullion GA, Norman DG, Hardie DG (2004). CBS domains form energy-sensing modules whose binding of adenosine ligands is disrupted by disease mutations. J Clin Invest.

[B56] Birktoft JJ, Holden HM, Hamlin R, Xuong NH, Banaszak LJ (1987). Structure of L-3-hydroxyacyl-coenzyme A dehydrogenase: preliminary chain tracing at 2.8-A resolution. Proc Natl Acad Sci U S A.

[B57] Marcotte EM, Pellegrini M, Ng HL, Rice DW, Yeates TO, Eisenberg D (1999). Detecting protein function and protein-protein interactions from genome sequences. Science.

[B58] Enright AJ, Iliopoulos I, Kyrpides NC, Ouzounis CA (1999). Protein interaction maps for complete genomes based on gene fusion events. Nature.

[B59] Coggins BE, Li X, McClerren AL, Hindsgaul O, Raetz CR, Zhou P (2003). Structure of the LpxC deacetylase with a bound substrate-analog inhibitor. Nat Struct Biol.

[B60] Sali A (1999). Functional links between proteins. Nature.

[B61] Doolittle RF (1999). Do you dig my groove?. Nat Genet.

[B62] Zhu H, Schein CH, Braun W (2000). MASIA: recognition of common patterns and properties in multiple aligned protein sequences. Bioinformatics.

[B63] Hulo N, Sigrist CJ, Le Saux V, Langendijk-Genevaux PS, Bordoli L, Gattiker A, De Castro E, Bucher P, Bairoch A (2004). Recent improvements to the PROSITE database. Nucleic Acids Res.

[B64] Sharma SK, Kapoor M, Ramya TN, Kumar S, Kumar G, Modak R, Sharma S, Surolia N, Surolia A (2003). Identification, characterization, and inhibition of Plasmodium falciparum beta-hydroxyacyl-acyl carrier protein dehydratase (FabZ). J Biol Chem.

[B65] Ciria R, Abreu-Goodger C, Morett E, Merino E (2004). GeConT: gene context analysis. Bioinformatics.

[B66] GeConT Home Page. http://www.ibt.unam.mx/biocomputo/gecont.html.

[B67] Pfam Home Page. http://www.sanger.ac.uk/Software/Pfam/.

[B68] MASIA 2.0 Home Page. http://129.109.59.108/masia/.

[B69] Kraulis P (1991). MOLSCRIPT: a program to produce both detailed and schematic plots of protein structures. J Appl Crystallogr.

[B70] Merritt EA, Bacon DJ (1997). Raster3D: Photorealistic Molecular Graphics. Methods Enzymol.

[B71] Schein CH, Ozgun N, Izumi T, Braun W (2002). Total sequence decomposition distinguishes functional modules, "molegos" in apurinic/apyrimidinic endonucleases. BMC Bioinformatics.

[B72] Katoh K, Misawa K, Kuma K, Miyata T (2002). MAFFT: a novel method for rapid multiple sequence alignment based on fast Fourier transform. Nucleic Acids Res.

[B73] Goodstadt L, Ponting CP (2001). CHROMA: consensus-based colouring of multiple alignments for publication. Bioinformatics.

[B74] Cuff JA, Barton GJ (2000). Application of multiple sequence alignment profiles to improve protein secondary structure prediction. Proteins.

