# Supplementary material for: The Hotdog fold: wrapping up a superfamily of thioesterases and dehydratases
Source: BMC Bioinformatics. 2004 Aug 12;5:109. doi: 10.1186/1471-2105-5-109 (PMC516016; doi:10.1186/1471-2105-5-109)
Supplement: Additional File 2 — A complete list of the 1357 HotDog domain containing proteins. [file 1471-2105-5-109-S2.doc]

Q889K7

Q7MXT8

Q9HZX5

Q9PEI4

Q8ERN5

Q8EZ71

Q9ABN6

Q9LBK1

Q9LBK2

Q87W68

Q92JQ8

Q7W9W5

P41903

Q7WN88

Q9VXJ0

Q88QB4

Q82LU6

P95378

Q9XH14

Q9HR42

Q83EF0

Q889L5

Q8LBU6

Q92NF5

P49251

O24419

Q81RJ1

P71859

O67466

Q87W74

Q940V5

Q8NXF8

Q8UDX4

Q9X7E2

Q9A7Q7

Q82CR2

Q8D3U5

Q7U4T5

P54879

Q92SV8

Q826Z2

P94584

Q9K832

Q8LDP5

Q9A2B0

Q7P453

O24420

Q7N0L9

Q99Z80

Q89BK8

Q9A2B6

Q897M7

Q7TYI0

Q88DK8

O69071

P71860

Q9A2B9

P71863

Q99Z88

Q87XS6

Q9PEK7

Q7W0U5

Q8TQW4

Q8NMI7

Q99VD7

Q7S8U1

Q83M09

Q8FKA5

Q98BH8

Q7QH28

Q8Y7J6

Q9A2C0

O85402

Q7N0M7

Q83GB2

Q9ZLX8

Q81TF2

Q8TSR0

Q87Y36

Q8GWU8

Q7WE71

Q8R911

O54520

Q9KZJ2

Q87W94

Q7WE75

Q83AT3

Q8R916

Q89FA5

Q7QH30

Q9K9P3

Q8EMB1

P18391

Q9I2R0

P15050

Q8A0S9

P22414

Q9L5V7

Q89KQ3

Q7MDV5

Q8XL28

Q88C27

Q9A9N4

Q9RSU3

Q7W0W4

Q7VHS7

Q7WE86

Q8CTX3

Q8E9A3

Q7N9S1

Q9X431

Q8E9A9

Q8D0C6

Q7NVY3

O32472

Q9C7I5

Q9C7I8

P56653

Q975X5

Q9S355

Q7WE92

P34731

Q8NBN2

P45159

Q8F089

Q7VYA2

Q8E9B2

P59201

P77781

P59202

Q9JZR7

Q8VHK0

Q7NXT4

Q99RS5

Q8NJ77

Q93CG6

Q8D9H3

Q9JY79

Q8FRN6

Q93CG9

Q8FYX1

Q7WFU1

Q97U86

Q7QME7

Q88L85

Q8F090

Q838S0

Q8X5Y9

Q8EGV5

Q9RHY3

O53751

P32204

P32205

Q8YBL0

Q88FJ0

Q8FTI0

Q39402

Q8EXC5

Q89MN1

Q7VYB9

Q8YIV4

Q8ZH57

Q8GCW0

Q88C52

Q92M10

Q8L6B1

Q944P6

Q9FAY8

Q928P8

Q7W339

Q83M52

Q8XZI0

Q89DK1

Q89DK2

Q7PU54

Q7TV98

Q884C7

Q9HK12

Q7NP19

Q9HT54

Q8VHM4

Q8EKL2

Q9CBJ6

Q9CBJ7

Q8I6T4

Q8EKL5

Q9CBJ8

Q817E4

Q9ZTF7

Q9ZTF8

Q9P980

Q9ZTF9

Q7S204

Q8ZXD8

P25944

Q9XBJ7

Q9I501

P96398

O27971

Q9C9G1

Q9FPM5

Q9C9G3

Q96ZP3

Q7WLF9

Q88DR1

Q899N7

Q9UZR6

Q88MV2

Q9FNS7

Q9YF99

Q9FNS8

Q8Y5V9

O58863

Q83D31

Q8Y2A4

Q7QJ30

Q7MUH4

Q8P3T7

P76084

Q46651

Q8DZF8

Q825G1

Q817G0

Q8YIY5

Q9A2J7

Q8XBU9

Q7P2C3

Q9AE13

Q8P7I8

Q7U8Q6

Q9C3Z1

Q8BZR4

Q9L0M3

Q9L0M4

Q04416

Q9HV20

Q8R8Y9

Q54826

Q987R7

Q8ZGZ5

Q8PBH4

Q8E5S2

Q8PBH6

Q8GYW7

Q8FH48

Q9PNX0

Q8RD71

Q89SA5

Q18187

Q7WYA1

Q8FXB3

Q8TGA1

Q87AE1

Q7QJ53

Q9JR32

Q80TT9

Q8XXS0

Q8XXS1

Q7WLI3

Q899Q1

Q7WLI4

P73848

P08999

Q8FRT1

Q8FRT2

Q87FU1

Q8VHQ9

Q8Y687

Q8YU89

Q7W380

P95914

Q8LAE6

P42961

Q7NJG6

Q8D838

Q8NMS0

Q83X85

Q10856

O66120

Q8FJ05

Q8YKV8

Q8FRU2

Q9CMM9

Q09521

Q8FJ08

Q8P7L8

Q9UUG0

Q7MCD7

O28020

Q8KAL6

Q81RV8

Q7WNE6

Q7P7V9

Q89E03

Q39473

Q8EDH7

Q8XXU4

P70523

Q8FJ13

Q7U1K2

Q7U1K3

Q9V9W4

Q7U1K4

Q9V9W5

Q87QU3

Q9M2E4

Q7VV40

Q8NQI1

Q7WHW2

Q9ZKH2

Q87R49

Q42558

P25200

O67879

Q831Q6

Q9KR07

Q9AAA2

Q8KSU0

Q9DBK0

Q7WEB9

Q9LBY7

Q83TF5

Q8U8L8

Q7X2V0

Q42561

Q98L79

Q42562

Q9RUZ1

P38172

Q86HX3

Q8E5W6

Q8R234

Q23044

Q92M94

Q9L110

Q9KQR0

Q7NUH6

Q87NA4

Q8A384

Q9HX12

Q7NR35

O42484

Q7V3A9

Q8EDJ4

P70540

Q92RI6

Q42712

Q9PIM2

Q42714

Q42715

Q89MW7

Q8G3R7

Q41917

Q7VM25

Q8KNG1

Q817M3

Q8KNG2

Q7TZ62

Q8PIW1

Q9XFF9

Q8NMW3

Q97HJ7

Q7WPB9

Q9HUY0

Q7XUL4

Q9DBM3

Q9I4Y1

Q8PFB3

Q8XE53

Q87I31

Q8X0T6

Q8E9M7

O06135

P53716

Q7W716

Q9K8B6

Q8F481

Q9KLD8

Q7VUX2

Q8PML6

Q7MEB4

Q833N7

O53202

Q9A2Q8

Q8Y4C6

Q7VV79

P95029

Q9XFG9

Q9L9X2

Q974I5

Q8XFQ0

Q8TGG3

Q9HWT5

Q89E40

Q7UTC8

Q9I4Z5

Q7WB02

Q89N83

Q87HU4

Q82KK3

Q84F01

Q9SV64

Q8P0G9

O49337

Q8KEE5

Q89SH0

Q8GME1

Q982J2

Q9L0U4

Q8GME8

Q7VRD5

Q8PIY9

Q7WEF6

Q39662

Q9HX45

Q39663

Q8EDM1

P49851

Q8EDM2

Q88R33

O85848

Q89N91

Q7WAR4

Q7XT36

Q8DDS4

Q92215

Q9CC48

P96926

Q9K6J4

P96927

Q7W4Z4

P96928

Q8PVR6

Q8YHG9

Q8G9D6

Q9WZQ8

Q92TG5

Q92TG6

Q8DFN2

Q8F7P5

O06307

Q93PM8

O06163

Q835K5

Q8UJY0

Q8G456

Q9I3A4

Q82U05

Q9KAI5

Q89JF0

P34229

Q7NSS0

Q88LF3

Q8FZE5

Q8FZE6

Q8F0G4

Q7M8M9

Q8LI66

Q8FMM5

Q7SBJ0

Q9SNP5

Q931N3

Q89E75

Q89E76

Q7WJX2

Q8YJC0

Q9D5Y3

Q8FJ83

O06178

Q7WK78

Q9ZBK4

Q9KAJ9

O48568

Q7NUN7

Q9CQJ0

Q9CHF4

Q7V5A9

Q7VGI3

Q7VPM0

Q9A6J1

Q9HX74

Q8AYH1

Q88HS2

O83191

Q43718

Q827M5

Q92Q46

Q9I3C7

O54052

Q9I3C8

Q96YB6

Q88WF4

Q8FQC6

Q7QN48

Q7MAS2

O33877

Q9TM14

Q82DF3

Q88HT3

Q8DG00

Q7WEJ9

Q9ASQ1

Q9VZZ5

Q7PAB5

Q9A6K6

Q9VZZ6

Q972T9

Q7VX70

Q7VX71

Q9RYV7

Q8F684

Q9RYV8

Q96EA2

Q9CN69

Q88WG2

Q87PC5

Q8VYI3

Q8FZH2

Q88WG9

Q7P0V7

Q88R80

Q8XQU8

Q8CSP1

Q9BR14

Q9BR15

Q9SX65

Q7W6Y1

Q8D151

Q88R89

Q8D153

Q97WD2

Q7MYK0

Q8PN27

Q9KT42

Q97WD9

Q8G0E4

P77455

Q7NT60

Q8EJA8

O34835

Q886N2

Q8EHG5

Q8ZC71

Q845J0

Q8FN01

Q7MB46

Q92BD9

Q820F1

Q87V33

Q890Z0

Q43745

Q845J9

Q8D2Q4

Q8PN30

Q8XCC1

Q89WC3

Q82A34

Q9Z7Q0

P34419

Q9Z7Q3

Q9SIE3

Q8F2F0

Q8XLG8

Q9JUV2

Q04846

Q9PFA4

Q81KX8

Q55116

Q81L79

Q8X6K7

Q00706

Q8EQL3

Q8ZC81

Q93TG8

Q8MR97

Q9CHJ7

Q97QW4

Q87V41

Q8E0R0

P20378

Q87V45

Q7WWU5

Q8EXV4

Q8KRE2

Q8U968

Q7UGS9

Q8PE05

Q88EB4

Q7UEY6

Q99SF9

Q9A395

Q7U269

Q8ESG1

Q7WGH2

Q9HRM9

Q8ESG5

Q8X6L6

Q8D8A7

Q8ESG6

Q01373

Q9CHK5

Q8U1N3

Q9AJQ2

Q9AJQ4

Q7WCT6

Q8F7W5

Q8EDU6

Q8VJZ7

Q8SRT9

Q83TR9

Q87GC0

Q7SA55

Q7U1X7

Q87RA6

Q9HTH9

P08634

Q8RFA0

Q7W5A4

Q8X6M1

Q7N8N6

Q87RA9

Q88CI3

Q87EI3

Q81KZ8

Q9HQ35

Q7WPM5

Q8UF03

Q83BK2

Q7XV63

Q9RS06

Q8CAL6

Q8DSN8

Q8W583

O05729

Q89FY9

Q7W8W7

Q83IU8

Q87RB4

Q7N6U0

Q9ZW37

Q9JX28

Q9RVF5

Q8KCU2

Q9I042

Q8E6C8

Q7MM61

Q93VD8

Q87EJ6

Q82QD6

Q8DI01

Q7WZ14

P45012

Q89R76

Q8EFQ7

Q87V74

O04792

O04793

Q99UC7

O04794

Q81XT2

Q9KNJ8

Q98AG4

Q7NWP0

Q9HTJ3

P93828

Q7MYP4

Q11164

Q8XAM7

Q88NI9

Q11168

Q7NV59

Q9HZ94

Q9C2R0

Q8R9K3

Q9CQR4

Q9KGA6

Q7WD60

P04379

Q8FMV7

Q9A714

Q9RRS9

Q9RS29

Q7VEW2

Q8CSV2

Q8XYD1

Q89CF0

Q8A015

Q81C82

Q9KM09

Q9ZC37

Q89AL4

P57331

Q8XSV0

Q88AR0

P58137

Q8XSV3

Q92VL2

Q89HV5

Q8XSV7

Q7W053

Q82XP3

Q8UD92

Q8UD93

Q89CG0

Q819V3

Q8WYK0

Q8Y4Q0

Q7QFT9

Q98I24

Q8SMI2

Q8S9G4

Q8ER10

Q8P4I0

Q8G9P8

Q8ZDY8

Q982W7

Q87B86

Q8DFZ2

Q9JYQ0

Q82W63

P78616

Q8DFZ7

Q80ZW2

Q7Z8J2

Q9HTM6

Q96WR1

Q8KZ45

Q89AN9

Q11198

Q9FA61

Q7WPR2

O14734

Q7TZF8

Q9CJK9

Q9CI03

Q89I74

Q9S254

Q9RRV4

Q9LWL2

Q7VS17

Q9LWL3

Q8R690

Q82MT5

Q7U3X6

Q8CT87

Q7W7A1

Q87RG3

Q9HKJ9

Q9HPZ1

Q99ZW5

Q81JE0

Q8PUE2

P57362

Q8ZR29

Q8KCZ3

O84537

Q8WUY1

Q7WPS2

Q7WZ61

Q8CNJ9

Q8P6E8

Q94245

Q8DT37

Q8YWH0

Q8EFV9

Q7MRK3

Q89NH7

Q97LP0

Q8X8N2

Q7MQ05

Q8UIL0

O84540

Q98HV1

Q98HV2

Q8ZQT7

Q89UR5

Q81JF0

Q8DLK3

Q89UR6

O25174

P58175

Q8DAM7

Q19781

P38790

Q89I96

Q8Z8C2

Q7TTZ2

Q7TTZ3

Q82DR7

Q82DR8

Q81Y90

O53664

Q8ZAG7

Q8XYI5

Q8P176

Q9Y305

Q59497

Q8ZG61

Q83HA5

Q83HA6

Q97B23

Q7VVI6

Q937K7

Q9KRE1

Q7WPU4

Q816E7

Q7MVA3

O74793

Q7Q8E6

Q814K4

Q7NRG2

P45083

Q83KW0

Q7WF68

Q8NUS3

Q8CUV9

Q8Z6J3

Q8XYJ4

Q9HU04

Q9YE93

Q9P4D9

Q976V4

Q94IN9

Q7S2L1

Q18061

O65983

Q9RA19

Q8YEC2

Q94F93

Q93RX6

P94842

Q9S1Z9

P51659

Q8U9A2

Q7RP73

Q87TE3

Q99T03

Q8CRB5

Q9LK77

Q8ZG80

Q8KF72

Q7UDI3

Q8VQ22

Q44017

Q9RA22

P51660

Q92A62

Q9PC80

Q99NB7

Q7WQ69

O54490

Q9AB75

Q54721

Q9RZD5

P32148

Q988L8

Q88YP1

Q8YUR4

Q9CNE8

Q818B0

P07149

Q8E8G6

Q89V51

Q97AV4

Q976X1

Q8CYM5

Q8GN83

Q8E524

Q97UX9

Q93IV3

Q93J53

Q976X8

Q82FP3

Q9PP18

Q8F0Y6

Q87CZ9

Q9LX13

Q820T5

Q8XPI2

Q8NV54

Q7WBE7

Q8ABB1

Q89NM8

Q7XPS0

O30041

Q9U1Q5

Q7W5M4

Q83FK2

Q89UW3

Q83FK3

Q81AG4

Q41634

Q41635

Q93TU5

Q93J64

Q83C60

Q9KEQ1

Q8RC87

Q8YNJ6

Q965D7

Q7V0D0

O69502

O33537

Q9HTU8

Q978T4

Q88D52

Q9KIF1

Q99R98

Q8DNK9

Q8F346

Q7W3T8

Q7TW90

Q8DUU8

Q7TW91

Q8R9V5

Q820V3

Q7TW94

Q8PQX3

Q98EG9

Q39513

Q39514

Q7VXI2

Q87IK3

Q95TK5

Q9A3H0

Q8YB26

Q89WS4

Q9SMI9

Q9F9V0

Q8VQ60

Q9RW22

Q89M47

Q8RZQ0

Q8FR52

Q28956

Q8DUV0

Q9RBQ8

Q95Q68

Q19058

Q81JM8

Q89GF1

Q99UP0

Q927W9

Q8ZJR0

Q8Y1F9

Q820W7

Q89YN2

Q9L009

Q7XIL1

Q83SF2

Q9RXN1

Q7NYW6

Q9HTW8

Q8Y6V5

Q86S55

Q7MMD6

Q92L92

Q8EHY6

Q9A985

Q8PHV3

P44498

Q820X5

Q820X6

Q8Z8K8

Q81G96

Q98LS1

Q82DZ9

Q39534

Q7WMG6

P21773

O16725

P21774

Q7NBA2

Q9RKW0

Q7WIS5

Q8XTB0

Q7WGY3

Q89GH2

Q89IB9

Q9BIA5

Q8NWU9

Q8EPC7

Q9BIA9

Q9A5E4

Q8ZT73

Q97YR6

Q7U2H3

Q9HTY7

Q8E720

Q82JJ3

Q7WKN8

O65261

P96807

Q8F2Y4

Q92C71

Q9PFT5

Q7V5X4

Q7M7J1

O00154

Q7M7J5

Q89PM0

Q8R6E0

Q8RLA7

Q39554

Q64559

Q39555

Q986Y5

Q7VKU0

Q81WI4

Q7W480

Q9CB38

Q92N63

P96670

Q92N64

Q9RVW9

Q92Y49

Q9ZJL6

Q7M7K1

Q8D6Z5

Q9I0E9

Q7M7K3

Q94FB6

Q9I644

Q9HMQ9

Q88IJ9

Q8ZV39

Q7MS67

Q9RZL9

Q7U2J7

Q7VDL8

Q7VVT2

Q84FL8

Q986Z7

P38256

Q9RC57

Q7QLR0

Q7TY91

Q9JTP2

P44679

Q7VTZ9

Q93HC1

Q93HC2

Q8K631

Q8J0J9

Q87ME8

Q9HN18

Q9A5H5

Q9NYI2

Q9PJK7

O06209

Q8EM17

Q97D89

Q9KBC9

Q81LM5

Q8A611

Q7WJ67

Q9KS00

Q824Q6

P27845

Q7NVG8

Q8ZEH6

Q7QAU2

Q9FJI2

Q9HXQ3

Q9FAE9

Q9KPW3

Q9UVH9

Q8U9L0

Q9A7C4

Q7NQ84

Q45061

Q7VZJ5

Q9WWX4

Q9PJL0

Q89X90

P72238

Q9I669

Q9VEV6

Q7WBN6

Q7UFN2

Q7MS88

Q8UIZ0

Q7WA35

Q98TA2

Q7WIX8

Q9A246

Q8H6F0

Q8RP95

Q7T175

Q7MMJ7

O33906

Q9CGF4

Q7V4A7

Q97Q23

Q7N3T8

Q55777

O86577

Q81EE4

Q84HI6

Q8YD49

Q84HI7

O69594

Q8ZRB2

O06085

Q7WJ83

Q7VW65

Q7WA49

Q8YGJ2

Q9L080

Q8N1Q8

Q9XEL9

Q814Y7

Q7UJD3

Q8ZM35

Q84HJ2

Q89PR6

Q84HJ3

Q9CD22

Q84HJ7

Q7W813

Q84HJ8

O84987

Q8XF59

Q8F4Y0

Q92P52

Q97NZ8

Q82LJ9

Q7VKZ2

Q8YIE1

Q9CM67

Q7MFB9

Q7TZX6

Q88KI2

Q7NKL9

Q8DTD4

Q8EPJ1

Q8K7R4

Q9KQ95

Q8FYH7

Q7UUC7

Q89Q24

Q89Q25

Q84HK2

Q84HK3

Q87U26

Q8RLG9

Q84HK7

Q84HK8

Q7VY27

P94334

Q9I0K2

P40725

Q7SI66

Q9L245

Q8VXJ5

Q8VXJ6

Q7QM69

Q8EGG4

Q89Q33

Q8E1K3

Q7MJ83

Q84HL2

Q84HL3

Q89Z74

Q9NPJ3

Q97YZ5

Q8F6U3

Q84HL7

Q92QR3

Q84HL8

Q7U2P5

Q7VE16

Q7W836

Q7W837

Q8D063

Q7SI70

Q89KE3

Q8YIG6

Q8YIG7

Q9SQI3

Q02207

Q93JC6

Q7N470

Q8Z8U3

O25928

Q8D5F7

Q9DCP4

Q7VFM3

Q82CI1

Q7WBS0

Q84HM2

Q8XEY3

Q9FQX7

Q9FQX8

Q8UBU4

Q9ZED4

Q9FQX9

Q84HM8

Q82LM4

Q84HM9

Q7WBS9

O54133

P44886

Q8XVF7

Q8PAW4

Q8D7A8

Q7S1B2

Q9FQY0

Q9S2J8

Q9FQY1

Q9AIQ0

Q84HN3

Q84HN4

P11469

Q84HN7

Q84HN8

Q9CNU6

Q92P90

P95162

Q7U319

P23911

Q7S6R7

O29141

Q97MC7

Q86SA5

O28346

Q88MG9

Q7VUA9

Q7MU91

Q9EUT2

Q8EYR4

Q7VZQ6

P97852

Q8RJQ5

Q7U2S5

Q7WBU8

Q8FV79

Q8FK97

Q9KA00

Q7MMQ2

Q8G1C7

Q9LQ87

O26754

Q8EGK2

Q8UFL4

Q8IG45

Q9HXY7

Q84HP5

Q84HP6

Q7UG50

Q93DX2

Q8E045

O66468

Q84HP9

Q9R0X4

P87304

Q8NTL7

Q8E989

Q97DA9

Q7NVP2

O34921

Q7NVP3

Q8WXI4

Q98H00

Q7NJ71

Q7VSI2

Q8KY19

Q44631

Q7XXM7

O68070

Q85G00

Q7UJK5

Q92SQ1

Q84HQ0

Q883Y1

Q8XH68

Q7W9S4

Q8FWT6

Q9FI76

Q8XH69

Q8DPV2

Q883Y6

Q7WC65

Q9HUE3

Q7U343

Q8CRV5

Q9X5F5

Q83HW6

Q7W4D3

Q9KL09

Q48926

Q8FPK1

Q8K9S4

Q96KR2

Q7TYD8

Q8A2G2

Q8PGJ2

Q92AJ8

Q8Y259

Q98F79

Q91V12

Q8EZ46

Q8EYU8

Q7U352

Q87TZ7

Q8CS64

Q98KG3

Q82B35

Q9SJE2

Q97VK2

Q05747

Q99Q03

P42398

O97376

Q8II11

Q89IQ0

Q8KBX0

Q8ZPQ8

Q8NPZ7

Q8YPW1

Q8XU05

Q9H2R8

Q92UM3

O07408

Q7TWK5

O85079

Q7WN64

Q98BD1

Q7UFY0

Q7WMW8

Q8RT81

P77712

Q99TF8

Q828Q9

O29336

Q88FB7

Q88FB8

Q8NS46

P14205

Q8PCW9

Q9CTI2

Q7NTY2

Q8XTR6

Q8FZ03

Q7TYF8

Q7W0R1

Q9W439

Q7WFN8

Q7V171

Q9HLD5

Q98MC5

Q8S392

O70529

Q88FC4

Q8E089

Q8DBF0

Q9W440
