# Supplementary material for: The Hotdog fold: wrapping up a superfamily of thioesterases and dehydratases
Source: BMC Bioinformatics. 2004 Aug 12;5:109. doi: 10.1186/1471-2105-5-109 (PMC516016; doi:10.1186/1471-2105-5-109)
Supplement: Additional File 5 — A list of motifs identified in each subfamily. Motifs were identified using the MASIA program [62]. A motif starts when at least 3 of 4 consecutive positions are more than 40% conserved and extend until at least 2 amino acids in a row are less than 40% conserved [71]. Motifs corresponding to PROSITE motif PS01328, [QR]-[IV]-x(4)-[TC]-D-x(2)-G [IV]-V-x-[HF]-x(2)-[FY] are underlined and in bold. Motifs highlighted in red and green are conserved between the respective subfamilies. [file 1471-2105-5-109-S5.DOC]

| **Subfamily** | **MASIA consensus HotDog domain motif(s)** |
| --- | --- |
| Acyl-CoA thioesterases | FGG-x(2)-M-x(2)-D |
| FabZ-like Dehydratases | 1) HRYPFLLVD, 2) KNVT-x-NE, 3) HFPP-x-MPGVLI-x-EAMAQ |
| MaoC dehydratase-like | None |
| NodN-like | 1) FADAT-x-DHQWIHVDPERAA, 2) PFG-x-TIAHGFLTLSLLP, 3) NYG-x-DKVRF-x(2)-PV-x(2)-GSRVR, 3) TVEIEG-x(2)-KPA-x-VAE |
| YbgC-like | DT**D-x(2)-GVV-x-H-x(2)-Y** |
| FabA-like  dehydratases/  synthases | 1) MLM-x-DRI,  2) AELDI-x-PDLWFF-x-CHF-x-GDPVMPGCLGLDAMWQL-x-GF-x-LGW-x-G  3) RALG-x-GEVKF-x-GQVLP |
| Fat subfamily | 1) LIWVV-x(2)-M-x-I, 2) DLD-x-NQHVNNVKYIGW-x-LES |
| TesB-like | 1) VFGGQ, 2) GQAL-x-AA, 3) VHSLH-x-YFLR-x-GD, 4) VER-x-RDG-x-SFS-RRV-x-A-x-Q-x-G, 5) LAY-x-SD, 6) ASLDHSMWFH, 7) QEG-x(2)-R |
| 4HBT-II | 1) MPVD-x-RT-x-QPFG-x-LHGGAS-x-LAE, 2) VGLE-x-NANH-x-RS, 3) TQVW-x-I-x-I-x-DE |
| CBS-associated | 1) GVF-x-IL, 2) LIIEQ-x(2)-IYFLPVQ, 3) GRR-x(2)-ID |
| PaaI | 1) HGG-x-IF-x-LAD, 2) GRTG-x-YDV-x-V-N-x(2)-G-x-LVA-x-FRG-x-SYR |
| Hydroxyacyl-CoA dehydrogenase-associated | WIDYNGHM-x-DA-x-Y |
| Acetyltransferase | 1) SEKMGV-x-I-x-QYTGQ-x-F, 2) APL-x-PN-x-NPH-x-T-x-FAGS, 3) LATLTGWGL-x-WL-x-L-x-ER-x-L-GDIVLAD-x(2)-IRY, 3) SGDLDRLA-x-GRKAR, 4) GTY-x-VLP |
| FapR | 1) IARGHHLFAQANSLAVAVI-x(2)-E-x-ALT, 2) GERV-x-AKA-x-V |
| MSCP | 1) LLDV-x-YF-x-R, 2) VL-x-ARLLQ-x-R, 3) HMNNARYLRE-x-DFARV-x(2)-Y-x-RTG, 4) VQGAS-x(2)-RYRR, 5) WDD-x-A-x-YLE-x-RFV-x-L-x-D, 6) GFV-x-A-x-A-x(2)-RQ |
| YbaW | 1) IKVRGYHLDVYQHVNNARYLEFLEEARW, 2) VVNININYRRPAVLGDLL, 3) ALITFVCIDLKTQKAL-x-LEGELREKLE |
| AMP-binding subfamily | 1) GHF-x(3)-PLLPGV-x-Q-x-DWA, 2) KFQ-x-P-x-LP, 3) SSGK-x(2)-L |
| 4HBT-I | 1) FGDC**DPAGIVWFPNY**FRWLDAASRHFFI-x-CG, 2) SYDDVL-x-IET-x-IREW-x-RKSF-x-Q-x-H-x-V, 3) QLVM-x-ADETRVFAG, 4) GRLRAIPIPADY |
