# Supplementary material for: The Hotdog fold: wrapping up a superfamily of thioesterases and dehydratases
Source: BMC Bioinformatics. 2004 Aug 12;5:109. doi: 10.1186/1471-2105-5-109 (PMC516016; doi:10.1186/1471-2105-5-109)
Supplement: Additional File 6 — Subfamily alignments. These alignments were constructed using the MAFFT alignment program [72] and rendered using the CHROMA software package [73]. Known active site residues are indicated below the subfamily alignments. The highly conserved Asp residue in the PaaI subfamily is proposed as an active site residue based on motif similarities between the 4HBT-II subfamily and the PaaI subfamily. Jpred predicted consensus secondary structures are indicated above the alignments [74]. [file 1471-2105-5-109-S6.DOC]

**Acyl-CoA thioesterase subfamily (135)**

Secondary St. ----------HHHHHHHHHHHHHHHHHHHHHHH-----EEEEEE----EE-------EEEEEEEEEEE------EEEEEEEEEEE------EEEEEEEEEEEEEEEEEEEEEE---

Q9V9W4_323-424 **PFP**ENR**N**AQ**NTIFGG**Y**LM**R**QA**V**EISFI**M**A**SI**Y**LGDR--**PT**LRC**IS**D**ISFM**H**PV**H**V**DR**FL**Q**L**T**A**H**V**V**YAA**---**QN**YV**QL**MTV**A**QIW**D**AKSGKV---**Q**T**TN**VF**YLTY**R------A**D**KV

Q7QFT9_355-456 **SHP**E**D**R**N**AH**NKVFGG**F**LM**RN**A**L**ELSWA**L**A**Y**NFA**KRR--**P**KLEH**IS**D**ISFH**H**PV**D**V**S**SML**N**M**Q**A**H**V**I**FTD**---L**H**Y**MEI**V**V**L**A**DVY**D**AVTGQQ---TT**TNS**F**YYTY**S------V**A**ER

Q9R0X4_299-400 **CHP**Q**E**R**N**VF**NRIFGG**F**LM**R**KA**Y**ELAWA**T**A**C**SFG**GSR--**P**Y**VV**T**VD**D**I**M**FQ**K**PV**E**VGSLL**F**L**S**S**Q**V**C**FT**Q---**DN**Y**IQV**R**VHS**EVS**S**LDSREH---**M**T**TN**VF**HFTF**M------**S**EKE

Q7Z8J2_329-434 M**QP**Q**D**R**N**LH**NNIFGG**Y**LM**R**RA**Y**ELAYA**NTAL**F**MKTSS-**PT**L**L**S**MD**E**VTFR**K**PV**H**VGTLL**N**L**R**S**G**I**V**LS**EGYP**HRS**V**QV**R**V**I**A**EVI**N**IEKGTR---**E**T**TN**VF**HFT**LA------**SG**ND

O49337_297-407 **CQP**QQR**N**IH**GRIFGG**F**LM**R**KA**F**ELAF**SN**A**Y**TFA**GVS--**P**RF**L**E**VD**R**VDFI**K**PV**D**VGNFL**R**F**K**S**R**V**L**YT**EATS**S**AEPL**I**N**IEV**VAHVTSPELR-SS**E**V**SNR**F**YFTF**S-**V**RP**E**-AMKD

Q23044_263-368 **AQP**E**H**E**N**PY**GSVFGG**F**L**VR**KG**L**ETA**E**L**C**A**KM**FS**KTS--**V**R**V**SS**ID**D**AEFM**KV**V**E**IGSIL**K**F**S**A**F**V**CN**VD**-NK**EQ**K**FQV**NS**QV**EVY**N**SNTNKF---**EICDR**F**LFTF**E**A**KEEIN----

Q9ABN6_43-150 **AMP**S**DTN**PE**GDIFGG**W**LL**S**QMDLAAA**SI**A**F**HRA**AGR--**CATI**A**ID**G**MTFL**S**PV**F**VGD**E**V**S**L**F**A**K**V**V**HTG**---**RTSLKV**A**VEA**WRR**R**RDGEQA---N**KVTEG**V**FTFVAID**E**N**R**KS**RP

Q87U26_21-128 **ALP**R**ETN**GF**GDIFGG**W**L**VS**QMDLAGTA**M**A**S**K**V**A**GGR--**VATV**A**ID**R**M**A**FL**V**PV**A**VG**EQ**L**S**F**YTR**T**L**EVG**---**RTSIK**MM**VEV**WSD**D**PVTSEW---**RKVTEA**A**FVFVAID**S**S**G**RT**RS

Q89SH0_26-133 **AMP**A**DTN**AN**GDIFGG**W**LL**S**QMD**V**GGGV**F**A**S**K**V**A**KSR--**TVTV**A**I**EA**MNFR**KA**V**Y**VGDLV**S**V**Y**A**N**L**V**RVG**---**RTSLTV**H**LEA**WAL**R**RREADP---**FLVTDG**N**FTYVSID**D**H**G**RP**QS

P25944_18-128 **AMP**A**DTN**AN**GDIFGG**W**LM**S**QMDIGGAI**L**A**K**EIA**HGR--**VVTV**R**V**EG**MTFL**R**PV**A**VGDVV**C**C**Y**A**R**C**V**K**R**G**---**TTSISI**N**IEV**WVK**K**VASEPIGQR**YKATEALF**I**YVAVD**P**D**G**KP**RP

Q7W4D3_24-132 **PMP**A**DAN**IH**GDVFGG**W**IM**A**QVDIAG**S**I**P**A**A**RRA**AGR--**VATV**A**VN**A**FQFK**Q**PV**F**VGDLL**S**F**YTS**I**T**RTG**---**TTS**V**TV**S**VEV**YAE**R**QRLDAE--VV**KVTEA**V**LTYVATD**EAR**RS**RP

Q8UDX4_18-125 **AMP**A**DAN**PA**GDIFGG**W**VM**S**QMDLASGI**R**A**A**ERS**RCR--**VVTA**A**V**KE**M**A**FE**L**PV**K**IGDTL**S**I**YTD**I**A**R**L**G**---**RTSITL**T**VEA**WAQ**R**SRYDKL---**EKVT**A**G**T**F**IM**VAMD**E**N**G**QP**TP

Q9PP18_13-123 **AMP**S**DTN**PA**GNIFGG**W**IL**S**QIDLAGAI**A**A**R**ELS**PER--**VVTI**S**MD**K**V**V**FK**E**PV**F**IGDII**S**C**Y**S**K**V**VN**VG**---**NTSISV**E**VEV**TAQ**R**VDSQGCTSC**I**N**VTSAL**V**TYVSVT**R**D**G**K**KNP

Q7NUH6_15-121 **ALP**TS**TN**AY**GRV**QA**G**W**LM**S**QIDMAG**S**L**D**A**E**RLS**RGP--**VTTV**A**VN**A**FQF**AA**PI**L**LGDVV**D**M**YVERL**R**I**G**---**QKSITL**K**ISV**EAE**R**MDGSHV----**R**I**TEVI**A**TFVAVD**A**E**G**KS**RL

O66120_26-133 **AMP**AN**TN**AD**GRMFGG**W**LM**G**MLDQAAGL**V**A**A**RHA**LAR--**VVTV**A**AD**S**ITFH**A**PV**Q**VGD**E**L**S**L**Y**A**R**L**V**KVG**---**RTSMKI**E**VEG**WRRVRHELET---**IKA**I**SGLFTFVAID**E**D**R**RP**CQ

Q64559_192-301 **V**G**P**S**DC**TLH**G**F**VHGG**VT**M**K**LMDEVAGI**V**A**A**RHC**KTN--I**VTA**S**VD**A**INFH**DK**I**RK**GCVI**T**I**S**G**R**M**T**FTS**---**NKSMEI**E**V**L**V**DADPVVDNSQ-KR**YRAASAFFTYVSLN**Q**E**G**KP**LP

Q91V12_235-344 **V**G**P**S**DC**TLH**G**F**VHGG**VT**M**K**LMDEVAGI**V**A**A**RHC**KTN--I**VTA**S**VD**A**INFH**DK**I**RK**GCVI**T**I**S**G**R**M**T**FTS**---**NKSMEI**E**V**L**V**DADPVVDNSQ-KR**YRAASAFFTYVSLN**Q**E**G**KP**MP

Q9HKJ9_17-124 **VLP**E**DM**DIYEY**LYGG**R**LM**E**WID**N**CA**S**I**V**A**T**KHC**RKR--**TVTG**S**ID**S**L**F**FL**L**PI**H**LGDMV**I**L**H**G**Y**I**N**YTT**---**KS**T**MEI**E**IDV**IKE**E**GLTGIR---**RYATKAYLTYVAID**S**D**G**RP**TE

Q7MFB9_14-121 **AEP**G**DVN**FG**GKVHGG**A**VM**K**WIDLAAYA**CSAA**WS**GKY--**C**I**TA**Y**AG**G**IRF**VA**PI**H**VGNLV**E**V**S**A**K**V**I**YTG**---**TTSMHI**A**IDV**QAS**D**PKDLKN---**RLTTHCI**VIM**VAVD**E**N**G**QP**TP

Q9RHY3_202-311 **AKP**T**DIN**WG**GNVHGG**TA**M**E**WIDEAGTA**CTM**EWS**GEQ--**T**I**AV**Y**AG**G**IRFY**K**PI**H**IGDLI**E**V**D**A**R**M**M**RTD**---**SRSMQ**MS**VHV**RSG**E**ARGGRE-NLST**A**I**HA**TV**AYLAMD**R**D**W**NP**LP

Q975X5_161-268 I**YP**E**HG**FMN**G**I**LY**A**G**KM**Y**T**MLDEA**L**AI**V**A**KL**YS**KGN--**V**F**TA**S**AG**Y**ANFL**T**PV**R**IGDIL**E**I**Q**G**A**I**E**YTG**---**NTSLDV**GA**KV**FAI**N**HYTGEK---**RLVTRT**V**FSFVAID**E**N**G**KP**KP

Q96YB6_176-285 **V**T**P**DM**T**YDGRI**I**SA**G**K**LL**K**LMD**D**IGGA**LCL**NY**ISEEGA**VVTV**S**IN**S**TSFY**T**PI**R**L**S**DII**K**I**R**A**G**I**S**YVG**---**STSFEV**I**LNV**IRL**D**PRNFVE---**EHVTTAYFNYV**R**ID**K**S**G**RP**TK

Q8ZT73_167-271 **AMP**E**D**SLDG**V**L**LYGG**K**LL**F**KLDEL**T**FV**E**A**F**SY**YPTV--Y**VTA**S**VN**S**I**V**FR**R**PI**Y**VGDIV**E**V**KTG**V**T**HVG**---**STSIEI**GFV**V**EAFGSRGR-----**RRVADGFFTFV**-NMAGG**KP**SE

Q9YF99_208-315 **V**N**P**I**D**SVAY**D**V**MH**A**G**AM**L**Y**IVDELGAV**A**A**L**R**S**S**AGI--**VVTG**F**VG**P**ADF**VA**P**MR**VG**E**VL**R**M**W**A**R**T**S**YVG**---**RSS**V**EV**L**IRS**ASGFMGGGEA---**RLTSEAYLTYISLD**S**S**G**RP**LE

Q81EE4_23-130 **VFP**T**DLN**DH**NTLFGG**K**IL**S**EMDMVA**S**I**S**A**S**RHS**RKE--**CVTA**S**MD**W**VDFL**H**PV**RSS**DCV**S**Y**E**S**F**V**I**WTG**---**RTSMEV**F**VKV**VSEYLISGEK---**RIAATSF**V**TFVALS**K**E**N**NP**VP

Q8RLA7_17-123 I**R**NS**DLN**EH**GTVYGG**R**IL**E**LID**GQ**A**S**V**A**A**M**R**V**A**RTT--**VATV**S**MD**E**IQFL**R**P**FD**L**Q**D**SMC**M**E**A**Y**V**TGF**G**---**KRSIEV**FT**KV**IGE**H**LMTGER---**FLG**FY**CFMTFV**I**LD**P**E**-**K**QTA

Q9KEQ1_18-125 **VLP**P**DTN**HL**GTIFGG**K**VL**A**YIDEIAAL**T**A**M**KHA**NSA--**VVTA**S**ID**S**VDFK**SSAT**VGDAL**E**L**E**G**F**V**T**HTG**---**RTSMEV**Y**VRV**HSN**N**LLTGER---T**LTTESFLT**M**VAVD**E**S**G**KP**KP

Q99T03_22-129 **VFP**Q**DTN**HHH**TMFGG**T**LM**AN**IDEIAAI**T**A**M**KHA**GAQ--**VVTA**S**TD**S**VDFL**K**PI**K**TGDIL**Q**Y**V**A**M**V**S**YAG**---**TSSMEV**V**VQ**IRID**D**VFNNKH---D**LAA**L**SYLTFVALD**D**E**G**KP**KH

Q9RXN1_95-202 **VFP**K**DTN**YH**GTAFGG**W**VL**A**LMDKAA**S**V**A**A**V**RHA**GGN--**VVTA**R**MD**G**VDFH**V**PI**R**VGDAV**A**L**D**A**R**V**I**RVG**---**RSSMTI**R**VDV**YRE**N**MASGEQ---**QLAT**G**GLFTFVALG**E**D**G**KP**RP

Q8U9A2_154-261 **VFP**DQ**AN**SA**GRMFGG**EA**I**A**YM**T**KAAFV**A**A**S**RYC**GKL--**VV**L**A**SSER**IDF**ARA**I**E**IG**E**IV**E**A**Q**A**H**V**E**RVG**---**RSSMSI**QT**K**LWSE**N**LLTGER---**HITATGHFT**M**VAVD**K**D**H**RP**AT

Q9HR42_18-125 L**LP**N**DTN**NL**GRA**L**GG**T**VL**H**WMDICAAI**ASM**RFA**GSQ--**CVTA**S**MD**H**VDFI**TA**I**E**MG**E**V**AV**V**E**A**Y**V**FD**TG**---**STSIDV**K**VDV**HAE**D**PREGAT---**RKTTSSFFTFVAVD**D**D**G**SP**IP

Q9HMQ9_15-122 **VQP**D**DTN**NY**ASAHGG**N**V**VK**WMDEIGAM**A**A**M**RHA**GKT--**CVTA**R**IN**S**LDFE**R**PV**PQ**GDI**CV**I**Q**A**Y**A**YD**TG**---**HTSIKV**R**LRA**FRE**D**PRSGET---**E**P**TTDSYFVFVAVD**D**D**M**QP**TA

Q7QLR0_10-117 **VLP**N**ETN**SLR**NLFGG**E**LL**A**RMDRCA**S**I**S**A**S**RHS**ERR--**VVTA**S**VN**H**VSF**NA**PI**PE**GSVV**V**L**E**S**K**V**S**RA**F---**STSMEV**Y**VDA**WLD**D**PIHRKK---**IHTN**A**GIYTFVAVD**EFN**KP**VP

Q88MV2_23-130 **VFP**NT**TN**HH**NTLFGG**TA**L**A**WMDEVSFI**A**A**T**RFC**RLP--L**VTV**S**TD**R**IDFK**H**PI**P**AGSIV**E**L**V**G**T**V**I**KVG**---**NTSLQV**Q**VDV**FVE**N**MYLDGR---**ERA**I**HG**V**FSFVAID**E**D**K**RP**VP

Q8KEE5_7-115 **VMP**E**HLN**HY**G**F**LFGG**N**LL**K**WIDEVSYI**AVTLDYPGCN-F**VTV**G**MD**N**IKFK**KS**I**RQ**GTIL**C**F**E**S**KKN**H**I**G**---**TTS**V**E**YT**VDV**TRE**E**ISTGSR---**ELV**F**TTRITFVSVD**E**N**G**R**KKA

Q824Q6_19-126 I**FP**N**DLN**TN**NTVFGG**L**LM**S**LLDRLALV**V**A**E**RHT**EGI--**CVTA**F**VD**A**LRFY**A**P**AY**MG**EN**L**I**C**K**A**A**V**N**RT**W---**KTSLEV**G**VKV**WAE**N**IYKQER---**RH**I**TSAYFTFVSVD**E**N**NA**P**TP

Q88C27_14-121 MT**P**DM**AN**FS**GNVHGG**T**LL**K**YLDEVAYA**C**A**S**RYA**GSY--**VVTL**S**VD**Q**V**I**FR**E**PV**H**VG**E**LV**T**F**L**A**S**V**N**YTG**---**NTSMEV**G**IKV**VTE**N**IRERSV---**RHSNSCFFT**M**VAVD**D**N**R**RP**VP

Q82DF3_53-161 MTHN**DTN**LL**GTVHGG**V**IM**K**LV**VD**AAGA**V**A**G**RHS**GGP--**AVTA**S**MD**E**M**V**FL**E**PV**R**VGDLV**H**V**K**A**Q**V**N**WTG**---**RTSMEV**G**VRV**LAE**R**WNESAP--AT**QVGSAYLVF**A**AVD**A**D**G**KP**RR

Q8WYK0_189-299 **VLP**P**HAN**HH**GNTFGG**Q**IM**A**WME**T**VATI**S**A**S**RLC**WAH--**P**FLKS**VD**M**FKFR**G**P**ST**VGD**R**L**V**F**T**A**I**V**NN**T**F---**QT**CV**EV**G**VRV**EAF**D**CQEWAEGRG**RH**I**NSAFL**I**Y**N**AAD**D**K**E**N**LIT

Q8CAL6_212-320 **VLP**P**HAN**HQ**GNTFGG**Q**IM**A**WME**N**VATI**A**A**S**RLC**HAH--**PT**LKA**I**EM**FHFR**G**P**SQ**VGD**R**L**V**L**K**A**I**V**NN**A**F---**KHSMEV**G**VCV**EAY**R**QEAETQ--R**RH**I**NSAFMTFVVLD**K**D**D**QP**QK

Consensus/80% sbP.chN..sphaGG.lb.bh-bhthh.A.pbs.....sssh.hs.hpFb.Pl.hGshl.h.t.h.bss...ppSbpl.lps...p.........bbsspsbbsalshs.p.ps..

**FabZ dehydratase subfamily (130)**

Secondary St. –HHHHHHHHHH------EEEEEEEEEE-------EEEEEEE-------------------HHHHHHHHHHHHHHHHHHHH-------------EEEEEEEE----E---EE---EEEEEEEEEE------EEEEEEEEE----EEEE---------

P72238_16-153 **MGFTELK**--**TWL**R**HRHPMVYLDRVLDY**E---PGVQ**I**KT**L**M**AVSG**QTD**A**LA**GHFP**E**R**A**IYP**ASH**L**MQ**AI**S**QSAIIL**F--Q**L**STSRL------AGDE**VTLVG**SI**KSRF**T**R**P**V**V**PGD**L**V**I**FQLDC**ESL**R**-P-D**FFTF**S**C**R**A**T**VDGR**S**VG**ML**K**GSL**V**---

Q8K9S4_8-145 **FNIKDIL**--**NILPHRYPFLLIDRILDF**K---AFQY**L**K**ALKN**C**TVNE**P**FF**Q**GHF**IK**EPVFPGVLMIEAM**S**QAAAVL**---I**F**KSIGK---LNINQ-L**YYFVGIENTRFKK**I**V**V**PGD**Q**I**F**IEV**I**Y**L**K**S**K**-K-N**FIKF**KIF**A**I**VN**K**K**N**VCKST**-**I**I**F**---

Q89AN9_4-144 S**NITDI**T--**TLLPHRYPFLLIDRII**A**Y**Q---KNFN**I**LT**IKNISYSE**F**CF**T**GHF**YK**NPVFPGVLILEAIAQSACLL**---V**Y**KSFGM---SYKNN-L**FYLTNI**I**DVKFKK**K**V**I**PGD**QML**INV**F**V**D**K**H**H**-H-R**LIRF**V**G**H**V**S**VS**KYI**VCKAT**-**I**S**C**LLT

Q820W7_4-145 **MNITDIK**--**KYIPHRYPFLLIDRVIKI**E---KDKS**L**V**AIKNVTVNE**P**FF**T**GHFP**V**RPVMPGVLIIESLAQAAGIL**---I**V**KSLNLP--EGHKD-I**YFFAGVDNARFKR**V**V**E**PGD**Q**L**T**LEVKV**L**K**V**H**-R-G**LWKF**E**G**K**A**T**VDDQ**L**ACKAE**-**L**M**T**IKG

Q7VRD5_8-148 **L**H**IEEVL**--**ELLPHRFPFLLVDRVLNF**E---KGKF**L**R**A**V**KNVSFNE**P**FF**Q**GHFP**G**KPIFPGVLILEAMAQA**T**GIL**---A**F**KSMGK---LAPGE-F**YYFAAIDEARFKR**P**V**Q**PGD**QMI**LNVEF**I**K**E**R**-C-G**VARF**K**G**I**A**T**VN**E**E**M**ACEAS**-**M**M**C**ARR

Q7WA49_19-163 **LDIK**G**IM**--**D**R**LPHRYPMLLIDRVLEM**V---PGKS**I**V**AIKNVSINE**P**FF**T**GHFP**H**HPVMPGVLIVEAMAQASALF**---S**F**TDENG(4)DGAKT-A**YYLVGID**G**ARFRK**P**V**V**PGD**Q**L**R**LEVEA**E**R**LS-C-T**ICKY**Q**G**R**A**L**VDGQ**L**VAEAK**-**L**M**C**AIR

Q87W74_16-153 **MGFTELK**--**TWL**R**HRHPMVYLDRVLDY**E---PGVQ**I**KT**L**M**AVSG**QTD**A**LA**GHFP**E**R**A**IYP**ASH**L**MQ**AI**S**QSAIIL**F--Q**L**STSRL------AGDE**MTLVG**SI**KSRF**T**R**P**V**V**PGD**L**V**I**FQLDC**ESL**R**-P-D**FFTF**S**C**R**A**T**VDGR**S**VG**ML**K**GSL**V**---

Q8D063_27-174 **FG**Q**D**I**IK**--**KMIPHR**E**PFLMVD**N**VE**L**I**NI--ECRL**I**K**AI**RR**V**DEKDP**VF**G**GHFP**N**DPIYPGVLQ**Q**ESM**F**QTALIL**(8)A**I**PPAEE------NIIH**AV**G**T**R**V**Y**DV**FHL**S**A**V**R**PG**ELMT**IRC**C**I**T**E**Y**D**-T-F**LATA**I**V**QIT**VNDQ**I**VT**V**GK**-GE**F**HVF

Q8E6C8_4-156 **LS**S**EEII**--**KMLPHRYPFLMVDRVQSY**----KKGI**I**EC**RKNITINE**P**YF**Q**GHFP**Q**RPI**V**PGVLMIE**MA**AQSAALL**(8)S**L**PSLDE(4)KIAEK-V**GYLASVKN**F**KFKK**I**V**T**PGD**Q**L**N**ITCK**SQSKL-G-H**LLEI**Q**V**VIRDE**NK**KE**V**A**S**GR**M**L**V**SEN

Q9RC57_8-150 **INVTELR**PG**NML**LQQP**PFLFVDRILEF**----DEET**I**TCS**K**Y**LSHNE**P**FF**S**GHFP**T**QPIMPGVLIIE**FA**AQASLLL**---T**M**LQL-----NELEPLM**GYLV**KT**EN**FT**FH**ALAE**PG**TE**L**EA**KVKM**L**K**KMGNYY**TTQV**T**V**RRSD**N**K**K**K**VAKGQ**-**L**V**F**YLD

Q81C82_1-131 **M**H**IKD**T-----**LPHRYPFLMIDKV**T**NV**K---QDEF**V**T**GYK**L**IT**N**NE**W**F**IND---S**H**KH**MP**HM**LIVEALAQ**L**SA**F**V**---H**T**SDSEG---------L**GFL**S**SLD**G**V**E**FH**GKAY**PGD**K**L**D**LHYEL**T**R**N**R**-R-G**FV**L**G**K**G**I**A**T**VNDQ**PI**VT**I**E**K**L**L**I**YQA

Q88WG2_1-136 **M**E**VTDL**-----**IP**Q**RFPL**Q**LLDRI**VA**V**Q---PGVSAT**AEK**L**VTINE**W**FF**QSQTLTGRT**M**IRP**VLLE**I**LAQTGVVA**---L**L**SMPEH------HGNN**VFFGGIRQA**D**FKT**D**V**R**PGD**R**L**EAM**V**T**L**T**K**L**R**-R-Q**IGTG**H**G**VITC**AGR**E**VVSAD**-**L**T**F**VMQ

Q7UEY6_20-160 A**DIE**A**IR**--**EY**N**P**Q**RH**E**M**E**QL**TA**ILH**EDL--DQHACA**AYKAIT**E**NE**F**W**VR**GH**M**P**GM**PLMPGVVMLEA**V**AQ**L**S**SY**Y**---TQKHDLL------GAAM**VGFGGVDEVRFR**GV**V**T**PGD**N**L**IVL**VKL**E**K**A**R**RG-R**MI**V**A**RFQ**G**V**VGTE**L**V**L**EGC**-**L**R**G**IPI

Q820X6_11-156 **YSATEL**V--**EAIP**QKP**PF**R**FIDKILSV**----DENC**I**E**GI**Y**T**FKP**TE**F**F**YQ**GHFP**S**NPL**T**PGVILLESMAQVGLVA**(8)E**M**ARNEV------MN-L**VTLFT**EA**NV**E**F**L**S**PIK**P**T**D**T**I**T**IHAE**KT**L**W**R**RK-K**L**K**SH**AKAHKA**DGT**L**VG**I**AE**-**L**G**G**IGV

Q98MC5_10-149 V**DI**MG**LM**--**KLLPHRYPFLMIDRIIDI**D---GDDSAI**GIKNVTINE**P**HF**Q**GHFP**E**QPVMPGVLIVEAMAQTAG**A**I**---C**I**RSL----GASKPS-L**VYFLTIDNAKFRK**P**V**V**PGD**Q**L**K**IHVK**KI**K**K**R**-G-N**LLKF**A**C**E**A**L**VDGT**K**AAEAE**-**I**S**A**MMV

Q9ZED4_3-142 **IAITEIM**--**DLIPHRYPFLLVDRVLKI**D---PNKS**I**I**GIKNVTVNE**PQ**F**T**GHFP**A**RPVMPGVLMVESMAQ**L**AAIL**---V**A**KSL----DSTKNK-E**VFLMSIENTKFRR**I**V**Q**PGD**TMH**IH**SV**I**D**Q**Q**R**-A-N**VWKF**S**S**K**V**M**V**E**CE**I**AAESK**-**F**T**A**MIK

Q9A714_13-153 **IDI**A**EIL**--AR**IPHRYPFLLVDR**A**EDY**N---PHQS**I**V**GIKCVTINE**P**FF**Q**GHFP**G**NPVMPGVLIIEALAQTGAVL**---MSKSLEV---DTEGK-T**IFFMSVDNARFRN**P**V**R**PGD**V**I**R**MEVEV**L**R**A**R**-S-S**IFKF**K**G**V**A**K**VGDK**V**AAEAE**-**F**A**A**MVV

Q8R690_2-139 **LDI**L**EIM**--**K**R**IPHRYPFLLVDRILEM**DK--EAQI**I**K**GKKNVTMNE**E**FF**N**GHFP**G**HPIMPGVLIIEGMAQC**L**GVM**---V**M**E-------NFPGK-VP**YFAAIENAKFKN**P**V**K**PGD**T**L**I**YDVKV**D**K**V**K**-R-N**FVKA**T**G**K**T**Y**VDD**AV**VAEAS**-**F**T**F**VIA

Q97DA9_3-141 **LSIEQIM**--**EIIPHRYPMLLVDRVEEI**E---PGKRAV**GYKNVTFNE**Q**IF**Q**GH**Y**P**G**KPIMPGVLMIEALAQ**L**GGVA**---I**L**SLD-----KYKGK-KP**ILGAVKNAKFRR**M**V**V**PGD**V**L**K**LEIEI**V**K**V**K**-G-P**AG**I**G**K**G**I**A**T**VNGE**K**AVEAE**-**I**T**F**MIV

Q9CI03_7-144 **MTATEVM**--**EVIP**N**RYPI**M**FID**Y**V**D**EI**----SENK**I**V**A**T**KNVTINE**E**VF**N**GHFP**G**NP**T**FPGVLILESLAQAG**S**IL**---I**L**KKE-----EFQGK-M**AYIGGIDKAKFRQ**K**V**T**PGD**VMK**LEFEI**T**K**F**R**-G-K**VGTA**D**A**A**A**Y**VDGK**K**VTTCQ**-**F**T**F**IVD

Q8DSN8_2-139 **IDISKIR**--**EALPHRYPILLVDRVLEV**----SDDE**I**V**AIKNVTINE**P**FF**N**GHFP**QY**PVMPGVLI**M**EALAQTAGVL**---E**L**SKK-----ENTGK-L**VFYAG**M**DKVKFKK**Q**V**V**PGD**Q**L**V**MTAKF**V**K**R**R**-G-T**IA**V**V**E**A**K**A**E**VDGK**L**AASGT**-**L**T**F**AIG

O84537_7-148 **LGIQDIQ**--**NLLPHRYPFLLVDKILSY**DL--NTRS**V**V**AQKNVTINE**P**FF**A**GHFP**GA**PIMPGVLILEALAQAAGVL**---L**G**IILEN---DRDKK-I**ALFLGIQKAKFRQ**P**V**K**PGD**V**L**T**LKAEF**S**L**IS-A-K**GGKA**F**A**Q**A**F**VGSQ**V**VAEGE**-**L**S**F**VLV

Q9RVF5_7-143 **L**L**IRDVL**--**KALPHRYPFVLVDRVFST**----ENGE**V**H**ALKNVTINE**P**FF**M**GHFP**T**EPVMPGVLI**T**EALAQASM**F**C**---L**H**GQMEP------GQ-I**GYLAGIE**G**ARFKR**K**V**I**PGD**Q**L**H**LHAKL**E**F**L**R**-R-G**LGKT**T**C**R**A**E**VDGE**V**AAE**M**Q**-**I**L**F**AVA

Q8YUR4_31-170 **FT**S**EEIQ**--**KLLPHRYPFLLVDKIIDY**T---PGKQAV**GIKNVTINE**P**HF**T**GHFP**D**RPLMPGVLIVEAMAQVGGIV**---M**T**QLP-----GLEGG-L**FVFAGIDKVRFRR**Q**V**V**PGD**Q**L**V**MTVEL**L**W**I**K**QR-R**FGKM**Q**A**R**A**E**VDGQ**L**AAEGE**-**L**M**F**SLI

Q7MXT8_319-458 **MDINRIK**--**ELLPHRYPFLLVDKIIEV**----GPDY**I**V**G**V**KSVSGNE**P**FF**P**GHFP**G**EPVMPGVLQVEAMAQVGGLL**---V**L**NTL-----TEPSSYS**TYFL**M**IDKVKFRR**K**V**V**PGD**T**L**V**FKLRM**ISEIRR-G**VANM**R**G**L**A**F**VG**E**Q**L**ACEAE**-**F**M**A**QII

Q7RP73_87-230 **IDIDQIK**--**NILPHRYPFLLVDKVL**Y**I**Q---PNKK**I**I**GIKNVTANE**H**FF**N**GHFP**Q**KPIMPGVLQIEALAQ**L**GGIL**---C**L**KNSEN---KSKDN-L**FLFAGVD**G**VK**W**KK**P**V**L**PGD**T**L**V**MEVE**QI**L**F**K**PTLG**IAKL**K**G**V**G**Y**VGNH**V**V**I**E**I**E**N**M**I**F**AMS

Q7U319_13-164 **MNVEKIR**--**QILPHRYPMLLVDRVMEL**V(7)PNGY**I**K**AYKNVTINE**E**VF**L**GHFP**N**KPIYPGV**M**QIEGMAQAGGLL**---A**F**VSMFG(4)EAKNK-I**VYFMTIDNVKFR**IP**V**V**PGD**R**L**V**YELKV**L**K**H**K**-G-S**IWQL**G**A**N**A**F**V**E**DK**L**VSEAE**-**L**K**A**MIT

Q9WZQ8_1-135 **MNID**Y**VK**--**SILPHRYPFLLVD**G**VIE**E----SEDR**I**V**AFKNISIS**DP**VF**Q**GHFP**EY**PIYPGVLIVEGLAQTAGIL**---L**L**KSV--------EG-IP**LFLGIDEARFKK**E**V**R**PGD**R**L**I**YEVR**KLGE**K**-L-G**TVQV**E**G**V**A**K**VDDK**I**VAKAR**-**L**L**L**GVK

Consensus/80% bshpplb..phlPHRaPblblD+lbph........l.tbKsl*hsE.hF.GHFP.pPlbPGVlblEtbAQsthlh....h...............hhbhslpps+F+p.V.PGD.l.bphch.b.c....hhph.s.s.Vssp.sspsp.b.h...

**MaoC dehydratase-like subfamily (122)**

Secondary St. -------E—-EEE-----EEEHHHHHHHHHH-----EEEE-HHHH----------------HHHHHHHHHHHHHH--------------EEEEE—EEE-------------EEEEEEEEEEE---------EEEEEEEEEEEEE---------EEEEEEEEEEE------

Q8KRE2_7-143 TP**YE**A**L**E**VGQ**K**A**EY--K**KSV**E**E**R**D**IQL**FA**A**MSGD**H**NP**V**HLD**AE**FA**A-**K**---**S**M--**F**RER**IAHGMFSGALI**SA**A**V**A**CTLP-------GP**GT**I**YL**G**QQMSF**QK**PVK**I**G**----**DTL**T**V**R**LEILE**KLP**K**F-----------K**VRI**A**T**NVY---**N**QNDE**L**V**V**AGE**AEI**LAPRKQQ

Q7VUX2_10-154 **I**Y**WEDL**E**LGT**R**F**GT-RK**RTVTE**T**D**LVN**FV**N**LTW**LT**E**E**L**FSNGEP**A**D-**R**---**A**HMGITGR**VV**P**GGLVY**VF**A**EG**L**V**A**PSFQ-------AA**G**IA**FL**NA**ELD**IKG**P**T**F**V**G**----**DTL**H**V**E**CEVVQ**R**R**M**T**-**S**RPGRG---**LV**R**TR**NT**V**V---**NQHG**EP**VL**A**Y**TPLR**MM**RTRQI--

Q9A2B0_8-152 NY**FEDF**R**LGQ**R**L**VHATP**RTVT**AG**D**VALY**T**A**L**Y**GP**RFS**L**FSSDA**FA**Q-G---**C**G--LMSAP**VD**P**LIAF**H**VV**FGKT**V**PDIS------LNA**VA**N**LG**YA**EGRF**LA**PVF**P**G**----**DTL**S**A**VS**DVI**GL**K**E**N**S**N**RKTG----**VV**Y**VRT**T**G**V---**NQRG**EP**VLSY**VRW**VMV**RKRDA--

Q8F2Y4_30-174 **R**Y**LEEF**TE**GE**I**F**EHPRE**ITI**D**R**AFAQE**FA**T**T**F**MD**A**NPL**F**L**SAP**YA**K-A---HG--**F**QDM**LVS**S**LQVF**N**IA**LS**L**G**V**QNDS------EKA**LA**N**LG**YY**NVQF**LK**PVY**P**G**----**DTL**S**A**K**TKILK**V**D**D**K**G**S**DKPG----**IV**S**VRT**I**C**L---**NQN**KEL**VLQY**ERK**IMI**YRSNG--

Q82LU6_5-151 **R**T**YEEF**E**VG**AV**Y**KHWPG**KTVTE**Y**D**DHL**FC**L**LTMN**H**HPLHMD**TN**YA**E-**K**---**T**TD-**F**GKN**VV**V**G**N**Y**I**YSLL**LG**M**S**V**PDVS------GKA**IA**N**L**EV**ESLKH**VA**P**T**F**H**G**----**DTI**Y**G**E**TTVLD**K**T**P**S**R**S**KSDRG---**IV**Y**VET**K**G**Y---K**QDG**TL**VC**V**F**RRK**VMV**PTETY--

Q9HN18_32-177 **R**F**YEDF**A**VGD**V**Y**KHPYG**RTVTE**T**D**NVW**FT**N**LTMN**A**NPMHF**NAA**YA**S-G---**T**E--**F**DER**LVNGLFVIAL**SVG**M**S**V**VDVS------ANA**TA**N**LG**Y**DDIQH**HA**PVF**H**G**----**DTL**F**A**ES**EVLS**K**R**E**S**E**S**RPHVG---**MV**T**TEL**R**T**Y---**TQDG**EL**VLSL**ERTP**MV**RKRGA--

Q7WC65_7-152 **R**L**LEDF**K**TGD**Q**Y**AHWPG**RTITE**A**D**NIQ**FS**L**LTMN**R**HP**A**HCD**HH**YA**S-**Q**---**T**E--**F**GKP**LVN**S**GLTLAIV**LG**M**S**V**DDIS------ANA**VA**N**LG**W**QEIE**LLA**PVH**P**G**----**DTV**Y**A**RS**QVLS**V**R**E**S**K**S**RPGQG---**VV**T**VRT**E**G**V---RA**DG**VVF**MRF**VRS**CLV**PSRAS--

Q83EF0_19-164 **L**S**YEDF**T**VGD**I**Y**EHRPG**RTLT**LT**D**NIWQ**S**L**INMN**P**HPLHID**EE**Y**GK-**Q**---**T**E--**F**GQT**L**I**S**S**A**V**TF**C**VI**NG**L**T**V**NTLS------AKA**VA**N**LG**W**DKVR**LIN**PVF**V**G**----**DTL**Y**A**ES**KILS**K**R**L**S**K**K**RPHQG---**IV**V**VET**V**G**Y---K**QDG**SQ**VI**I**F**ERT**ILI**PRKGH--

Q7NBA2_1-139 **M**Y**Y**G**DF**N**VGD**V**F**EHRPG**RTV**L**D**V**D**NVW**FT**L**LTLN**P**Q**QV**HFD**QH**YA**D-**K**---**T**E--**W**KKL**LVD**S**TFTLALV**TE**M**S**V**NSIS------GKV**VA**N**LG**W**DKVR**LTN**PVF**A**G**----**D**M**I**Y**A**ES**TILS**K**R**E**S**K**S**RPIQG---**IV**T**V**L**T**REL----------**IKT**IKK**LLA**SKELY--

Q99Q03_21-166 **L**A**Y**G**EM**TE**G**LV**I**EHRPG**RTITD**T**D**NVLM**T**S**LGGN**DA**PLHTD**HH**Y**SR-**H**---**T**A--**W**GRP**LVC**SS**I**I**L**H**IV**GG**M**T**V**RSTS------GLT**IA**N**LG**F**DQIR**CTH**PVF**V**G**----**DTL**Y**A**E**TEILE**R**R**R**S**R**S**RPTAG---**LV**T**CRT**T**G**H---**NQD**HVT**VMTF**TRT**FLV**PLDTD--

Q98LS1_4-150 **L**Y**LEEF**V**VGH**V**F**QHTLR**KTVTE**S**D**NML**FS**V**MTLN**P**QPLHID**FD**FA**A-**R**---**S**E--**W**GKP**LVN**S**LFTLGLM**IG**I**S**V**NDIT-----VGTT**VA**N**LG**M**KETTF**PH**PVF**H**G**----**DTI**R**V**E**TTVIS**V**R**E**S**K**S**KPDRG---**IV**E**FEH**R**A**Y---**NQQG**DL**VAKC**TRQ**AMM**LKKAA--

Q7TYF8_33-181 **L**W**FEEF**Q**IGT**T**Y**LHRPG**RTVTE**A**D**NVL**FT**T**LTMN**T**Q**S**LHLD**AA**WA**G-**Q**---QPGFRGER**LVN**S**MFTLS**T**M**VG**L**S**V**AQLT-----LGTI**VA**N**LG**F**SEVSF**PK**PVF**H**G**----**DTL**Y**A**E**T**VC**T**GK**R**E**S**K**S**RPGEG---**IV**T**LEH**I**A**R---**NQHG**EV**VARA**VRT**TLV**QKQSI--

Q7W4Z4_4-150 **L**Y**FEDF**K**AG**MV**V**QHAIR**RTVTE**T**D**NVL**FS**A**LTYN**CA**PLHID**AE**Y**SA-**D**---**T**I--**Y**GQR**LVN**S**MF**L**LALV**AG**V**T**V**YETT-----LGTT**LG**N**LG**FG**EI**V**F**PK**P**T**F**HV----**DTI**R**V**E**TEILQ**T**R**L**S**R**S**RTDSG---**IV**T**FKH**V**A**R---**NQRD**EI**VCTA**VRT**GLM**MLRPA--

Q9HRM9_4-152 **R**Y**YE**G**F**E**VGE**T**I**EHATR**RTVSE**A**D**NQE**FC**D**MTMN**Q**QPLHLD**AA**FA**S-**E**---**T**Q--**F**GER**IVNGLYTMSLA**VG**I**TIPETT-----DGTI**VA**N**L**SY**DDVEH**PA**PVF**H**G**----**DTI**R**V**QS**TVTD**K**R**E**T**S**D**GERG----**VV**T**MRV**E**V**FAVNR**ED**EPL**VCEF**ERT**VL**SLKRAH--

Q976X1_8-157 PY**FEDF**K**VGQ**R**F**KSKIG**RTITD**V**D**NIW**FT**L**LT**N**N**S**N**Q**IHF**NKD**Y**TE-**K**YFP**G**EPFKGRL**VVNGFLTLAIV**AG**M**L**V**EQTS-------QN**G**FM**LG**L**ENVKF**LN**PVF**A**G**----**DTI**Y**A**E**AEVIE**V**R**E**S**K**S**RPGFG---**IV**K**IRT**W**G**Y---**NQRG**EK**VVEF**DRV**FMV**RKKGV--

Q93TU5_19-164 **R**Y**FEDF**P**VGT**V**C**VT-GG**RTV**DMS**D**ISM**FA**G**LTGD**HY**PLHTD**EQ**YA**S-**R**---**T**Q--**F**GTR**IAHG**P**FTF**C**IA**VG**L**V**A**LAGF----YGEAI**VA**MREV**QSIR**ALK**PVR**P**G**----**DTV**H**V**R**VEVV**AA**E**PAR**K**PDQG----**IL**S**LDY**T**V**V---**NQHD**ET**VMTV**RMV**LLA**KRRSP--

Q976V4_1-144 **M**Y**FEDF**Q**VGQ**K**W**ES-KG**RTVTE**A**D**VVL**FT**G**LTGA**L**NPL**F**LD**EE**YA**K-**T**---**T**R--**F**KGR**I**LP**GLLTASIG**VG**L**TYQLPS---DPFGEG**FV**A**LT**KL**EIN**AKK**PVK**I**G**----**DTL**K**A**L**VEVVN**K**Q**E**R**E**K**DG------K**V**Y**LKI**S**V**I---**NQN**KEE**VM**I**L**NME**ILA**NKRS---

Q89Q33_9-154 **L**R**YEDI**A**LG**AE**F**ET-AA**HTVTE**A**D**IAV**FA**D**VT**R**D**H**HPLHLD**AG**YA**K-**S**---RG--**F**PAV**IGHGLFGLSLM**EG**L**K**S**ELKL----YEETS**VA**S**LG**W**DEVKF**KG**P**IVA**G**----**DSL**R**V**R**FR**F**VE**K**R**P**T**K**N**PARG----**IV**IE**TL**D**L**L---**NQRD**EV**VTTA**RHTS**LI**LTRQA--

Q89GF1_5-148 **R**Y**WDD**AV**VGD**E**C**IT-PSV**TVTE**AMVNGY**A**E**LTGD**F**TP**V**HVD**EE**YA**R-**T**---**T**P--**F**GTR**VAHGLFGLSLA**DG**L**K**T**RAEY------------------**RF**LPGMSL**G**(12)**DTV**H**V**K**FRVGS**M**R**T**T**K**R**EGWG----**IV**V**L**PSE**L**I---**NQHG**QV**V**QL**G**EHR**LMI**PMRPK--

Q89DK1_16-161 **L**T**FEDF**PP**GR**F**G**TF-GP**R**H**VTR**D**E**ILA**FA**A-E**FD**P**QPMHLD**ED**AA**S-**K**---**S**M--LRGLS**GSGWH**L**CSLM**MR**M**M**A**DGFI----TRAAS**LG**SP**G**V**DEVRW**LS**P**L**R**P**G**----**D**D**L**MLD**VDVME**A**R**A**S**K**S**RPGLG---**IV**K**FKC**T**V**R---**N**AA**G**QV**LCEM**TSP**ILI**ERREG--

Q7VY27_1-146 **M**K**F**A**DL**R**AG**MV**I**TG-GP**L**Q**VTE**A**E**ILE**FA**R-K**FD**P**Q**W**FHTD**VQR**A**A-**E**---**G**R--**W**GGL**IASGWHTCALA**MR**M**A**V**DAIL----HDSES**FG**SP**G**LG**EVSW**RV**PVR**P**G**----**DTL**TLH**ARV**QGA**R**V**S**A**S**RNDLG---**IV**N**W**A**W**V**V**D---**NQHG**ET**VLEL**DATS**LF**DLSGD--

Q8XXS0_10-155 **F**Y**FDDF**Q**VGQ**T**M**EM-GT**Y**A**VTE**D**E**ILA**FA**R-Q**YD**P**QPFHVD**PE**AA**R-**R**---**S**I--**Y**GGL**ISSGWMTCAVM**MR**L**M**V**QNFL----SKSSS**MG**SP**G**V**DEIRW**LR**PVY**P**G**----**DTL**S**V**SS**T**C**LE**V**R**P**S**Q**S**KPDRG---**V**AIN**RW**E**A**R---**NQHG**EL**VCTL**VGM**GLF**GRRPA--

Q8UD93_19-164 **Y**T**YEDF**A**VGR**E**F**PL-GP**QSIS**AAQIIE**FA**S-E**FD**P**QPMHL**SEE**A**GR-**R**---**S**I--LGGLA**ASGWHTCSLL**MR**M**M**A**DSYI----SNSTSQ**G**SP**G**I**D**Y**VDW**KK**PVL**A**G**----**DTL**S**G**KSI**VLE**Q**R**P**S**A**S**RPGIG---**LV**K**LRH**E**L**Y---**NQRG**IL**V**S**QG**ENT**VMF**LMGGD--

Q92N63_3-156 TL**EE**L**Y**Q**AGR**K**V**VT-GS**LT**F**T**AE**D**IVR**FA**R-**DFD**P**QPFHVD**EEQ**A**R-**Q**---**S**L--**F**GGLC**ASGWHT**S**A**G**W**MQ**C**FLRFWK(11)HAPKL**GP**SP**G**F**KELRW**LK**PVY**A**G**----**DTI**TYA**VTLLE**A**R**IVA**S**RPGWR---**I**NT**I**L**C**E**G**E---**NQHG**EP**VIRF**ESK**VI**EFA-----

Q8YIG7_1-157 **M**S**F**I**EE**N**IGK**ERVL-GT**YT**F**T**AE**E**IIA**FA**R-K**YD**P**QPFHID**EE**AA**K-**N**---**S**L--**F**GGLC**ASGWHTTAIF**MK**L**N**V**ASMV(11)TPPTF**GP**SP**G**F**ENLKW**PK**PVF**A**G**----**DTI**TYKRV**VH**AI**R**PLA**S**RPGWS---**ML**T**MTT**R**A**H---**NQHG**EE**VLSF**DNA**AMV**KLPPK--

Q89NH7_5-148 **E**W**FDDL**S**IG**MR**F**KS-PEVE**VTE**A**D**IKR**FA**A-E**FD**P**QPMHLD**HE**AA**K-G---**T**L--**F**NGLA**ASGWHTAAIA**MN**L**AIQTRP----FGPHP**L**IG**AG**V**D**G**LRW**TI**PVR**PN----**D**R**L**HLV**GEVMS**L**T**P**S**K**S**KPQG----**I**AL**VKW**T**M**F---**NQNG**EE**VYTF**TPI**AIV**PRRA---

Q7NTY2_2-148 **L**Y**FEDL**RP**GQ**L**F**LS-AE**HTLSE**Q**E**IIA**FA**R-Q**FD**P**QPFHTD**PE**AA**R-**Q**---**T**F--**F**NGLA**ASGWHT**S**SL**SMR**L**V**A**ESEL---GRAANG**L**IG**M**QI**DKMRW**PQ**P**S**R**P**G**----**DTL**Q**V**E**VEVLD**K**R**R**S**S**S**QPGFG---**VV**K**VSW**T**T**R---**NQRG**ETA**MQL**ECA**IWL**QCRSK--

Q988L8_4-147 **L**Y**LDDL**ER**GQ**T**F**VS-AS**H**A**L**D**E**QQIKA**FA**R-E**FD**P**Q**I**FHL**ERA**A**EG-----**T**L--**F**GGLA**ASGWHTAAIT**MR**L**N**V**ESGL-----PLAN**G**II**GA**GG**EINW**PN**P**T**R**P**G**----**D**I**L**H**V**ES**EVMD**IAP**S**R**S**RPERG---**IV**T**I**VSK**T**L---**NQRG**DV**L**QI**L**TAK**L**V**V**FRRPA--

Q8XQU8_14-159 **R**Y**LEDF**K**VGD**L**T**ET-SG**F**A**VTR**DMILS**FA**E-Q**YD**P**QPMHLD**ET**AA**R-**D**---**T**V--**F**GEL**VGSGWQTLAVT**MR**L**LLDARL----LGGTP**IV**G**A**EL**RDIRF**HA**P**M**R**P**G**----**D**V**L**R**A**R**AEVI**AI**R**P**S**K**S**RPDRG---**F**MD**ARV**T**T**T---**NSEG**VT**LVT**QRWS**L**V**V**PRRPD--

Q9LBY7_1-142 **M**K**YDDF**I**VGE**T**F**KT-KS**L**H**ITE**E**E**IIQ**FA**T-**TFD**P**Q**Y**MHID**KEK**A**E-**Q**---**S**R--**F**KGI**IASGMHTLSI**SFK**L**W**V**EEGK----YGEEV**VA**G**T**QM**NNVKF**IK**PVY**P**G**----N**TL**Y**V**I**AEITN**K**K**SIK**K**ENG-----**LV**T**VSL**S**T**Y---**NEN**EEI**VFKG**EVT**ALI**HNS----

Q7WE75_17-160 **R**Y**FEDF**E**VGE**R**F**VL-PS**RT**M**TD**ALFAA**F**QL**ASGD**N**HPIHYD**VE**Y**CR-A---YG--MPHM**LAHGFQVV**IQ**T**AA**G**A**G**LFPH----MTEAS**M**KG**FI**E**QS**S**RF**VG**PVF**V**G**----**DTL**Y**C**S**LEIVE**L**K**PGR**T**TG------**TV**R**MKT**L**V**V---**NQKG**ET**VMEG**SQT**YLL**KKRRP--

Q9LBK2_7-143 **I**P**Y**A**EL**E**VGQ**K**A**EY--TS**SI**A**E**R**D**LQL**FA**A**VSGD**R**NP**V**HLD**AA**YA**A-**T**---**T**Q--**F**KER**IAHGMLSGALI**SA**A**I**A**TVLP-------GP**GT**I**YL**G**QTLRF**TR**PVK**L**G**----**D**D**L**K**V**E**LEVLE**KLP**K**N---------R**V**R**M**A**T**R**V**F---**NQ**A**G**KQ**VVDG**EAE**IMA**PEEKL--

Q9A2Q8_10-146 **Y**I**LEEL**S**VG**MT**A**EK--HV**TVTE**ERIQR**FA**E**AS**D**D**F**NP**V**HVD**EA**FA**A-**K**---**T**A--**Y**RGR**IAHGLLSAS**F**G**SA**V**V**G**TILP-------GA**GA**I**YL**G**QTLTF**HK**PVR**I**G**----**D**V**V**T**A**R**ATVAS**I**D**T**E**SA--------R**V**V**LRC**A**A**L----VG**D**EV**VMDG**EAT**VRV**PRRRR--

Q7NRG2_3-138 VY**FEDL**A**VG**AS**A**ES--G**KTITE**A**D**VLL**FS**A**VSGD**N**NP**V**HLD**QT**YA**A-**T**---**T**P--**F**ETR**IAHGMLTASLI**SG**V**I**G**TRLP-------GY**GT**V**YL**S**QSTRF**KA**PVR**I**G**----E**TV**T**T**R**VTV**E**E**LIP**E**K**K**--------R**V**R**LST**Q**C**R----VG**D**KV**VLEG**ESL**VIA**PSRN---

Q81G96_13-147 **L**R**YDEI**Q**VGD**Q**A**SL--T**KTITD**E**D**VIN**FA**K**LTGD**V**NPIHI**LDS**FA**K-**T**---**T**M--**F**KER**IAHGMLV**S**S**F**I**ST**I**L**G**TKLP-------GKN**T**I**YL**S**QNVSF**RA**PVK**I**G**----**DTL**R**V**V**AEVIK**K**R**D**D**K**K**--------**II**T**LQT**N**I**Y---**NQSD**DI**VVEG**TAT**IL**KKE-----

Q81RV8_2-133 ---**T**M**Y**S**TGQ**Q**A**SC--S**KTITE**T**D**FVL**FA**G**LSGD**F**NPIHID**HE**YA**K-**Q**---**T**R--**F**NQR**IAHGLLT**S**SLL**SQ**L**L**G**IHLP-------GK**GS**V**YM**E**QTIKF**TA**PVF**I**G**----**DTI**T**A**T**ATV**Q**E**FMM**E**K**R**--------**VL**K**L**L**T**E**C**H---**NQKG**DL**VLTG**VAT**MMV**PK-----

Q8EYU8_6-143 **K**S**YEEI**E**IGE**K**A**SF--T**KTITE**T**D**IYL**FA**G**ISGD**F**NPLHVD**EE**YA**K-**T**---**T**I--**F**GTR**IAHGGLAASLL**AP**V**L**G**MKLP-------GL**GT**V**AL**E**T**V**TKF**RK**PVY**P**G**----**DTV**T**C**I**VEV**K**S**KIE**K**M**K**--------**MI**Q**MKI**L**W**T---**NQKG**EM**IGKG**ECK**VL**PPGFNI--

O32472_2-134 -SA**Q**S**L**E**VGQ**K**A**RL--S**K**RFGAA**E**VAA**FA**A**LS**E**D**F**NPLHLD**PA**FA**A-**T**---**T**A--**F**ERP**IVHGML**L**ASLF**SG**L**L**G**QQLP-------GK**GS**I**YL**G**QSLSF**KL**PVF**V**G**----**D**E**V**T**A**E**VEVT**AL**R**E**D**KP--------**I**AT**LTT**R**I**F---**TQ**G**G**ALA**VTG**EAV**VKL**P------

Q7X2V0_85-222 GTTMR**I**K**VGD**S**A**FL--T**KTITE**A**D**IQT**FG**D**LSGD**H**NPLHFD**KD**HA**Q-**R**---**T**R--**F**GRP**IGHGMLTGSLF**SP**I**I**A**HQLP-------GE**GA**I**YL**S**QSLRF**VA**PVF**A**G**----**DTI**T**A**E**LTVTH**V**R**E**D**K**Q**--------**IV**T**L**A**G**V**A**K---**NQRG**EV**VITG**ESV**VLV**EALNQ--

Q9FJI2_21-159 AS**KT**L**L**K**VGD**V**L**RE--T**R**VF**S**SE**D**IKAY**A**E**VSHD**W**NPLHFD**PES**A**R-**K**---**A**G--**F**ENR**LVHGMLV**S**S**M**F**PR**I**I**S**AHFP---------**GA**V**YV**S**QSLHF**RS**PVY**I**G**----**D**E**I**L**G**L**VQ**A**I**AL**R**E**T**K**N**KY------**IV**K**FST**K**C**FK--**NHN**ELV**VIDG**EAT**AIL**PNLDM--

O29141_31-159 **K**F**E**G**EL**KE**G**YR**F**EY--E**K**K**L**C**E**I**D**VAM**FG**L**ISGD**L**NP**V**HFD**ED**FA**S-**K**---**T**R--**F**GGR**VVHGMLTTSLV**SA**A**V**A**RLPG---------**TV**V**LL**E**QSFRY**TS**PVR**I**G**----**D**V**V**R**V**E**G**V**V**SGV**E**K**N**RY--------**TI**D**VKC**------Y**T**G**D**KV**VAEG**VVK**VLI**W------

Q833N7_11-148 **K**T**IEDI**EE**GD**S**L**SL--T**ESI**E**D**K**D**LLLYLG**LT**N**D**A**NPL**Y**I**QHD**YA**Q-**K**---**T**E--**Y**EKP**IV**PS**IM**L**MGII**TS**A**I**S**KHLP-------GP**GS**H**VV**NF**SVNF**VE**PVF**HY----E**TL**TFQ**LEVIK**V**D**KMK**D**--------**VI**T**ISV**E**A**V---**NEQ**ENR**VLDA**VVM**VQ**PPQVTI--

Q89Q24_5-142 **K**S**FDDL**K**V**N**D**R**V**SL--S**KTITE**A**D**GALYIA**ATGD**FG**P**V**HVD**EV**YA**G-A---**T**R--**F**GRR**LA**P**GIMVAGLC**TS**I**L**T**SELV-------GT**IG**VS**V**E**DRF**W**F**TG**PVF**Y**G**----**DTL**TFD**V**W**IAE**Q**H**D**E**T**R**--------**TI**I**WEA**S**A**R---**NE**G**G**LE**VLKA**RAS**LKF**PRRKP--

Q7WN88_13-158 **L**YGV**DL**RP**G**LASHG-SPA**TITR**T**E**NLG**FA**A**LTGD**S**HPIHYD**EA**FA**A-**R**---**T**R--**H**GRC**VVHGLL**L**ASLG**AF**G**A**T**PLSR----RIEDA**MV**A**FV**D**SQFSF**LK**PVF**I**G**----**DTV**T**S**H**FEVAQ**V**E**H**K**PARNLS----**LL**R**FDM**W**L**T---**ND**A**G**DT**VMQG**KHT**YLV**RMQPD--

Q7VX71_7-156 **L**AAA**DL**A**VGQ**V**V**AQ-RE**I**R**L**DAQAFAQ**FA**A**LTGD**A**HPIHYD**AG**YA**Q-**R**---QG--LRAP**IAHGLL**L**VAI**SAL**G**A**T**PLSA----QLHDA**MV**A**MV**DV**EA**V**F**MR**PVY**VD----E**TV**Q**C**V**YRVAQ**I**E**HVA**K**GRSKT---**TI**E**VSI**H**T**TDAP**PD**A**A**RQA**HCV**VHL**TFL**LKTHL--

Q7UGS9_39-185 **L**YC**EDL**Q**VGN**E**W**LS-PW**RTIT**AD**D**VRA**FS**V**LTGD**F**DPLH**DEDENGESLMPK**S**P--**F**GRP**VAHGLLGLSVL**AG**L**S**T**EYPR------AAT**LA**L**V**SV**SDWNF**DN**PVF**F**G**----ER**V**R**V**V**TTV**E**S**T**E**A**H**G**R**RAA-----K**I**T**WH**RQ**L**I---**SDDG**RT**L**Q**QG**KFV**TLV**ASNKR--

Q7VVT2_3-145 VE**FE**R**I**Q**LGE**R**H**VS-SR**ITITE**SHIVT**FA**G**LTGD**F**NPLHMD**EV**AA**R-**E**---**N**G--**Y**GQR**IAHGMLGHAV**STG**L**R**S**GIDD-------WD**IV**A**FL**E**TR**R**RF**VA**P**I**L**A**G**----**DSV**RYE**AEVLE**L**R**P**S**R**S**KPFG----**VV**K**VSM**Q**L**R---**NQDG**VT**V**Q**DG**EDV**F**A**V**AMGGG--

Q7WWU5_5-150 **L**Y**FEDF**LR**GQ**V**F**ES-TG**RTITE**T**D**LTM**FS**M**LSGD**W**NPIHAD**AE**FA**R-G---**T**R--**F**GQR**VVHGALGIALA**TG**M**LHQLGV----FDKSA**VA**M**M**SL**QNWTF**AA**P**I**F**V**G**----Q**TL**RLR**MEILE**T**E**AGT**S**KRVG----R**V**NR**RL**Q**L**I---**DQTG**GV**I**Q**DG**TTD**VLV**LKRDA--

Q7W0R1_10-150 **Q**T**FDEI**E**VGK**V**F**RS-GG**RTITE**T**D**VVN**FC**A**LTGN**WIE**IH**SNVQ**YA**S-**K**---**T**R--**F**GKR**LVQG**S**LTYSIV**TG**L**IQFGLG--------IQ**A**N**YG**I**DNMRF**LA**PV**SI**G**----**DTI**Y**A**V**CEVMR**K**K**E**K**D**E**KYG-----**VI**T**F**F**M**K**A**V---**NQDG**TV**V**Q**KG**EWS**LLM**LRRRE--

O27971_4-149 **L**Y**FEDF**E**IG**MK**V**ES-AA**RTVTE**A**D**IVM**FA**S**LSGD**W**NPIHTD**AE**FA**K-**K**---**T**I--**F**GQR**VAHGLLTLSVV**AG**L**L**V**RLGL----TERTI**VA**F**YG**I**DKLRF**TN**PVF**I**G**----**DTI**R**A**V**LEVV**GK**E**D**K**EGKPYG----**VV**V**YDI**K**G**V---**NQRG**EV**VITY**TSR**A**A**I**LKRRP--

O28346_13-160 **I**Y**FE**S**I**Q**IGE**K**I**EG-LP**RTVTE**T**D**IWT**FA**Y**LTAD**FF**PLHTD**VE**FA**K-**K**---**T**I--**F**GKP**IAQGMLVLSIA**LG**M**V**D**QVIL-SNYDVSSV**IA**F**FG**I**KDVRF**LR**PVF**I**G**----**DTI**A**A**S**AEVVE**K**Q**DFD**E**KSG-----**VV**T**YKL**E**V**K---**NQRG**EL**VLTA**LYS**ALI**RKTPS--

P77455_529-677 **K**Y**FEEL**QP**GD**S**L**LT-PR**RT**M**TE**A**D**IVN**FA**C**LSGD**HFYA**HMD**KI**AA**A-**E**---**S**I--**F**GER**VVHGYFVLS**A**A**AG**L**F**V**DAGV------GPV**IA**N**YG**L**ESLRF**IE**PVK**P**G**----**DTI**Q**V**R**LT**CK**R**K**T**L**K**K**Q**RSAEE(4)**VV**E**W**A**V**E**V**F---**NQH**QTP**VA**L**Y**SIL**TLV**ARQHG--

Q92TG5_522-666 **K**S**L**A**EL**R**VGD**Q**I**VT-ET**RTVT**LE**D**IEH**FA**E**FTGD**TFYA**HMD**EE**AA**RA**N**---**P**F--**F**DGR**VAHGYLVVSLA**AG**L**F**V**DPAP------GPV**LA**N**YG**V**D**G**MRF**LT**PVY**P**G**----**DTL**Q**V**R**LT**CK**E**I**S**P**R**I**N**SDYG----E**V**R**WDC**R**V**T---**NQTG**AT**VAQY**DVL**TMV**AKTGS--

Q986Z7_5-148 TY**FEDY**E**IGS**SRLT-SG**RTITE**T**D**FVVH**A**G**HTGD**FF**PHHMD**AE**Y**MK-A---**T**P--**F**GQR**IAHGTLVFSVG**IG**L**T**A**SIIN-------PV**A**FS**YG**Y**DRLRF**IK**PVF**I**G**----**DTI**R**T**R**TTIT**AK**E**D**D**T**K**RPGSG---R**V**IE**RC**E**V**I---**NQRG**EV**VL**A**A**DHI**YIV**ERKPA--

Q92UM3_33-174 **W**F**YEDW**P**VGQ**R**I**RS-LR**RTI**G**E**S**D**SHL**FN**T**LVVD**I**HPY**VQ**D**QM**FA**E-----REGI**F**GRR**LV**A**GAFVFS**A**G**LG**L**V**A**TNCV--------N**A**FS**YG**Y**DKLRF**IK**PVF**I**G**----**DTI**Y**T**IR**T**N**ME**K**T**P**R**Y**K**DLG-----**LI**R**ASY**Q**V**F---K**SEG**EL**VL**Y**C**EHLQT**V**KYKNP--

Consensus/80% b.bp-b.hGp.h.....b*l*c.-...Fs.hshs.pPbHhD..hA..p...s...a...lspGhbshtlh..h.s.............hs.hh.pphpa..PVb.G....D*l.s.hplhp.p.p.p........hl.hph.h....spps..lhph...hbh.......

**YbgC-like subfamily (102)**

Secondary St. -------------EEE-HHHHHHHHHHHHHHHHHH----------------EEEEEEHHHHHH---------EEEEEEEEE----EEEEEEEEE----------EEEE—-EEEEEEE--------------

Q9HTM6_15-133 S**LRVRW**A**EVD**PQ**GIV**F**NGNYL**T**YL**DV**A**TT**EYY**RQL**GM**SYPADLLRGG----GD**L**FAV**K**S**TLEYR**A**PA**H**FDD**W**L**D**I**G**T**R**V**A**RL**GRS**SL**V**F**E**L**G**IWR**------DEQ**LLT**V**GE**LV**YVY**A**D**AN**E**RQ--SQP**L**PDW

Q8E9A9_10-128 D**MQV**P**F**H**DVD**S**MGI**TW**HGNYL**R**YFE**V**AR**C**KLL**DEL**GY**NYRQMRASNY----A-**W**P**I**I**DVQIKY**VK**P**ST**F**EQH**I**T**VRA**E**L**V**EW**EN-**RL**K**I**N**YQIRD**----TATGER**IT**K**G**Y**T**IQ**AAVD**MT**S**QE--MCF**V**TPE

Q87RA9_14-132 T**M**VTS**F**Q**DAD**P**MGVI**Y**HGN**F**F**R**YFE**E**AR**RV**LM**EKIQ**Y**GYRDMQDSGY----M-**W**P**I**I**DTRVKY**VK**AI**P**FNH**T**I**R**ITA**Q**L**T**EW**EN-**RL**RVD**Y**V**IYD**----AETNQR**MC**K**AHT**TQ**VAVS**IE**K**QE--MCF**V**SPA

Q9ZLX8_2-125 R**CRVYY**E**DTD**SE**GVV**Y**HANYL**K**Y**C**E**R**AR**S**EFF**----**F**KQNVLPENEE----GV**F**V**I**R**SIKADFF**T**PA**S**LG**QV**L**E**IRT**Q**I**K**EL**RKVF**V**V**L**F**QEIYC**(6)EPMKPFK**V**FA**SE**IK**FGFVN**RS**T**YS--PIA**I**PKL

Q7VHS7_5-128 H**VRVYF**E**DTD**C**G**R**IV**Y**H**T**NYI**K**Y**C**E**R**AR**S**ELF**FAQ**GA**QPFENQSA--------**F**V**L**K**KLEADFL**A**SA**R**LGD**M**L**E**VR**SA**I**A**QF**KNV**SV**V**L**K**QDIYR**(6)QKECEE**II**FK**A**YIT**LAFVD**VE**R**GK--PCK**I**PSS

Q9PNX0_2-115 K**MRVYY**E**DTD**A**GGVV**Y**HSNYL**K**F**C**E**R**AR**S**EIF**FNKK**V**DIFDASKGH-------**F**L**L**A**KANCNFL**K**PA**K**LGD**M**I**E**IKT**K**I**L**EV**KNA**SV**E**I**L**QEIYK**------DEI**LL**FKM**E**LT**LAFIK**NE**K**----PAR**M**DMQ

Q7MRK3_2-117 K**IRVYY**E**DTD**C**GGIV**Y**HANYL**K**F**C**E**R**AR**S**ELF**FAQ**GM**RPEEGGYS--------**F**V**V**R**NLQARFL**S**SA**R**LGD**E**I**W**VSA**TPKL**I**KSA**SL**T**L**I**QEIR**L---GDDSGK**IL**FAM**EV**E**VVCLK**GG**K**----VAK**I**PDF

Q8Z8C2_9-127 P**IRVYY**E**DTD**A**GGVV**Y**HASY**VA**FYE**R**AR**T**EML**RHH**HF**SQQVLLAERV----A-**F**V**V**R**KMTLEYY**A**PA**R**LDD**M**L**E**VQT**E**I**T**SM**RGTA**L**V**F**T**QRI**V**N**-----AENT**LLN**E**AEV**L**IVFVD**PL**K**MK--PRA**L**PKS

Q9WWX4_13-132 R**CRVYY**E**DTD**A**GGVV**YYV**NYL**K**FME**R**AR**T**ERL**RHL**GF**SQSQLAEDNL----L-**F**V**V**H**S**S**EARYH**A**PA**R**LDD**E**L**R**VTA**Q**V**L**EL**NRA**SL**R**F**V**QQVWR**----EKDET**LLC**E**GQ**FL**VAAVR**AD**T**FK--PRA**L**PPQ

Q8EQL3_7-125 P**IEVRY**Q**ETD**Q**MGVV**Y**HANYL**V**WFE**I**GR**T**KYI**EAI**GL**KYNDMEKHGV----V-SP**V**I**DANISFK**R**PI**R**YAE**ETH**VET**W**L**E**SY**DGI**RT**V**Y**G**YKII**-----NEQGK**VAV**E**GTT**T**HTIVN**KE**T**FR--PLS**V**RRT

Q81Y90_7-125 E**VEIRY**A**ETD**Q**MGVV**Y**HSNYL**V**WLE**L**GR**T**KLI**QDL**GF**SYVEMEKEGI----I-SP**V**L**DLQISYR**K**AM**R**YGE**KAI**VKT**W**V**D**TV**SPL**RV**V**Y**G**YEIY**-----NGDGE**LC**IT**AST**TNI**C**A**K**KEGFR--PVS**F**KKF

Q8R916_7-125 E**IRVRY**G**ETD**K**MGVV**YY**ANYL**H**WFE**I**GR**T**EFF**RSL**GM**TYRDLEERDI----M-**L**P**V**I**EAHCKYF**S**SA**F**YDD**L**I**I**IRT**R**L**EF**V**TGT**RI**K**F**L**YEVIR**----KEDGK**LLA**Q**G**Y**T**E**HPF**T**D**ST**R**K---PIN**L**KKV

O67466_6-121 RR**RVQF**Y**ETD**AQ**GIV**H**HSNYF**R**YFE**E**AR**G**EFL**RSK**GF**PYSKMRDMGL----E-**V**V**L**L**NA**Y**CEYK**K**PL**F**YDD**VFE**VHL**N**L**E**EL**SRF**TF**T**F**S**Y**I**VFK**------EDI**AVA**K**ANT**K**HCMVK**NG**K**----IVS**I**PKE

Q7UFY0_22-138 R**LRVRY**D**ECD**P**MGLV**H**HSNYL**R**YFE**I**GR**T**EFL**RSS**GG**RYREVEEAGL----Y-**V**V**V**V**HIDCRYR**A**SA**R**YDD**E**I**D**I**V**T**R**I**A**KI**TAA**KI**I**H**E**YEIRR**------GDE**VLV**Q**ATV**T**LAVID**KTGR---LQR**V**PEA

Q8DUU8_5-125 H**H**L**VQY**Y**ETD**R**MGI**TH**HSNYI**R**WME**E**AR**V**HFL**AEI**GW**PYDKLEEAGI----I-SP**V**TA**VHC**I**YL**A**T**ST**FAD**T**I**S**ISV**E**V**E**KV**KAA**RL**T**L**S**YQMIN**-----QKGK**TVC**Q**AQS**E**HSFLT**LE**N**RFVNLKKNFPD

Q97MC7_7-126 R**IKVRY**A**ETD**K**MGIV**Y**HANYY**V**YFE**A**AR**E**DLI**EGA**GI**KYSDMEDIGI----M-**M**P**L**V**ETKCKYH**E**GA**K**YGD**Y**I**L**VET**T**L**G**KL**SPI**KV**E**I**N**YRVLR**----ESDGK**LLA**E**GQT**TQ**VFVD**AQ**N**FK--IIK**L**MKS

Q7MJ83_11-129 P**VTVYY**E**DTD**A**GGVV**Y**HSNYL**K**FFE**R**AR**T**ELL**RSV**GI**SQNVLLEQNI----G-**F**V**V**R**HMDIDFI**Q**GA**R**LD**QH**L**T**I**L**T**K**I**G**EI**KRA**SL**L**F**C**QEL**V**N**-----DDGK**LLC**K**ATV**K**VACID**NV**K**MK--PIA**I**PTS

Q7W480_12-130 D**IRVYY**E**DTD**A**GGVV**FY**ANYL**K**FLE**R**AR**T**EWL**RGL**GV**NQSDLAEREH----RL**F**V**V**H**SLDMSYR**K**PA**R**LDD**L**I**T**IR**SR**I**T**RI**GRA**SI**H**F**A**QRAER**------NAE**LLA**Q**GN**IQ**ICCVD**SI**R**MR--PAE**L**PDD

Q8P6E8_19-137 P**TRVYW**E**DTD**A**GGVV**Y**HA**R**Y**VA**FME**R**AR**T**EWM**RAL**GF**GQERLRQQHD----LV**F**A**V**R**SMQLDFL**K**PA**R**LDD**A**L**S**VSA**V**L**T**RC**KRA**SL**I**F**A**QSVRR**------EGE**VL**LT**AEV**R**IAAL**GGS**D**FR--PRG**M**DDA

Q82XP3_8-126 P**VRIYY**Q**DTD**A**GGVV**Y**HASYL**N**FLE**R**AR**Y**EWL**REL**GF**TVDTMIRSHK----MI**F**L**I**R**SL**G**IEYF**K**PA**V**LDD**L**L**D**ITV**Q**V**V**DI**GRS**RI**T**L**Q**QQILR**------EQG**TLA**S**ATV**H**AVCV**GAE**T**LK--PIS**I**PAP

Q92M94_18-138 IQ**RVYY**E**DTD**FS**GVV**Y**HA**R**YL**H**FME**R**AR**T**DYL**RLL**GV**EQASLAIEGD-VEGLV**F**V**V**H**RMEIDFK**A**PA**R**MDD**V**L**T**IET**ATE**KA**GGA**KM**I**L**Q**QQIRR**------GDA**LL**IA**AKV**I**IAVIN**GQGR---PRR**L**PEA

Q89E75_17-142 Q**VRVYF**E**DTD**S**G**Q**IV**Y**HAN**F**L**R**FME**R**GR**T**NYL**RLL**GT**NQQALLEETR(5)FA-**F**V**V**R**SMTIDFL**K**PA**V**LDD**L**L**D**I**V**T**VPQ**EV**RGA**SI**A**L**L**QECRR**------GGD**LLV**S**ARV**R**VAFIS**GG**K**AQRIPKA**L**RLA

Q9A3H0_17-136 P**VRIYY**E**DTD**F**TGIV**Y**HANYL**R**YLE**R**GR**S**DFF**RAV**GI**SHTELAKQDT-----G**F**A**V**I**RMELDFK**R**AA**R**IDD**A**L**L**VRT**LFE**RA**EGV**RL**HVR**QTI**T**R**------GDE**VL**FE**ATV**V**AVCIS**LSGRPRRPTPDMLA

Q9RVW9_12-133 E**LRVRY**A**ETD**A**M**A**V**AH**HATY**PV**WFE**V**AR**T**ELM**HAL**GL**PYTEMETRGY----Y-**L**M**L**SG**LHVQYR**R**AA**R**YDD**R**L**D**ITT**R**I**T**EI**RSR**TL**K**F**A**YEVHR**-IGADGTRE**LLA**T**GET**H**H**I**A**T**D**HQYR---PSR**M**PDD

Q7U4T5_17-132 CK**RVL**PQH**TD**H**AGV**MW**HGAY**VA**WLE**E**AR**V**E**A**L**AAA**GL**SYSAMATMGV----E-**M**P**V**VA**MNLEYR**R**SI**R**HGD**Q**I**V**LE**SHCG**S**QSGV**RW**P**W**RS**CFL**L------DGL**VMA**E**ARV**E**LVIL**GRG**R**----LLRQPPE

Q7VDL8_13-133 RK**TVL**PQN**AD**H**AGV**MW**HGSYL**M**WLE**E**AR**I**N**A**L**SKV**GL**AYSDLSNQGF----E-**M**P**V**V**DLQIKYM**R**SL**L**HGE**E**V**L**LK**SW**I**F**QG**KGP**RW**R**W**KT**DFFN**-----NSGE**IAA**L**ANV**D**LVLIQ**RD**N**SGDRLLREGPE

Q8YPW1_32-151 P**VRVH**PHH**TD**Y**AGIV**W**HGTYL**T**WME**E**AR**V**E**C**L**RSI**GI**EFADLVALGC----D-**L**P**V**V**ELSIRYH**R**SV**Q**LG**MA**V**V**VKA**RMI**DV**TGV**RI**N**W**D**Y**A**I**V**S**----TDGQQ**LFV**T**AKV**T**LVALD**RD**R**GK--IMRQLPS

Q7V171_8-126 EKL**VL**PQH**SD**H**AGV**MW**HGTYF**D**WLE**E**GR**I**N**A**L**SKA**GL**NYVDLTKNGF----D-**L**P**L**I**DTSIKYI**S**PL**F**LGD**T**V**T**IET**IFEISKSP**KI**K**I**HS**KFIN**-----KSKT**ILT**I**AKV**N**LVLIN**KK**S**FS--IIRKRPD

Q88L85_15-132 P**IT**T**RW**H**DND**V**YGHV**N**N**V**VYY**S**FF**DS**A**VN**RLL**VRE**GG**LDIDQGRVI-------**A**L**V**V**S**SA**CDYQ**A**PV**A**FPH**D**I**E**V**G**L**A**V**S**RL**GNT**SV**H**Y**Q**L**A**VF**L----SGQPL**ACA**T**GR**FV**HVFVD**REGRR--PVP**V**PDC

O07408_20-139 P**V**GT**RW**A**DND**M**FGHL**N**NAVYY**QL**F**DT**A**INA**WI**NTS**TG**VDPLAMPVL-------**G**I**V**A**E**SG**CRYF**SE**L**R**FPE**S**L**M**V**G**L**A**V**T**RL**GRS**SV**T**Y**R**L**G**VFK**--EPDDAGV**ITA**L**GH**WV**HVYVD**RT**S**RR--PVP**I**PEA

Q88R80_15-132 P**I**LT**R**PQ**DND**LN**GHI**AG**AT**V**H**G**FFE**T**A**IQA**FL**VEQ**A**ELDLRHGELA-------**A**F**V**V**S**SA**ADFY**ALPG**FPD**L**L**E**V**G**L**G**V**T**RL**AGS**TV**E**Y**R**L**A**LYR**----PGESD**ACA**A**GTV**VQ**VFIE**RA**S**GR--PVA**L**PEA

Q7UJD3_11-130 G**FRV**A**Y**Q**ETD**GQRR**V**H**HANYL**N**YFE**R**GR**V**EML**RDL**GH**NYKAIEDDGR----M-**L**V**V**A**EMNVKYF**A**PA**E**FDD**W**L**E**LTT**T**V**V**EI**RKV**RM**R**H**L**YQIHR**------GDQ**LIV**E**ADS**V**IACVD**RTGKLARLPN**L**ESR

Q8EKL2_13-131 Q**I**P**V**A**W**G**E**M**D**A**L**Q**HV**N**N**V**VYF**R**YFE**T**AR**I**DFF**NRL**F**PLDALYKSGVG------PV**I**S**E**N**QARYK**R**PV**T**FPD**T**L**L**VSV**S**I**S**DI**QSD**RF**T**M**H**YQAFS**----KQQQA**VTT**L**GTS**V**AVM**F**N**FK**T**GQ--KAE**L**PTE

Q8F6U3_12-130 LQ**KV**A**W**G**D**M**D**A**FGHV**N**H**V**VY**AK**YFE**N**AR**A**NYF**TDLK**L**WDTSDTSSQT----G-PV**I**T**HIQVEYR**KQ**V**R**YPD**T**L**E**ITM**Q**V**D**SV**SSR**SF**K**I**SC**TMWN**-----QEGD**CVA**T**ASG**E**F**L**WLN**FV**T**QK--PTQ**L**PEI

Q8PBH4_11-128 P**ISVRW**R**D**M**D**S**MGHV**N**NA**K**YI**S**YLE**E**AR**V**RWM**LGVE**G**VAMTDRIA--------PV**V**AA**TNVNYK**R**PL**V**WP**ND**I**L**VEL**F**V**E**RL**GSS**SV**T**I**G**HRILD**---QKDEGV**LYS**D**GNV**V**VVWID**TQ**T**GK--SAS**L**PDA

Q7NWP0_12-129 T**IS**M**RW**G**D**M**D**A**VGHL**N**N**TY**YF**R**YLE**QV**R**I**EWL**QGM**GF**GIEPDGIG--------PV**L**A**STSCTYR**KQ**L**T**YP**AT**L**D**ITI**E**L**E**KL**GRS**SL**K**L**R**HHFYR**---RDDPGV**VYA**S**AEV**L**LVWVD**YKAEK--SVP**I**PDA

Q9I042_11-128 H**I**P**VRW**G**D**M**D**S**YGHV**N**N**TL**YF**Q**YLE**E**AR**VA**WF**ETL**GI**DLEGAAEG--------PV**V**L**Q**SL**HTYL**K**PV**V**HP**AT**V**V**VEL**YAG**RL**GTS**SL**V**L**E**HRLHT**---LEDPQG**TYG**E**GHC**K**LVWVR**HA**E**NR--STP**V**PDS

Q9HK12_2-115 R**IQIRY**D**D**I**D**Y**LGHV**N**NA**RF**L**A**YFE**I**GR**L**SYM**KRF**F**NTRSAKDIS--------**M**V**I**A**RAELDFE**R**SV**M**F**E**D**D**I**F**VRT**W**I**S**RV**GNR**SF**D**F**S**YTIED**-----DGGT**VFC**R**GRT**VN**VFVE**DG**K**----PVS**V**PDF

Q8EYR4_5-121 P**IQ**T**RW**M**D**M**D**P**F**A**HV**S**NSV**FVA**YLE**I**GR**V**DY**CKRR**L**NVKGIFDVP--------**F**I**L**A**RIEID**L**K**K**SI**E**I**H**H**Q**V**E**VQT**S**V**I**RI**GNK**SW**D**F**QS**KIIE**----TNTKE**IFA**V**AKT**VQ**VA**F**D**HV**N**KS--SIP**I**PHN

Q9HPZ1_9-122 T**VDVRY**Q**D**H**D**T**MGHV**N**NAVY**VT**YME**Q**AR**FA**YL**TDG**IG**RAPTDLD---------**M**V**V**V**NLSVDF**NR**PV**E**FAD**T**V**T**V**G**A**S**I**T**HV**GDT**SF**T**M**A**YEVRD**------DDG**VVA**T**GET**VQ**VALD**PD**T**GQ--PRS**V**PDD

Q7VW65_20-136 D**I**P**LRW**G**DSD**C**L**N**HV**N**N**TI**YF**RM**ME**E**AR**V**RIL**YEA**GY**ELPGQFG---------**V**I**L**A**HASCDFL**R**PL**T**YP**GE**V**R**VTH**T**V**V**RI**GRS**SM**D**L**D**LTLEK**---VGEEGEP**YA**R**SRN**V**LVWVN**YQ**T**NR--AEP**W**PAR

Active Site ---------*-------------------------------------------------------------------------------------------------------------------------

Consensus/80% .hplba.-sD.hGhl.ptsYb.abE.tR.pbb...hh................h.l.phphpab.sh.bsc.l.lph.l.ph...ph.b.bphbp.........hhs.tps.hshlp..p.......h...

**FabA-like dehydratases/synthases (77)**

Secondary St. ----------------EEEEEEEEE---------EEEE---------------------------HHHHHHHHHHHHHHHHHH---------------EEE-----EEEEEEEEEEE----EEEEEEEEEEEEEEE------EEEEE—-EEEE------

O33877/22-167 GPGNA**QLPAP**N**MLMIDRIVHIS**DV**GG**K**YGKGEL**V**AELDINPD**L**WFFACHFEGDPVMPGCLGLDAMWQLV**G**FYLGW**Q**G**N-P**GRG**---------**RALGS**G**EVKF**F**GQVLPTAKKVTYNIHIKR**T**I**NR**S**---**LVLA**I**ADG**T**V**S**VDGR**E**IYSAEGL**R**VGL**F**TS**

O33906/1400-1552 HQPSL**C**F**AS**EK**FLMIE**Q**VSK**V**D**R**TGG**T**WGLGLIEG**H**K**Q**LE**A**D**H**WYFPCHFKGD**Q**VM**A**GSL**MA**EG**CG**QLL**Q**FYMLH**L**GM**HTQTK---NGRFQP**L**EN**AS**QQ**VR**C**RGQVLPQS**GV**LTYR**M**E**V**TEIG**FSPRP-**YAKA**NI**D**--**I**L**L**N**GK**A**V**V**D**F**QNL**G**V**M**I**K**EE**

O33906/1853-2003 -QPHY**RLAGG**Q**L**NF**ID**-S**VEIV**D**NGG**TE**GLGYLYAERTIDP**SD**WFF**QF**HFH**Q**DPVMPGSLGVEAII**ET**M**QA**Y**A**I**SKD**L**GA**DF**K---NPKFGQ**I**LS-**N**I**K**W**KYRGQI**N**P**L**NKQ**M**S**M**DV**S**IT**S**I**KDE**D**G--**K**KVIT**G**N**A**S**L**SK**DG**LR**IYEV**F**DI**AI**SI**E**ES**

P18391/22-167 GAKGP**QLPAP**N**MLMMDRVVKMT**E**TGG**N**FDKGYVEAELDINPD**L**WFFGCHFIGDPVMPGCLGLDAMWQLV**G**FYLGW**L**GG**-E**GKG**---------**RALGV**G**EVKFTGQVLPTAKKVTYRIHFKRIV**NR**R**---**LIMG**L**ADG**E**V**L**VDGR**L**IYTASDL**K**VGL**F**QD**

P45159/28-173 GKEGP**QLPAP**T**MLMMDRI**I**EMN**E**E**T**G**A**FGKGYIEAELDIKPE**LP**FFGCHFIGDPVMPGCLGLDAMWQLV**G**FYLGW**I**GG**-K**GKG**---------**RALGV**G**EVKFTGQILPTAKKV**V**YRIHMKRVI**NR**K**---**LVMG**M**ADG**E**V**E**VDGR**V**IYTATDL**K**VGL**F**QD**

Q7M7J5/23-168 GPGYP**QLPAP**N**MLMMDRVTKMS**E**T**E**G**D**FGKGLILAELDIKPD**L**WFFDCHF**P**GDPVMPGCLGLDAMWQLV**G**FFLGW**V**GG**-K**GKG**---------**RALGV**G**EVKFTGQILPTAKKVTYEIHMKRVV**NR**K**---**LVMG**L**ADG**R**V**L**VDGK**E**IY**V**AKDL**K**VGL**F**QD**

Q7MB46/23-168 GENGPP**LPSG**N**MLMMDRI**I**EMT**E**NGG**T**HDKGYIEAELDITPD**L**WFFDCHFIDDPVMPGCLGLDAMWQLV**G**FFLGW**L**GG**-E**GKG**---------**RALGV**G**EVKFTGQVLPTAKKVTYRI**N**FKRVI**NR**K**---**LIMG**L**ADG**E**V**L**VDGK**I**IYTATDL**K**VGL**F**KD**

Q7U343/27-172 GKEGP**QLPAP**T**MLMMDRVN**L**MT**E**NGG**L**FDKGYIEAELDIHPD**LP**FFGCHFIGDPVMPGCLGLDAMWQLV**G**FFLGW**I**GG**-K**GKG**---------**RALGV**G**EVKFTGQILPTAKKVTYRIHMKRVI**NR**K**---**LVMG**L**ADG**E**V**E**VDGR**V**IYTATDL**K**VGL**F**QD**

Q7U352/23-168 GKKGPV**LPAP**N**MLMIDRITKMT**T**NGG**N**YNKGFV**S**AELDIR**S**D**M**WFFSCHFINDPVMPGCLGLDAMWQLV**G**FYLGW**I**GG**-K**GRG**---------**RALGV**K**EVKFTGQILPTAK**T**VTY**L**IHFRKII**TR**T**---**LIMG**M**ADG**E**V**M**CD**N**K**I**IYTA**A**DL**K**VGL**F**TN**

Q7WYA1/657-811 VQKSL**C**F**AS**EK**FLMIERVSQL**QT**QGG**T**WGLGLLEG**H**K**H**I**A**PD**H**WYFPCHFQGD**Q**VM**A**GSL**MA**EG**CG**QLL**Q**FFMIH**I**GM**HTLVE---NGRFQP**L**KN**AS**Q**KVR**C**RGQVLPQT**A**ELTYR**M**E**V**SEIG**IHPRP-**YAKA**NI**D**IL**I**L**LDGK**V**V**V**D**F**QNL**G**V**M**I**K**ED**

Q820X5/17-160 --GIPA**LPAP**P**LLM**V**DRV**L**S**VEH**TG**---**SRGKIIAE**T**DIH**L**D**D**WFF**Q**CHFR**H**DPV**K**PGCLGVDAIWQLL**GL**YI**SLR**GG**-V**G**S**G**---------**RALGA**K**EV**D**F**F**GQI**R**PHNK**V**VRYEV**S**IRR**YSHL**E**QQGVS**M**VL**GDG**S**V**F**VDGE**Q**VYQ**I**N**QAK**VG**SFL**N**

Q87B86/23-169 SHSNA**RLPND**P**MLMFDRITEI**YA**DGG**S**HGKGIV**N**AELDIRPD**L**WFFGCHFLGDPVMPGCLGLDAMWQLT**G**FFLTW**S**GA**TP**GYG**---------**RALGC**G**EVKFTGQVLPNAKLVRYEVEMTKII**NR**T**---**LVIG**Q**A**N**A**RML**VD**N**R**E**IY**F**AKDL**R**VG**MF**NN**

Q87PC5/23-168 GPGYP**QLPAP**N**MLMMDRVTKMS**E**T**E**G**D**FGKGLILAELDITPD**L**WFFDCHF**P**GDPVMPGCLGLDAMWQLV**G**FFLGW**V**GG**-K**GKG**---------**RALGV**G**EVKFTGQILPTAKKVTYEIHMKRVV**NR**K**---**LVMG**L**ADG**R**V**C**VDGK**E**IY**V**AKDL**K**VGL**F**QD**

Q883Y6/22-167 GPGNA**QLPAP**N**MLM**V**DRITHIS**E**EGG**K**FGKGEL**V**AELDINPD**L**WFFACHFEGDPVMPGCLGLDAMWQLV**G**FYLGW**Q**G**N-P**GRG**---------**RALGS**G**EVKF**F**GQVLPTAKKVTYNI**Q**IKR**T**M**RG**K**---**LVLA**I**A**E**G**T**V**S**VDGR**E**IYSAEGL**R**VGL**F**TS**

Q88FC4/22-167 GPGNA**QLPAP**N**MLM**V**DRITHIS**E**EGG**K**YGKGEL**V**AELDINPD**L**WFFACHFEGDPVMPGCLGLDAMWQLV**G**FFLGW**Q**GL**-P**GRG**---------**RALGS**G**EVKF**F**GQVLPEAKKVTYNIHIKRVL**KG**K**---**L**N**MA**I**ADG**S**V**S**VDGR**E**IYTAEGL**R**VGV**F**TS**

Q89M47/29-174 GPGNA**QLPAP**P**MLMMDRISEIS**L**DGG**E**FGKGHI**V**GELDI**V**P**GH**WFFDCHFRGDP**L**MP**S**SLGLDAMWQ**M**I**G**YWLGW**S**G**S-P**GKG**---------**RAIGV**G**EV**EC**TG**E**ITPTV**Q**RVRYEV**A**MR**M**V**RRG**K**---**LVLG**I**ADG**R**V**L**ADG**AC**V**F**TAKD**MR**VGL**T**K**A

Q89WC3/23-168 GPGNA**QLP**L**P**P**MLMFDRITDIN**D**NGG**E**FGKGLVRAELDVKPD**L**WFFGCHFKNDPVMPGCLGLDALWQ**M**V**G**FYLGW**S**GG**-E**GRG**---------**RALG**LN**ELKFSGQVLPEARKV**V**YNVDIKRVM**RA**K**---**LVLG**I**ADG**W**L**S**VD**D**Q**I**IYRAKDL**K**VGL**F**KQ**

Q8D2Q4/23-168 GKYGP**QLPAP**N**MLMIDRLVK**V**T**E**NGG**N**YNKGFIKAELDINP**NM**WFFSCHFIGDPVMPGCLGLDAMWQLV**G**FYLGW**L**GG**-K**GKG**---------**RALGV**R**EVKFSGQILPTSKIV**V**Y**Y**IHFRRII**NR**K**---**LFMG**M**ADG**E**V**F**CDGK**I**IYTANDL**K**VGL**F**QD**

Q8D9H3/23-168 GPGYP**QLPAP**N**MLMMDRVTKMS**E**T**E**G**D**FGKGLILAELDIKPD**L**WFFDCHF**P**GDPVMPGCLGLDAMWQLV**G**FFLGW**V**GG**-K**GKG**---------**RALGV**G**EVKFTGQILPTAKKVTYEIHMKRVV**NR**K**---**LVMG**L**ADG**R**V**L**VDGK**E**IY**V**AKDL**K**VGL**F**QD**

Q8EFV9/22-167 GPNSP**RLPVD**N**MLMIDRI**I**TIN**D**NGG**E**FGKGEI**V**AELDIKPE**L**WFFDCHFITDPVMPGCLGLDAMWQLV**G**FYLGW**E**GA**-E**GKG**---------**RALGV**G**EVKFTGQVLP**G**AKKVTYKL**N**IKR**T**I**HR**K**---**LVMG**I**ADA**I**L**E**VDGR**Q**IYSATDL**K**VGV**F**SD**

Q8EGK2/1360-1517 LQPSL**C**F**AS**EK**FLMIE**Q**VSKL**EV**HGG**A**WGLGLIEG**H**K**Q**L**A**PD**H**WYFPCHFKGD**Q**VM**A**GSL**MA**EG**CG**QLL**Q**FFMLH**I**GM**HA**N**TQ(5)NGRFQP**L**EN**AS**Q**KVR**C**RGQVLPQS**GT**LTYR**M**E**V**TEIG**MSPRP-**YAKA**NI**D**--**I**L**L**N**GK**V**V**V**D**F**QNL**G**V**M**I**K**EE**

Q8EGK2/1813-1963 -KPHY**RLAGG**Q**L**NF**ID**-K**AEIV**K**TGG**KK**GLGYLYAERTIDP**SD**WFF**QF**HFH**Q**DPVMPGSLGVEAII**E**LL**QT**Y**A**I**DQD**L**GA**GFN**---NPKFGQ**I**LS-EI**K**W**KYRGQI**N**P**L**NKQ**M**S**L**DVHIT**S**I**EDK**D**G--**K**R**I**IK**GDA**N**L**SK**DG**LR**IYEVTDI**AI**CI**E**E**A

Q8FJ83/22-167 GAKGP**QLPAP**N**MLMMDRVVKMT**E**TGG**N**FDKGYVEAELDINPD**L**WFFGCHFIGDPVMPGCLGLDAMWQLV**G**FYLGW**L**GG**-E**GKG**---------**RALGV**G**EVKFTGQVLPTAKKVTYRIHFKRIV**NR**R**---**LIMG**L**ADG**E**V**L**VDGR**L**IYTANDL**K**VGL**F**QD**

Q8PCW9/22-167 GPNSG**RLPND**P**MLMFDRITEIN**D**NGG**S**HGKGLIRAELDIRPD**L**WFFNCHFIGDPVMPGCLGLDAMWQLT**G**FFLTW**I**GA**-P**GRG**---------**RALGC**G**EVKFTGQVLPTA**T**LVTYEIEISRVI**NR**K**---**LVMA**Q**SDA**RML**VDGR**E**IY**A**AKDL**R**VG**MF**TS**

Q8PGJ2/22-167 APDSG**RLPND**P**MLMFDRITEIS**N**TGG**A**HGKG**V**IRAELDIRPD**L**WFFGCHFIGDPVMPGCLGLDAMWQLT**G**FFLTW**I**GA**-P**GRG**---------**RALGC**G**EVKFTGQVLPSARLVRYEID**V**SRVI**NR**K**---**LVMA**QT**DA**RML**VDGR**E**IYTAKDL**R**VG**MF**TS**

Q8UIZ0/37-182 GPGNA**QLP**L**P**P**MLM**VH**RITEIS**E**TGG**A**FDKGFIRAEYDVSPD**D**WYFPCHFQG**N**P**I**MPGCLGLDGMWQLT**G**FFLGW**L**G**E-E**GRG**---------**MALST**G**EVKFKG**M**V**R**PNTKLLQY**G**IDFKRVM**RG**R**---**LVLG**T**ADG**W**L**K**ADGE**T**IYQASDL**R**VGL**S**KE**

Q8XEY3/22-167 GAKGP**QLPAP**N**MLMMDRVVKMT**E**TGG**N**FDKGYVEAELDINPD**L**WFFGCHFIGDPVMPGCLGLDAMWQLV**G**FYLGW**L**GG**-E**GKG**---------**RALGV**G**EVKFTGQVLPTARKVTYRIHFKRIV**NR**R**---**LIMG**L**ADG**E**V**L**VDGR**L**IYTAHDL**K**VGL**F**QD**

Q8YEC2/23-168 GPGNA**QLP**L**P**P**MLMI**H**RITEIS**E**TGG**A**FDKGYIRAEYDVRPD**D**WYFPCHFQG**N**P**I**MPGCLGLDGMWQLT**G**FFLGW**L**G**E-P**GRG**---------**MALST**G**EVKFKG**M**V**R**PHTKLLEY**G**IDFKRVM**RG**R**---**LVLG**T**ADG**W**L**K**ADGE**L**IYQATDL**R**VGL**S**KE**

Q8YWH0/1054-1206 INPSL**RLPPA**K**LLMLD**N**V**MMV**N**P**QGG**VA**GLGL**A**IG**S**K**E**VTPE**D**WY**YF**CHFRNDP**T**MPGNL**M**IEG**C**IQLV**Q**FY**C**LF**L**GL**QTRTK---DARFQI**IPG**K**T**QAA**RFRGQVTPQT**GT**L**M**YQ**M**E**VL**ELG**LSPQP-**YA**V**A**NV**D**--**V**I**F**G**GK**T**I**A**T**I**KNI**G**V**Q**L**V**EK**

Q8YWH0/1450-1601 -KPHY**RLS**EKY**L**DF**LD**-EML**I**IEG**GG**N**Y**Q**KGYIYA**R**KSITP**QD**WYFP**F**HFY**Q**DPVMPGALGVESIIQ**A**M**QA**Y**A**L**QLD**L**GK**SF**R---NPRFGQAINHEITW**KYRGQITPENHL**M**S**L**EVHISKI**EVE**S**E--**RI**TII**ADA**S**L**WKEDLR**IYE**I**KDI**AL**CL**V**E**A

Q8ZG80/23-168 GAGGPP**LPAG**N**MLMMDRIVKM**IE**DGG**S**HNKGYVEAELDINPD**L**WFFGCHFIGDPVMPGCLGLDAMWQLV**G**FYLGW**L**GG**-E**GKG**---------**RALGV**G**EVKFTGQVLPDAKKVTYRI**N**FKRVI**MR**K**---**LIMG**V**ADG**E**V**L**VDGK**V**IYTATDL**K**VGL**F**KD**

Q92SV8/23-168 GPGNA**QLP**L**P**P**MLMF**N**RITDIS**E**TGG**PN**DKGYVRAEFDITPD**L**WFFPCHFMGDPVMPGCLGLDAMWQLT**G**FFLGW**L**G**E-A**GKG**---------**RAIST**G**EVKFTG**M**VTPKTKLVEY**G**IDFKRVM**RG**R**---**LVLG**I**ADG**WMK**ADGE**T**IYKATDL**R**VGL**F**QE**

Q93CG6/1338-1490 HQPSL**C**F**SSD**K**F**M**MIE**Q**ISH**V**D**P**QGG**T**WGLGLIEG**H**K**Q**LE**A**D**H**WYFPCHFKDD**S**VM**A**GSL**MA**EG**CG**QLL**Q**FFMMY**L**GM**HTQVE---NGRFQP**L**EN**AP**QQ**VR**C**RGQVLPQS**AV**LTYR**M**E**V**TEIG**LSPRP-**YAKA**NI**D**--**I**L**LDGK**V**V**V**D**F**QNL**G**V**M**I**K**EE**

Q93CG6/1807-1958 AQPHY**QLAGG**R**L**NF**ID**-K**VDIT**S**DGG**KA**GLGYLYAERTIDP**SD**WFF**QF**HFH**Q**DPVMPGSLGVEAII**E**LM**QT**Y**A**L**NKD**L**GA**GF**R---SPKFGQ**I**QS-EV**K**W**KYRGQI**N**P**L**NKQ**M**S**L**DVHIT**A**I**KDE**D**G--**K**R**I**IV**GDA**N**L**SK**DG**LR**IYEVKDI**AI**CI**E**E**A

Q93PM8/203-353 DKPHW**RLPPG**H**R**FR**L**L**H**Q**ASLV**R**EGG**K**FGLGYVKGERTID**AGE**WYFT**N**HFH**R**DPVMPGSLGLEAILQ**A**M**QL**F**A**I**RT**GL**DA**GIA**N---PRFGIA**VGVP**VNW**RYRGQLL**R**TDKR**MGL**EVHIKEI**RREGE--G**L**VVI**AD**TD**L**FN**D**RLR**IYEA**L**S**MSI**SI**---

Q94FB6/267-419 PEINY**KLCA**RK**MLMIDRVTSID**H**KGG**V**YGLGQL**V**GEK**I**LE**R**D**H**WYFPCHF**VK**D**Q**VM**A**GSL**VS**DG**CS**Q**M**L**KM**YMIW**L**GL**HL**T**T**G**---PFDFRPV**NG**H**P**N**KVR**C**RGQI**S**PH**KG**KL**V**Y**VM**EIKE**M**G**FD**E**DN-DP**YA**I**AD**--**V**N**I**IDVDFE**KGQD**FSL**D**RI**SD**

Q94FB6/795-950 PSGAQLNRR**T**D**Q**GQ**Y**L**D**A**VDIV**SGS**G**KK**SLGY**A**HG**S**KTVNP**ND**WFFSCHFW**F**D**S**VMPGSLGVESMFQLV**EAIA**AH**ED**L**AGKAR-HCQPHLCA**RP**R**A**RSSW**KYRGQLTPKSKK**M**D**S**EVHI**VS**V**DAH**D**G--V**V**DLV**ADG**F**L**W**AD**SLR**VYSVSNI**R**V**R**I**A**S**G

Q98BH8/22-167 GEGNA**QLP**Y**P**P**MLMFDRITEIS**E**TGG**A**FDKGFIRAEFDIKPD**L**WFFACHFIG**N**P**I**MPGCLGLDALWQLT**G**FYLGW**L**G**E-P**GKG**---------**MALST**G**EVKFKG**M**VTPSVKKVEY**G**VDFKRVM**RG**R**---**LVLG**I**ADG**WMK**ADGE**P**IY**A**ATDL**K**VGL**S**KQ**

Q9A246/21-166 GPGNA**QLPAP**P**MLMFDRIVRI**EA**EGG**K**YGKGYVEAEFDIRPD**L**WFFDCHFIGDPVMPGCLGLDAMWQLV**G**FFLGW**S**GG**-P**GRG**---------**RALGV**G**EVKFTGQVTPD**I**KKV**V**YKIDLKRVI**MR**K**---**LVMG**I**ADG**V**L**E**ADGK**V**IYETSDL**K**VGL**F**T**P

Q9CNE8/28-173 GEDGP**QLPAP**T**MLMMDRIVEMN**E**TGG**Q**FNKGYIEAELDIKPD**LP**FFGCHFIGDPVMPGCLGLDAMWQLV**G**FYLGW**I**GG**-K**GKG**---------**RALGV**G**EVKFTGQILPSAKKV**V**YRIHMKRVI**NR**K**---**LVMG**M**ADG**E**V**E**VDGR**V**IYTATDL**K**VGL**F**QD**

Q9KS00/23-168 GEGYP**QLPAP**N**MLMMDRITKMS**E**T**E**G**E**FGKGLILAELDITPD**L**WFFDCHF**P**GDPVMPGCLGLDAMWQLV**G**FFLGW**V**GG**-K**GKG**---------**RALGV**G**EVKFTGQILPTAKKVTYEI**N**MKRVV**NR**K**---**LVMG**L**ADG**R**V**L**VDGK**E**IY**V**AKDL**K**VGL**F**QD**

Q9PFT5/23-169 SHSNA**RLPND**P**MLMFDRITEI**YA**DGG**S**HGKGIV**N**AELDIRPD**L**WFFGCHFLGDPVMPGCLGLDAMWQLT**G**FFLTW**S**GA**TP**GYG**---------**RALGC**G**EVKFTGQVLPNAKLVRYEIEMTKII**NR**T**---**LVIG**Q**A**N**A**RML**VD**N**R**E**IY**F**AKDL**R**VG**MF**NS**

Q9RA19/1393-1545 RNPSL**K**F**SS**EK**FLMIERITKID**P**TGG**H**WGLGLLEG**Q**KDLDPE**H**WYFPCHFKGD**Q**VM**A**GSL**MS**EG**CG**Q**M**A**M**FFML**SL**GM**HT**N**V**N**---NARFQP**LPG**E**S**QT**VR**C**RGQVLPQ**RNT**LTYR**M**E**V**T**AM**G**MHPQP-**FMKA**NI**D**--**I**L**LDGK**V**V**V**D**F**KNL**S**V**M**I**S**EQ**

Q9S1Z9/1669-1821 HVRTPL**LDG**ER**L**RL**LDEVT**V**FD**PV**GG**P**WGRGYLRAE**T**AVSPD**D**WFFT**G**HFKNDP**C**MPGTL**M**L**Q**G**G**LQ**A**M**A**FYLT**AS**GH**TV**DRD**---GWRFEPV**DT**Q**P**S**R**T**K**C**RGQ**A**TPESRRI**V**YEL**FV**R**G**V**SAGP---VPTLH**AD**VLGS**VDGR**KAFL**GRG**MALR**L**VP**D**

Q9S1Z9/2082-2235 -AGEP**RLPG**RM**LLMLDRVT**GSWP**EGG**TA**GLGRLRSEKDVHPD**A**WFF**RA**HFF**Q**DPV**Q**PGSLGVEAM**C**QLL**Q**YHLI**EG**GA**AD**GIA**HP--RFEPV**LPG**RETVWT**YRGQITP**A**NRLIR**V**D**M**DI**V**E**T**G**TDAR--GP**YA**V**ADA**T**L**W**GD**D**T**C**IYRVRGL**GMR**V**V**S**G

Active Site ------------------------------------------------*-------------*------------------------------------------------------------------------------------------------

Consensus/80% .....pLsss.bLMb-clspbs.pGG.asbGblbtEbslpP-.WaFsCHFbsDPVMPGsLGl-tbbQLh.aabha.Gh..sbs.........bshss.cl+apGQlhPps+blpYplcbpclh..p...bhbt.tDt.l.hDGp.lYpspsl.Vsl.pp

**Fat subfamily (73)**

Secondary St. ----EEEEEEEEE-------E-HHHHHHHHHHHHHHHHHHH-----------------EEEEEEEEEEE---------EEEEEEEE----------EEEEEE------HHHHHEEEEE----------HHHHHHHH-------------E----------

Q43718_79-362 GYS**YKEKF**I**V**RS**YEVGIN**K**TATV**E**TI**A**NLL**Q**EVACNHVQKCGF**S**T**DGFATTLT**M**R**K**LH**LIWVTARMHI**E**I**Y**KYP**A**W**S**D**V**V**E**IETWC**Q**SE**G**RIGTRR**D**WILRD**SA**T**N**E**V**IGRATS**K**WVMMN**Q**DTRRL**Q**RV**TD**EV**RDE**Y**LVFCPREPRLAFP**EE**NN**SSLKKI**

Q9ZTF7_85-372 GLS**YTEKF**I**V**R**CYEVGIN**K**TATV**E**T**MA**NLL**Q**EVGCNHAQSVGF**S**T**DGFATTPT**M**R**K**LN**LIWVTARMHI**E**I**Y**KYP**A**W**S**D**V**V**E**IETWC**Q**SE**G**RIGTRR**D**WILKD**YG**NGE**V**IGRATS**K**WVMMN**QN**TRRL**Q**KV**DDS**V**REE**Y**MVFCPREPRLSFP**EE**NN**RSLRKI**

Q41634_103-363 GLV**FRRTF**A**I**R**C**S**EVG**P**D**R**STSI**V**AVMNYL**Q**EAACNHAESLGL**L**G**DGFGETLE**M**S**R**RD**LIWVV**R**R**T**H**VV**VERYP**A**W**G**D**T**V**E**VE**A**W**IG**A**AGN**IG**M**RR**H**FLVRD**CK**TGH**I**LARCTS**VS**VMMN**M**RTRRL**S**KI**PQ**EV**RGE**I**DPLFIEKFAV--K**E**GEI**KKLQKF**

Q9XH14_141-405 GLV**FR**Q**NF**S**I**RS**YE**I**GAD**R**TASI**E**TLMNHL**Q**ETALNHVRNAGL**L**G**DGFGATPE**M**S**K**RN**LIWVVTKMQ**VL**IEHYP**S**W**G**D**V**V**E**VDTWV**G**A**SG**K**N**G**M**RR**D**WHVRD**YR**TGQ**T**ILRATS**I**WVMMD**K**HTRKL**S**K**MPE**EV**RAE**I**GPYFMEHAAI--V**DE**DS**RKL**P**KL**

Q8XH69_3-250 ENK**F**M**KKY**D**V**L**YYDSDVN**E**NIR**MV**PLM**K**IF**G**DVSA**I**H**E**EELAY**E**G**IKY-----**L**K**D**HE**L**S**W**I**IYSYSI**D**IKK**PIP**Y**KSS**I**N**VETY**LE**GI**K**KFYA**C**R**V**YKVYN**-E**K**N**E**L**VAEGK**II**FLLID**L**EKRRA**V**RI**PK**E**YCEL**I**N-----------M**SD**TG**E**V**ELK**S

Q8XH68_2-246 GKA**YEK**V**Y**E**V**T**Y**G**ETDGR**K**DCRI**T**S**M**MNFF**S**DC**C**LSQ**E**EK**N**SM**NYAD-------N**S**SET**TWVFFDYEI**I**VNRYP**R**Y**R**E**K**I**K**VKTYV**E**SI**R**KFYS**N**R**V**FE**A**YD**-M**DG**AL**VARAD**VLA**FLIN**K**KTRR**PA**RI**SD**E**EYEI**H**GL----------S**KE**SS**K**L**LRK**K

Q899Q1_2-246 -SKM**EKDY**E**I**H**YYEVDYK**K**RA**L**I**T**SIINYL**G**DIATKQSEDM**NVGLKY------**M**E**E**NK**IAWVIYKWDI**N**IK**E**FP**V**Y**G**D**I**V**K**IKT**S**P**KC**F**K**KFYAYR**D**FEVIN**-S**KGE**K**IIEA**L**S**Q**WLLID**T**DKRRL**K**KI**PL**EL**FKF**Y**G-----------V**ED**VE**CE**D**I**E**I**

Q97D89_3-247 KVVT**KRNY**DTH**IYEVDFK**G**KATI**T**SIMSYL**E**DIAT**Y**QTDNLGM**S**V**QY------**L**I**D**NK**LAWVVYKWEI**HM**DKYP**E**L**G**D**T**I**E**V**A**TIP**Y**SI**R**KFYAYR**K**YELFN**--**KGE**K**IGYANS**L**WFLID**T**ERRR**PC**RV**TE**EI**YKI**Y**N-----------L**TE**ED**DEQI**P**F**

Q838S0_2-244 GKK**HT**S**SY**E**V**A**YYDGDFT**GA**MKI**P**ALLA**V**V**I**KVS**E**EQTE**L**LGR**D**A**AY------**V**A**Q**FG**LGWVITNYEI**E**IHRLP**KVG**E**K**V**A**ITTQA**M**SY**N**KYFCYR**N**FWVHD**-E**EGK**EC**VFVKS**T**FVLMD**Q**KNRKI**S**SV**LP**EI**IAP**F**------------D**SE**KI**TKIYRH**

Q88YP1_8-255 ASL**YSEQH**R**I**T**YYECD**R**T**G**RATL**T**TLIDIA**VL**AS**E**DQSD**A**LGL**T**T**EM------**V**Q**S**HG**VGWVVTQY**A**I**D**ITRMP**R**Q**D**E**V**V**T**I**AV**RG**S**AY**NP**YFAYR**E**FWIRD**-A**DGQ**Q**LAYITS**I**WVMMS**QT**TRRI**V**KI**LP**EL**VAP**Y**------------Q**SE**VV**KRI**P**RL**

Q8E045_2-241 GLL**YRETY**E**V**P**FYESDTN**HY**MKL**PQ**LLALA**L**QISAKQS**L**KLGI**G**D**DIV-----**F**K**R**YG**LVWVVTDY**I**I**D**IERLP**K**H**A**E**K**I**V**IETEA**K**AH**N**KLLCYR**Y**FYIYG**-E**DGQ**K**II**T**ISS**A**FVLMD**F**KTRKI**HP**V**LD**DI**TSI**Y**------------Q**S**QRI**KK**V**IRG**

Q8P176_2-241 GLS**YQEE**LT**L**P**FE**L**CDVK**S**DIKL**PL**LLDYC**LM**VSGRQS**A**QLGR**S**N**NNL-----**L**V**D**YK**LVW**I**VTDYEI**T**IHRLP**H**F**Q**E**T**I**I**IETKA**L**SY**N**KFFCYR**Q**FYIYD**-Q**EG**GL**LV**D**I**L**A**Y**FALLN**P**DTRKV**A**TI**PE**DL**VAP**F**------------E**TD**FV**KKLHRV**

Consensus/80% ...apcpa.l.hb-sshp.phpl.slbsbh.phthppspphtb.s........h.p..lhWVhhpbpI.lp+bP.b.-.l.lpTbs.tb.+bhsbR.ablbs..pGp.lhbhpt.ahbbs.cpR+h.pl..-l...b.............p-..ppbb+h

Secondary St. -----------------EEEEE----------HHHHHHHHHHHHHHHHHH------EEEEEEEEEE----EEEEEEEE------------------EEEEEEEEE-----EEEEEEEE---

Q43718_79-362 P**K**---L**E**DPA**Q**YSMLE**LKPRR**A**DLDMN**Q**HVNNVTYI**G**WVLES**-IP**QEI**I**D**T**HE**L**QV**I**TL**D**YR**REC**Q**Q**D**D**I**VDS**LTT**SEIPDDPIS(11)SIQGHNESQ**FLHML**RL**S**EN**G**QE**INRGRTQWR**K

Q9ZTF7_85-372 S**K**---L**E**DPA**E**YSRLG**LTPRR**A**DLDMN**Q**HVNNV**A**YI**G**WALESVP**Q**EII**D**SYEL**E**TI**T**L**D**YRREC**Q**QDD**V**V**DSL**TSV**LSDEESGTL(14)KRDHDGSRQ**FLHLL**RL**S**PD**G**LE**INRGRTEWR**K

Q41634_103-363 -----N**D**STA**D**YIQGG**WTPRW**N**DLDVN**Q**HVNN**I**KYV**G**WIF**K**S**-**V**P**D**S**I**Y**E**N**HH**L**S**SI**TL**E**YR**RECTRGRALQS**LTT**VC-----------GGSSEAG**I**I**CEHLL**QL**E**D-**G**SE**V**L**RGRTDWR**P

Q9XH14_141-405 -----D**D**DTA**D**YIKWG**LTPRW**S**DLDVN**Q**HVNNVKYI**G**WILESAP**I**SIL**E**NHEL**A**SM**T**LEYRREC**G**RDS**V**L**QSL**T**A**V**AND----CT----GGLPEAS**I**E**CQHLL**QL**E**C-**G**AE**IVRGRTQWR**P

Q8XH69_3-250 T**K**V--E**K**LIR**E**DLESN**ISVRR**S**DIDFN**K**HVNNTKYL**E**WTMEATP**E**CIL**D**EYSL**I**SA**K**IKYEKEV**R**LGD**D**V**NII**CQW**DEIEE--------------G**Y**K**CL**Y**KI**VN**N**RL**G**EVS**A**SI**ET**I**WK**K

Q8XH68_2-246 L**N**F--E**K**FDK**E**DLDMK**FH**I**RY**L**DIDLN**M**HV**S**N**I**KYV**E**WILETVP**V**DIV**L**NYKM**K**KI**K**IKFEKEI**T**YGH**N**V**IIKS**KI**IKGED--------------E**V**K**VLHKV**EN**E**E-**G**ES**ITLAET**Y**W**Y-

Q899Q1_2-246 S**K**I--N**E**LKKVDNEKI**FNVRY**S**DIDTN**G**HVNNSKYI**S**WIIETVP**L**EIV**L**NYSL**K**NL**N**M**T**YKKET**V**YGD**M**V**RVL**CEI**ENKDD--------------M**A**I**CRH**S**I**AD**K**N-**N**NE**LNIAETTWQ**K

Q97D89_3-247 E**K**L--S**K**PKEVSFENR**FKVRY**S**DIDTN**R**HVNNVKYV**S**WVLENIP**L**E**VMK**DYEI**S**DL**K**VMYQKET**A**YGE**T**I**DIV**TEM**KKSEG--------------K**C**S**F**N**HLI**TN**S**Q-**G**EN**LTL**I**KTD**FIK

Q838S0_2-244 E**K**---I**E**KVT**E**GNFLP**YRVRF**F**DIDGN**Q**HVNNA**I**Y**FN**WLLDVLG**Y**DFL**T**THQ**PK**KI**L**VKF**D**KEV**E**YGQ**E**V**ESH**YEI**VEQEN--------------Q**L**K**TRHEI**RI**D**--**G**QT**YCEANIDWT**N

Q88YP1_8-255 P**R**PISF**E**ATD**T**TITKP**YHVRF**F**DID**P**N**R**HVNNAHY**FD**WL**V**DTLP**A**TFL**L**QHDL**V**HV**D**VRYE**N**EV**K**YGQ**T**V**TAH**ANI**L------------PSEVADQ**V**T**T**S**HLI**EV**D**--**D**EK**CCEVTIQWR**T

Q8E045_2-241 P**K**---Y**H**PIG**D**SKVKQ**YHVRY**F**DLDMN**G**HVNNSKYL**E**WMYDVLD**L**DFL**S**SH**IPK**KI**D**LKYIKEI**Q**YGT**D**I**KSH**W**YQD------------------G**L**V**TRH**D**I**IGG--**D**AI**HAQARIEWQ**E

Q8P176_2-241 P**K**---MPLLE**Q**SIDRD**Y**Y**VRY**F**DIDMN**G**HVNNSKYL**D**WMYDVLG**C**EFL**K**THQL**L**KM**T**LKY**V**KEV**SP**G**GQ**I**TSS**YHL**D------------------Q**L**TS**YHQI**IS**D**--**G**QLN**AQA**M**IEWR**A

Consensus/80% .p....c...p.....bpsRb.DlDhN.HVNNspYl.Whb-shs.pbl.papb.ph.hbab+Eh.bsp.l...hph....................h.hbHbl..p..s..hsbsphpWp.

**TesB subfamily domain 1 (66)**

Secondary St. ------------------------------HHHHHHHHHHHHHH---------E-EEEEEE------EEEEEEEEE------EEEEEEEEE----EEEEEEEEE------------------

Q18061_11-128 **KM**Y**T**FEK**ID**E**N**N**FRA**DYLFH**A**RQNPNGS**AYGGLLFSQALAAA**E**NTV**SD**EF**K**PNS**-**IHAYFV**S**A**VIYST**P**A**IYKVKRI**K**DGKTF**II**RTV**E**AVQNDR**IC**F**V**L**Q**VSFH**ISE**KSG**---**M**I**H**QDI**MP**

Q9BIA5_42-159 **KF**F**D**FTE**LK**K**D**S**FSP**STLSN**G**RQAHHGA**AYGGLIFSQALAAA**E**KTV**DE**QF**K**PHS**-**MHSYFI**L**N**V**D**TKE**PI**S**YNVRRIRDGRSF**I**TRTV**E**AVQKDK**VC**F**V**L**Q**CSFH**VEE**KSS**---**I**I**H**QSE**MP**

Q19781_35-151 **TFLNL**QR**ID**T**N**LYI**A**RHLLK**G**RHSY-NA**VYGGQVVGQSLAAA**AA**TV**ED**CFIPHS**-**LHSYFI**K**TG**SVDK**PILY**MI**DRIRDGRSFCTR**V**V**K**AVQDGE**A**IF**S**C**Q**ISFH**HKEP**DA**---**IKH**SSK**MP**

O14734_30-143 **TVLNL**EP**LD**E**D**L**FRG**RHYWV**P**A----KR**LFGGQIVGQALVAA**A**K**S**V**SE**D**V**HVHS**-**LHCYFV**R**AGD**PKL**PVLYQVERTR**T**GSSFSVRSV**K**AVQHGK**P**IF**I**C**Q**ASFQ**QAQP**S**P---**MQH**QFS**MP**

O06135_15-128 A**VLDL**NA**V**AS**D**L**FTG**SHPSK**N**P----LR**TFGGQLMAQS**F**VA**SS**RT**LTR**HHLP**P**S**A**F**S**V**H**FI**N**GGD**TAK**DI**E**FQV**I**RLRD**E**R**R**FANRRV**D**AVQDGT**L**L**SS**A**M**VS**YMA-GG**RG**---**HEH**ALDP**P**

P41903_13-132 **KILEL**VP**LS**P**T**S**F**V**T**KYLPA**A**PVGS-KG**TFGG**T**LVSQSLLA**SL**HTV**PL**NFFPTS**-**LHSYFI**K**GGD**PRTK**ITYHV**Q**NLR**N**GRNF**IHK**QV**S**AYQHDK**L**IF**TSM**I**L**F**AVQR**SK**EHDS**LQH**WET**IP**

Q8SRT9_6-121 **EFIQ**MVR**LD**S**G**V**FEG**SNLWS**P**LPG--YP**AFGGQIAAQSLA**S**A**F**STV**GE**D**S**VPN**T-**M**SIL**FI**NKC**K**SDQK**V**K**YTVKNL**KN**GS**IV**D**M**RQV**DC**YQNN**VL**V**SS**A**H**ISFS**KPD**NNA**---**HD**YEGTPY

Q8G3R7_15-132 **KVLKL**GEP**S**AYRN**HT**YINGE**S**MYFPTGR**VYGGQVIAQS**V**IAA**S**KTV**GP**SRLPHS**-V**HGYFI**A**AGD**IRQ**DLLFDVENLRDGRSFSARR**INV**TQ**AEGS**IL**T**A**I**ASFQ**ETG**QEG**---V**E**FADP**MP**

Q7SI70_34-155 **EVIEL**AV**I**GP**N**I**FTN**ARQPWHPPGA-RG**IYGG**A**VIA**MC**LAA**GQ**KTV**PS**DFLVHS**-**MHCYFL**L**AG**SAEL**PILFHVE**L**VRDGRSFATRTV**Q**A**R**QKGR**C**IF**T**T**T**ISF**VREG**SSG**(5)V**SH**ASS**MP**

Q89WS4_22-134 **SILDL**EQ**LE**V**N**L**FRG**NSPKT**S**W----QR**VFGGQVIGQA**M**VAA**C**RTV**E-G**RLPHS**-**LHCYFI**L**PGD**PQI**PIIYQVERLRDGKS**Y**STRRV**T**AIQHGN**A**IF**S**I**M**VSFH**AEE**ESA**---**FDH**QDK**MP**

Q8FYH7_20-132 **SILDL**ET**LE**M**D**L**FRG**NSPQV**G**W----QR**VFGGQVIGQALIAA**Q**RTV**DP**ERHVHS**-**LHAYFV**R**PGD**PAI**PIIYEVDRIRDGSSFSTRRV**L**A**K**QHGK**A**IF**T**L**S**ASFQ**I-D**E**G**G**---**LDH**QMP**MP**

Q82CR2_10-122 **DLLDL**ER**IE**Q**D**I**FRG**QSRSA**V**V----PR**VFGGQVAAQALVAA**G**RTV**PE**DRLAHS**-**LHAYFL**R**AGD**PGA**PIVYTVDRIRDGRSFTTRRV**V**AVQHGQ**P**IF**H**L**S**ASFQ**T-Y**EEG**---**MEH**QAD**MP**

Q8FPK1_28-141 Y**IL**G**L**EE**ID**R**D**IY**RG**PVVES**A**L----SR**TFGGQVAAQ**T**LVAA**T**RTV**DP**EFAVHS**-**LHGYFL**R**AGK**SDR**P**T**VF**L**VDRMRDGRSFSSR**M**V**R**AIQDGE**T**IF**S**M**Q**ASFH**RRG**DEG**---**IEH**ADT**M**R

P23911_9-121 **TLLNL**EK**IE**E**G**L**FRG**QSEDL**G**L----RQ**VFGGQVVGQALYAA**K**ETV**PE**ERLVHS**-**FHSYFL**R**PGD**SKK**PIIYDVETLRDGNSFSARRV**A**AIQNGK**P**IF**Y**M**T**ASFQ**A-P**E**A**G**---**FEH**QKT**MP**

P44498_10-122 **HLLKL**EK**ID**DLI**FRG**ESQDL**G**F----RQ**VFGGQVVAQAL**S**AA**M**Q**VAPE**DRI**L**HS**-C**HAYFL**A**PGD**SQY**PIIYDVETLREGRNFSA**L**CV**K**AIQH**K**N**T**I**CH**V**T**ASFQ**V-P**EKG**---**FEH**QNT**MP**

Q87W94_10-122 A**LLTL**EA**IE**E**N**L**FRG**HSQDL**G**F----RQ**LFGGQVLGQSL**S**AA**S**QTV**EE**DRHVHS**-**LHGYFL**R**PGD**AGL**PVVYQVDRVRDG**G**SFSTRRV**T**AIQKGK**P**IF**T**C**S**ASFQ**Y-D**EEG**---**FEH**QTV**MP**

Q8PBH6_14-126 **SLLSL**ER**LE**D**N**L**FRG**QSRDI**G**T----KY**VFGGQVLGQAL**S**AA**QA**TV**ENG**R**Q**AHS**-**LHAYFL**R**AGD**IDH**PIVYDVDRTRDG**G**SFSVRRV**T**AIQHGK**V**IF**F**C**A**ASFQ**E-R**EDG**---A**EH**QSA**MP**

Q9A2B6_9-120 **DILDL**EP**IE**V**N**L**FRG**VSPND**G**F----PR**IFGGLVIAQALLAA**Y**KTV**P-**DRVCHS**-**LHAYFI**R**PGD**VTA**PVLYEVERARDG**G**TFTTRRV**A**AIQHGE**Q**IF**N**L**A**ASFQ**T-P**EDG**---**FEH**QSE**MP**

Q8GYW7_134-248 **RILQL**DPS**E**L**N**I**FRG**ITLPD**A**PIF--GK**VFGGQ**F**VGQALAAA**S**KTV**DFL**KVVHS**-**LHSYFL**L**VGD**IDI**PIIYQVHRIRDGNNFATRRV**D**AVQKGN**I**IF**I**L**L**ASFQ**K-E**QQG**---**FEH**QESTM

Consensus/80% phlpL..lp.s.Fps.....s.......haGGblhtQtLhAA.pTV..pbhspS.bHsYFl.sGc...slhapVcphR-GpsFssRpV.AhQpsp.lb.h.hSFp...ppt...bpH...bP

**TesB subfamily domain 2 (66)**

Secondary St. --------EEEEEE---------HHHHHHHHHH—HHHHHH------------------HHHHH—HHHHHHH------------EEEEEE---------EEEEEEE-----EEEEEEE—-EEE---

Q18061_194-309 CSDLKH**Q**A**RVWMRTR**ER**LN**-**T**G**D**KR**LH**R**WLLA**C**MSD**AI**LL**P**AGM**SA**H**FSQGF------EDSV**HASLDHCLYFHN**H-D**FRVDDWFL**LDC**KSS**VSAE**G**IC**F**I**QGKIW**-**RRDG**V**LIASC**H**QEAIVR**GK

Q9BIA5_225-339 ASDKK**PE**L**YFWMRAR**GD**LS**-**N**-**D**ER**FH**R**WLIAY**N**SD**SL**LV**S**TAV**SP**H**YTTGF------CSSM**L**F**SLDHC**V**WFHR**S-E**VKADEWLLF**EC**KS**RI**A**SG**SRA**TI**EGRIW**-**RRDG**V**LIASC**Q**QEALVR**SK

Q19781_211-325 KEDTE**PM**S**MIWIRAR**EN**LG**-**D**-**D**HR**LH**Q**CVAAYL**T**D**LS**ML**T**TAV**RP**H**I**R**NGF------IPSMSF**SLDHCIW**M**HE**N-E**FRIDDWMLY**ETI**SS**K**A**GG**SRAF**I**EGRLW**-**SRDG**R**LI**I**ST**A**QEALVR**AP

O14734_198-311 LQRME**PK**Q**MFW**V**RAR**GY**IG**-**E**G**D**MK**MH**C**CVAAYISD**YA**FL**G**TAL**LP**H**Q**W**-------QHKVHF**MVSLDHSMWFH**AP--**FRADHWMLY**EC**ESP**W**A**GG**SRGLVHGRLW**-**RQDG**V**L**A**VTC**A**QEGVIR**VK

O06135_175-289 KGDRL**AY**N**RVW**V**KA**LGEM**P**-**D**-**D**PV**LH**T**ATL**L**Y**S**SD**TTV**L**D**SVI**TT**H**G**L**SW-----GFDRIF**AAS**AN**HS**V**WFHR**Q--**VNFDDW**V**LY**ST**SSP**V**A**AD**SRGLGSGHFF**-**DRSG**K**LIATV**V**QEGVL**KYF

P41903_224-340 GDESSL**H**K**HH**P**YR**IPKS**IT**P**E**N**D**AR**YN**Y**VAFAYLSD**SY**LL**L**T**IPYF**H**N**L**PLY------CHSFS**VSLDHTIYFHQ**L--P**HVN**N**WI**YLKI**S**N**P**RSHWDKH**LVQGKYF**D**TQSG**RIM**ASV**S**QEG**Y**V**VYG

Q8SRT9_172-288 GTENK**DM**R**QI**R**IK**I**K**QRQE---**E**VIGIAS**LV**TL**ISD**IL**LV**E**TAL**MASN**L**TLF----SKDLSL**LTSL**N**HVIHF**V**N**LEKSIN**D**GY**I**Y**Y**IV**K**CKGIRN**S**K**AICEGQL**I-**HEDG**T**LIC**L**T**G**Q**Q**GVFR**VK

Q8G3R7_185-299 AEHDS**GK**Q**MVWMKAD**GH**V**--**D**VPQV**MH**R**AMLA**L**G**C**D**QV**M**ME**PVL**RRAG**L**SI-----STPGIS**YASIDHSMW**WY**Q**D--**IDINEWHLY**VQ**DTP**I**A**AH**GRGLG**IA**K**V**Y**-A**QNG**D**LVA**AIA**QEA**M**VR**VP

Q7SI70_198-352 PRPQDK**K**T**R**Q**WYRAK**GK**IS**A**E**GGQQA**H**L**TALAYVSD**SY**FI**G**T**I**G**RI**H**K**L**WRF(35)RPEIGM**MVSLDHSIYFHE**PAK**VRADEWML**SEM**ESP**W**A**GD**GRG**V**VT**Q**RIY**-**NKDG**M**LLATC**V**QEGLVR**LE

Q89WS4_183-297 QKIED**GR**I**HVWIRT**AAK**LP**-**D**-**D**PA**LH**M**CALAYASD**FS**LL**D**A**I**M**ARYG**R**-TL----FDKRMMP**ASLDHAMWFHR**P--**FRADEWLLY**AQ**DSP**S**A**RS**GRGLTRGSIF**-**K**P**DG**T**LVASV**A**QEG**S**VR**ER

Q8FYH7_182-296 REKLE**P**VQ**HVW**V**RAR**GL**VP**-**D**-**D**RA**LQ**A**AILAYLSD**MT**LL**D**TSL**HP**H**G**R**-FI----FDRDMQ**VASLDHAMWFHR**P--**CRLDDWLLY**TQ**D**A**P**S**A**SG**ARGFNRG**A**LY**-**TRDG**V**LIASV**A**QEGLIR**VH

Q82CR2_174-289 GEPRE**PR**S**QVWFRTN**GK**LV**-**D**-**D**PL**LH**V**CLA**T**YVSD**MT**LL**D**SVL**LA**H**G**R**GGW----VTGDVV**GASLDHAMWFHR**P--**FRADEWLLY**DQ**ESP**S**A**SG**GRGLGQ**A**RIY**-**TQDG**Q**L**AI**SV**I**QEGVVR**VP

Q8FPK1_183-293 NPHTA**TE**QV**VWLRSK**AE**LP**-**D**-**D**PT**FH**V**CTLAYMSD**MT**LL**P**GAL**AP**H**---------PGAKVQ**MASLDHAMWF**L**R**P--**FRADEWLLY**DQ**RSP**S**A**GS**GRALTHGRLF**-**NQ**Q**G**D**LVA**I**V**N**QEGLTR**TL

P23911_170-284 GHVAE**PH**R**QVWIRAN**GS**VP**-**D**-**D**LRV**H**Q**YLLGYASD**LN**FL**P**VAL**QP**H**G**I**-GF----LEPGIQ**IA**T**IDHSMWFHR**P--**FNLNEWLLY**SV**EST**S**A**SS**ARGFVRGEFY**-**TQDG**V**LVAST**V**QEGVMR**NH

P44498_171-285 GTKLP**AE**Q**Y**S**WFKTN**GET**P**-L-**D**IK**IQ**Q**CLLAYFSD**FHC**I**L**TAL**HP**H**E**K**-GF----LQKGMK**VA**T**IDHSIWFHR**P--**FDLNHWHLH**AI**ESN**N**A**FG**GRGLAQGQIF**-**SQDG**Q**LIATT**Q**QEGLIR**FS

Q87W94_171-285 PKPGE**P**VK**HVWFRAD**GS**L**K-**D**-VQS**LH**R**YMLAYASD**FN**LL**T**TSL**LP**H**G**K**-TV----WQRDMQ**VASLDHSLWFHN**D--**LRTDDWLLY**AM**DSP**W**A**GN**SRGFSRGSIF**-**NRAG**Q**LVASV**S**QEGLIR**HR

Q8PBH6_175-289 PPKRP**PF**Q**QMWLR**L**S**DP**VG**-**D**-**D**VG**LH**Q**ALLAYASD**FQ**LL**G**TST**FP**H**G**I**-SY----YTPNVQ**MASLDHALWFHR**P--**FRTDDWLLY**SL**DSP**T**A**QG**SRGLARGQFF**-**TRDG**V**LVAST**T**QEGLIR**VV

Q9A2B6_169-283 PVKKS**G**TK**QVWMRAK**AP**LG**-**D**-**D**VK**MQ**Q**AALAYASD**MA**F**ME**SAL**RP**H**G**L**-IW----TTPGIQ**AASLDHAMWFHH**P--**FNFNDW**T**LF**AQ**DSP**S**A**SQ**GRGLVRGQMF**-**SQDG**K**LLASV**A**QE**C**LMR**VR

Q8GYW7_304-416 QTKSP**PR**LN**YWFRAK**GR**LS**-**D**-**D**QA**LH**R**CVVAFASD**LI**F**CG**VGL**NP**H**R**R**-------KGVKSA**A**L**SLDHAMWFHR**P--**LRADEWLLY**VIV**SP**T**A**HET**RGFVTGQMF**-**NR**K**G**E**LVVS**LT**QEALLR**EA

Active Site -----------------------------------*----------------------------*-----------------------------------------------------*------

Consensus/80% .....sb.bhWb+sp..ls.p.-..bp.hhhtahSD..bl.ssh..H.b.............hsSlDHsbaFHp...hphscWbLa..p*s.A..tRtbspGpba.ppsG.Lls*s.QEtlhR..

**4HBT class II subfamily (59)**

Secondary St. –-EEEEEE--------EEEEEE-----EEEEEEE—-HHHHHHHHHHHHHHHHHH-------EEEEEEEE-------------EEEEEEEEEEE---EEEEEEEEE--------EEEEEEE----------

CMA2_BACSU_1-125 MDM**KH**TL**L**EAL(10)**RC**V**A**V**MPVD**H**RTVQPFGYLHGGASVALAETAA**RP**GA**QN**LI**--DH**T**T**QACVGLEINANHLKSVKEG**--**TV**K**A**I**AE**P**VH**I**GRTTIV**Y**HIHI**Y**DEQ**---E**RLICISRCTL**----A**VI**K

Q04416_34-148 **VG**FV**I**DE**M**TP**E**----**RA**T**AS**VE**VT**D**TL**R**Q**R**WGLVHGGA**Y**CALAE**M**LA**TE**A**TV**AVV**--HE**K**G**MMAVG**Q**S**NH**TS**FF**R**P**VKEG**--**HV**R**A**E**A**VR**IH**A**G**S**TTW**F**WDVSL**R**DD**A---**GRLCAVSSMSI**----A**V**RP

O85402_25-139 **LGIEF**TE**V**GK**N**----**YL**R**GRMPVD**H**RTHQPIGL**M**HGGASCVLAETLGSVAA**N**FCV**--DS**N**E**LYCVGLDINTNHVRSARSG**--**FV**I**G**T**AK**PF**H**I**GKSTQVW**G**IEI**F**DEK**---**DRLVSVSRLTM**----A**VL**K

Q8KCU2_30-146 **LGIEI**TA**V**GP**D**----S**M**T**ATMPVD**H**RTIQ**R**IGILHGGASLALAETVGSIAA**S**YCV**--DR**E**K**QF**I**VG**Q**EINANHLRAVRQG**ESS**V**H**A**T**AT**P**LH**L**GRTSQVWDIKI**R**DDK**---**GRLVCVSRFTA**----A**VL**E

YG18_PSEAE_25-139 **LGIRF**EA**F**DD**E**----S**L**T**ASMPVD**S**RTHQPFGLLHGGASVVLAESLGSMAS**Y**LCV**--DT**S**Q**YYCVGLEVNANHLRG**L**RSG**--R**V**T**A**V**AR**A**IH**L**GRTTHVWDIRL**S**GDD**---**GK**PS**CIARLTM**----A**VV**P

Q8EHY6_30-144 **LGIEI**SE**I**GD**D**----**YM**K**ATMP**A**T**PA**VH**N**PLGIVHGGANVALAETVASYAA**N**FAV**--DF**E**Q**YYCVG**Q**EINANHLRASRNG**--**VL**T**A**T**AK**P**IH**V**GKRS**S**VWEI**L**I**H**NS**A---**GELTCISRMTA**----A**VV**K

Y788_PASMU_24-137 **L**A**IQF**SAQGE**N**----**WL**E**ATMPVD**Q**RTIQPMGFLHGG**L**SVALAETIGSMAG**F**CCI**---T**E**N**QFVLGLEINANHLR**P**VKQG**--**IV**T**A**R**AT**P**IH**L**G**T**RTQVWQIEI**K**DQQ**---**DQLCCLSRLTL**----S**V**GN

Q82W63_29-144 **IGIRF**LE**V**GP**D**----**FL**K**ASMPVD**H**RTTQPFGILHGGASCVL**S**ETLGSVSA**W**MTI**--DP**E**Q**Y**R**AVGIEINANHIRAVTQG**--N**V**I**G**VC**T**P**LH**V**GRRTQVWQ**T**DI**TE**EE**--T**GKRIAVSRLTV**----A**II**E

Q9FAE9_12-126 **LGIEI**VENTA**E**----**RC**V**A**V**MPVD**H**RTVQPFGYLHGGASVALAETAASAGA**QN**LI**--DH**S**T**QACVGLEINANHLKSVKEG**--**TV**K**A**I**AE**P**VH**I**GRTTIV**Y**HIHI**Y**DEQ**---E**RLICISRCTL**----A**VI**K

YBDB_ECOLI_24-137 **LGI**V**Y**TR**L**GD**D**----V**L**E**AEMPVD**T**RTHQPFGLLHGGASAALAETLGSMAG**F**MMT**---R**D**G**QCVVGTELNATH**H**R**P**VSEG**--K**V**R**G**VC**Q**P**LH**L**GRQNQ**S**WEI**V**V**F**DEQ**---**GRRCCTCRLGT**----A**VL**G

Q7VPM0_25-139 **LGIEF**IA**I**GD**N**----**WL**E**AQLTVN**E**KTMQPFG**V**LHGG**I**SAALAETTA**N**AGS**L**LTC**---EAH**QMAVGMELN**I**SHLKSV**PY**G**Q-**T**AI**A**R**A**YP**VK**I**GRE**I**QVWQVDI**K**DES**---**GHLCAVARLST**----K**IL**N

Q7MU91_290-405 **LGIR**CTK**I**ARG----**YV**E**ATMPVD**I**RT**R**QPMGILHGGASLA**F**AETLAGFGS**V**ALC**---NPG**EI**Q**VGLQVSGNHVSSA**L**EG**D-**VL**R**G**E**AS**IM**H**Q**GRSTHVWSINI**Y**STK**--S**GKLICTCRV**LN----S**IL**K

Q8CT87_8-122 FEM**KI**EQKEDG----**KV**VV**SMPVT**D**KV**K**QPFGYLHGGASLAL**G**ETA**C**SIGA**A**HLI**--DT**Q**Q**FIPLGLEMNANHIRSTRQG**--**HV**R**A**F**AS**L**IH**Q**GKTTQVWNIDI**K**DDH**---**DQLISV**M**RGTI**----A**I**KP

Q9S2J8_54-164 M**GVQI**VE**A**SA**D**----**RV**V**GTMPVE**G**N**-**TQPYGLLHGGASAVLAETLGSVGS**M**LHG**---GAA**KIAVGVDLNCTH**H**RGVRSG**--**LV**T**G**V**AT**P**VH**R**GRST**ATY**EV**V**I**S**DEQ**---**DRRVCTARLTC**------**L**L

YO06_DEIRA_43-156 **LGIRY**VS**M**AR**E**----**RV**V**ATMPVE**G**N**-R**QP**A**GRLHGGATLALAE**E**LASVGS**W**L**N**L**--DP**Q**R**QVAVGVDLNGTHVRGVSEG**--**HV**T**A**E**AR**LSYR**GRS**L**MVWEIE**MK**DEK**---**GR**T**TSLCRCTC**----N**VI**S

YI47_MYCTU_21-137 **LGLQF**TE**L**GP**D**----G**A**R**AQLDVR**P**KLLQ**LT**G**V**VHGGV**Y**CA**MI**ESIASMAA**F**AWL**NSHG**E**GGS**VVGVN**N**NTD**F**VRS**I**SSG**--**MV**Y**G**T**AE**P**LH**R**GRR**Q**Q**L**W**L**VTI**T**DDT**---**DR**V**VA**R**GQV**R**L**----QN**L**E

Q8FTI0_68-179 **LGLRY**VE**V**GP**E**----**RV**V**SEL**R**VT**A**NHLQP**A**GLV**N**GGV**Y**CA**I**AESTGSTAG**I**IFG**----**R**G**K**P**VVGVN**N**NTD**F**I**A**SVRDG**--**VI**R**A**E**AT**A**I**QK**G**G**RTQVWQI**LCTH**N**----**GELVA**R**TTL**R**T**----M**VL**G

Q9SX65_18-154 **LG**F**EF**DE**L**SP**T**----**RI**T**GRLPVS**PV**CCQPF**KV**LHGGVSA**LI**AESLASMGA**H**MA**S----GF**K**R**V**A**GIQLS**I**NHLKSAD**L**G**D-**LV**F**A**E**AT**P**V**ST**GKT**I**QVWEVKL**WK**TT**(6)KI**LIS**S**SRVTL**(17)KM**V**A

Q9CHK5_7-132 **L**N**IT**DFQ**V**FT**D**(11)**KF**S**SKM**IL**S**DF**HAQPHGFL**N**GGASLALAE**I**TAGMAS**N**AIG**---S**S**Q**YFALG**Q**SISANHLNS**K**KC**EG-**FV**N**A**CGLL**LK**N**GKRNHVWEIKI**T**DEN**---E**TLIS**QI**TVV**N----A**LV**P

Q89MN1_54-169 K**GVEF**VE**A**EK**D**----**RV**V**ARMTVR**P**DLC**TL**H**HT**IHGGAVMALA**D**SVGAAA**TV**I**N**L**--PE**D**A**KGT**TT**LE**SK**TN**F**I**G**GAKEG**T-**TL**I**A**T**AT**P**VH**R**GRRTQVWT**T**RL**E**TED**---**GKLVAVVT**Q**T**Q----L**VL**V

Q9RW22_2-116 **VGIRF**TH**I**ERG----**LL**R**SELTVR**P**ELF**A**P**N**GYLH**AA**SVVALA**D**TT**C**GYG**TR**VLL**--PD**E**AT**G**FTT**IEL**K**SNHL**GT**SRQG**--**VV**TCE**AR**A**VH**A**GRTTQVWD**A**EV**R**NEQ**---**GN**V**MAL**F**RCT**Q----A**VL**Y

Active Site -------------------------------------------*--------------------------------------------------------------------------------------

Consensus/80% lGlpb..h..p....bh.tpbsVp.phhQPbGblHGGsshsLAE*htthtt.hhh....p.bhslGhphsssHlptsppG..hl.t.Ap.l+.G+psbVWplpl.spp...spbhshsphsh.....ll.

**CBS associated subfamily (21)**

Secondary St. –EEEEEEE------HHHHHHHHHHHHHHHHHHHHHH---EEEEEEHHHHHH-------EEEEEEEEEE------EEEEEEE----EE-HHHHHH---

Q9K832_338-434 **YEVE**I**TPQMTNQLGTIS**H**GVMTSLV**I**E**S**G**S**R**V**L**RKY**KK**G**DLVVENITLYFLKPVQIDS**R**LTIRPRVLEIGRK**H**GKIDVE**M**Y**HE**GEIVGKA**LF**MAQI**I

O34921_340-436 **YEYEVTPQMTNQLGTIS**Y**GVFTTILT**Q**AA**N**R**F**L**RSK**KR**GE**LVIESITIFFLKPVQ**M**ES**V**IEVKPRILEAGRK**F**GK**ME**VEVH**SQ**GHIVSKA**M**LMVQL**M

Q81KX8_339-435 **YQFSVTPQMTNSIGTLS**Y**GVFATIVTEAT**N**R**V**I**RAQ**KK**S**DLIVENLTIYFVKPVQIDN**V**VSVHPKVLEIGRK**F**GKVDVEVH**HE**GNVVGKA**L**LMVQL**I

Q817E4_339-434 **YQFSVTPQMTNSIGTLS**Y**GVFATIVTEAT**N**R**V**I**RAQ**KK**S**DLIVENLTIYFVKPVQIDN**V**VSVHPKVLEIGRK**F**GKVDVEVH**HE**GNVV**W**K**S-**IT**YGSV

Q8EPC7_337-433 **FTTR**II**PQMTNQLGTLS**N**GVFTSLI**A**EAC**S**R**M**L**LKV**KK**A**DI**S**IENITVYF**S**KPVQIES**E**LTIKPKIIDVGR**LY**AKIDVEVY**NI**DQ**M**VGK**GL**IMAQL**I

Q835K5_340-436 **FRFSV**A**PQMVNSVGTIS**F**GVLSEIIS**N**VT**Q**R**TMLMN**QK**R**NVLIEQ**VN**LH**Y**LR**L**IQ**L**ES**E**LDIRPRILEIGRR**S**AKLDIEVY**LE**N**V**IVAKA**I**VVCQV**M

Q99TF8_333-429 **ITVEVSPL**L**INHYGTVS**KAA**FVSIIEET**IQYEMRKF**KK**G**NV**M**IENL**N**I**VY**IKTV**P**IES**H**ITVR**FG**ILDVGR**NF**AKI**E**V**NM**H**SQ**ND**K**VA**S**A**L**VICQ**MF

Q8P0G9_331-427 **YQV**V**VEP**T**MIDSAGN**M**S**N**GVISE**F**LKEIS**I**R**A**L**TKK**HQ**K**NIIIEQMMVYFLHAIQIED**E**LKI**Y**PKII**TE**NRR**S**STIDIEIF**VD**DQV**I**AKA**I**ITTKI**N

Q99Z80_331-427 **YQV**V**VEP**T**MIDSAGN**M**S**N**GVISE**F**LKEIS**I**R**A**L**TKK**HQ**K**NIIIEQMMVYFLHAIQIED**E**LKI**Y**PKII**TE**NRR**S**STIDIEIF**VD**DQV**I**AKA**I**ITTKI**N

Q97QW4_330-425 VV**ITVEPFML**E**K**N**GVL**AN**GVLAEILTHMT**-**Q**D**L**VVN**S**GR**NLIIEQMLIYFL**Q**AVQIDD**I**LRIQARIIHHTRR**S**A**I**ID**Y**DIY**HGH**QIVSKA**N**VTVKI**N

Q8DPV2_330-425 VV**ITVEPFML**E**K**N**GVL**AN**GVLAEILTHMT**-**Q**D**L**VVN**S**GR**NLIIEQMLIYFL**Q**AVQIDD**I**LRIQARIIHHTRR**S**A**I**ID**Y**DIY**HGH**QIVSKA**N**VTVKI**N

Consensus/80% bphpVpPbMhsphGslS.GVbspllpchs.p.l...pp.slllEpbhlaFl+slQI-s.lplps+llchsR+.tplDl-la..splVtKA.lhspl.

**PaaI subfamily (19)**

Secondary St. HHHHHHH---EEEE-----EEEEEE-HHHH--------HHHHHHHHHHHHHHHH---HH---EEEEEE—EEEEE-------EEEEEEEEEE-----EEEEEEEE-----EEEEEEE—-EE---

PAAI_ECOLI_15-132 **ND**A**C**A**KALGI**D**I**I**SMD**E**GFA**V**VTM**T**VT**AQ**MLNGH**QS**CHGG**Q**LFSLAD**T**AFAYAC**---**NS**Q**G**LA**AVA**SA**CTIDFL**R**PGF**A**G**DT**LTATAQ**VR**H**Q**G**K**QTGVYDIEI**V**N**-**Q**QQ**K**T**VA**L**FRGK**S**HRI**-

YM64_ARCFU_29-146 **ND**K**L**F**ELL**D**A**R**I**L**EM**KE**GYAKVEM**V**VK**K**EHLNA**AN**VCHGGIIFSLAD**L**AFALAS**---**NS**H**G**KL**ALAI**E**VSITYM**K**AAY**E**G**EK**LVAEAK**EV**N**L**G**N**KTATY**L**MEV**K**N**-**S**A**NKLIA**L**AKGT**V**YRV**-

Q8A2G2_9-126 **KD**L**F**A**E**NA**GV**V**L**L**EV**RE**GYSKAKL**E**IK**P**EHLNA**GAR**T**Q**GGAIFTLAD**L**ALAAAA**---**NS**H**G**TL**A**F**SL**SS**SITFL**R**AS**GP**G**DT**LYAEAR**ERYI**G**R**STGCYQIDI**T**N**-**Q**N**GDLIA**T**FESS**V**FR**K-

Q9F9V0_29-146 **ND**R**V**L**HA**N**GI**R**F**EA**IG**P**GYAKVTM**T**VR**E**DMLNGF**D**ICHGGFI**TL**LAD**T**AFAYAC**---**NS**G**N**EQ**TVA**SG**ISLDFM**A**PGR**P**G**EV**LCAEAK**EVFA**A**G**RTGVYDISV**T**N**-PK**GELIA**V**MRGK**S**YRL**-

Q8TSR0_21-137 **KD**K**F**AA**H**A**GI**E**L**L**EAA**P**GYAKATL**E**IE**E**KHLNAL**R**AV**Q**GGAIFTLAD**L**AFAAAS**---**NA**Y**G**IA**AVGI**NS**NISF**VK**AA**TK**G**-T**LTAEAK**ET**S**INP**KIATYTVNV**T**D**-**D**A**GDLVA**I**FQG**MV**YR**K-

Q8R8Y9_22-139 **DT**N**F**H**QLIGV**H**V**V**ELG**Q**GYA**V**TEI**E**IE**E**KHLN**P**L**N**IAHGGVLFSVMD**IT**MGMAA**---R**T**V**G**KQ**VITI**E**MNINYL**S**P**V**R**V**G**EK**V**K**AK**G**K**IV**H**A**G**S**KT**T**VA**V**CE**AY**A**-**E**D**GRLLA**V**AR**E**T**F**F**N**V**-

O29336_51-171 **SA**P**W**Y**KLIGM**VPKLQ**G**DR**V**VVEM**E**ID**R**S**KHL**Q**AL**G**TTHGGAI**A**SVLD**S**AIGLNV**(4)**V**KM**G**KT**AVTA**Q**LNI**H**YI**R**P**VT-EGK**IV**GVG**M**PM**H**I**G**S**KV**T**VG**Y**GEV**R**N**-**E**E**GELVA**A**GTAT**F**YII**-

Q7WGY3_28-145 G**D**A**A**S**QGLGM**T**V**V**EIA**P**GYAK**L**SM**P**VR**A**DMLNGH**K**TCHGGFIFALAD**S**AFAFSC**---**NS**R**N**VS**TVA**SG**CTIDYL**A**PGL**E**G**DV**LTA**V**AQ**ER**S**L**A**G**RTGIYDVTV**T**N**-**Q**Q**GR**S**VA**V**FRGR**S**YRI**-

Q845J9_20-137 **RD**Q**A**S**R**R**MGM**R**L**LA**AG**P**G**S**AQV**G**M**S**VR**E**DMI**Q**GH**G**TCHGGYLFALAD**S**AFAFAC**---**NS**Y**N**EA**TVAI**G**CSIDY**VA**PAR**L**G**DT**LTAQAI**EQ**S**R**S**G**RTG**N**YDVRI**E**N**-**Q**HRQ**LIA**L**FHGK**S**YKV**-

Q93JC6_20-137 A**D**E**A**S**RGLGI**E**L**V**EHG**E**GTALVRM**T**VT**PA**MVNGH**R**IAHGGFLF**L**LAD**T**AFACAC**---**NS**H**G**PV**TVAA**G**ADIVF**VA**PAR**E**G**DV**LVARAE**ERVRYG**R**S**GIYDVSV**-R-**R**G**DEVVA**E**FRGR**S**R**S**V**R

Q8FRU2_30-146 **ND**A**A**S**KMLGV**V**I**T**ELS**PEQ**ARGHF**T**IR**E**DM**C**NGH**G**TA**Q**GGILFT**F**AD**AV**FAGVC**---**NA**A**G**DV**AVAA**Q**VGI**H**YL**S**PAR**V**G**EV**V**E**AEA**VCR**Q**NWG**R**N**GITDVTL**-R-VG**DRIVA**E**FRGT**S**R**V**V**-

Q97VK2_3-119 **ES**P**F**L**KFL**N**I**E**L**E**EI**RE**GYARVS**GV**V**AK**DFLN**L**H**N**TAHG**S**FIFAIAD**A**AF**E**Y**I**S**----**N**F**S**RD**SVAL**H**MDIDFR**R**P**V**K**E**G**EK**VIAEAF**EE**S**S**G**K**TTSLYRI**I**V**K**N**-**E**D**GKLVA**Y**VTA**LV**YHL**-

Q7VZJ5_12-129 **T**IP**F**M**QLLGV**VPE**H**S**G**N**GTARTRL**PA**R**A**DLVNSR**GDI**HGGTLMSVLD**FT**LGAA**I-RG**DT**PEVG**V**A**TI**D**MN**T**SFM**S**PGR**--GD**LV**I**E**T**R**CL**R**R**G**A**SIAFCKGEI**R**D**-**S**A**GELVA**K**ATAT**F**KII**-

Q8XYJ4_11-129 **N**IP**F**L**QWLGV**R**C**L**KVA**H**G**E**GIVEL**P**LE**A**RH**M**NSW**E**MAHGGV**T**MTLLD**V**SMAMAG**RSA**DT**H**G**RG**VVTI**E**M**KT**AFM**Q**PGR**--GT**L**R**AHAR**CV**H**Q**S**T**TMAFCEGEV**R**D**-AD**GKLVA**R**GSGT**F**KFV**-

YN21_DEIRA_25-142 AMS**Y**A**EVLGM**T**I**L**DAS**PD**L**T**RV**A**L**T**VT**EAG**LN**M**H**G**TAHGGLIFSLAD**E**AFAV**I**S**---**N**LD**A**-Q**AVAA**E**T**HM**SFF**R**AAR**E**G**ER**LVA**V**A**TPE**R**V**G**R**TLATYRIEV**RRG**E**E**GEVLA**L**F**L**GT**VS**R**R-

Proposed active site ----------------------------------------------*----------------------------------------------------------------------------

Consensus/80% ps.h.phbGh.h.phs.Ghtbspb.lp.cblNtb.hsHGGhlbslhD.tbthss...ss.s..slsh.hslsab.stb.G..lhApAb..p.t.phthhphpl.s.p.scllA.hptp.bbl.

**Hydroxyacyl-CoA dehydrogenase associated subfamily (14)**

Secondary St. ------------E-----HHHHHHHHHHHHHHHHHHHH----HHHHH----EEEEHHHHHHHHHHHH----EEEEEEEE---HHHHHHHHHHH--------EEHHHHHHHH-------------HHHHHHHHHHHHHH----

Q7WNE6_4-142 **L**PS**TT**L**SVD**PA**WID**AY**GHMN**A**AEY**VG**VFD**R**VG**FD**LL**REV**GVG**L**D**YT**E**ATQ**C**G**IYTM**NIQ**V**A**YLREV**LA**G**DP**L**M**LRVRLL**E**ADDKRV**L**CLMELW**---QTRDDY**LAAT**M**E**Q**L**S**LHVD**L**RT**R**RS**K**PFD**PA**L**AER**L**ART**VS**E**H**AQ**A**

Q89UR6_4-142 **E**AT**YR**G**TV**YPWQC**DH**V**GHMN**I**MWY**VGK**FD**E**AN**WN**LF**AR**LGLT**P**S**YL**R**STGRG**M**AA**V**QQN**ITY**K**REL**LA**G**DI**V**E**IR**S**HLL**E**V**R**DK**S**IRFRHDM**T---NAETGE**IAA**F**CE**ITGV**HMD**R**EL**R**RSAPFT**DA**I**RDAALR**HL**AEPAE**A**

Q92NF5_345-482 **L**RL**VD**T**KVN**AA**WVDYNGHMT**E**HRY**LQ**LFG**D**TSD**A**LL**KV**IGVD**FAYV**E**A-G**HSYYTVE**T**HIRHL**G**EA**KL**G**QA**L**YT**TLQLL**SS**DEKRIHFF**T**RIH**---DAASGD**VIATAE**Q**M**M**LHVD**A**KA**G**KSVP**A**P**AE**V**MAK**L**KP**IAE**G**H**AK**L**

Q8UJY0_333-468 **L**RL**HE**A**HVN**GG**WVDYNGHMT**E**FRY**LQ**V**L**G**D**ATD**A**LL**IH**IGLD**A**D**YRAA-G**HSAYTVE**T**HIRHL**A**EV**KA**G**AR**L**T**VETRLL**G**YDDKRLRLHH**A**IL**---NEDGE-**TVATGE**H**M**L**LHVD**T**KA**N**RTVA**M**P**PA**L**MRA**L**DH**L**-**N**A**Q**EE**G**

Q93RX6_4-145 **L**PL**LH**S**TVR**PE**WIDYNGHLS**E**AFY**VL**VFG**H**ATD**A**LM**TGT**GLD**SGYR**E**STR**CSLYTVE**S**HIRFLR**D**V**SE**G**AH**L**A**VRTRVL**G**A**AARKA**RFQHEMY**(8)PAPDAA**AVATTE**L**L**AV**HVD**Q**QA**G**RAT**E**FP**ES**V**RRRFTE**LTE**-----

Q9HTH9_5-143 **L**TT**YE**TP**V**LPE**WVDYNGHLR**D**AFY**LL**VFS**Y**ATD**A**LM**AH**IGLD**S**Q**NR**D**ASG**HSLFTLE**C**HLNFLHEV**KE**G**AR**V**E**VRTQLL**G**HDRKRLHIHH**A**LY**---LPGSGQ**ALA**L**SE**Q**M**L**LHV**SL**DG**P**RSAPF**EGE**V**LAR**V**EA**LAE**A**H**RA**L**

Q88R33_4-142 **L**IT**YR**T**TVQ**ED**WVDYNGHLR**D**AFY**LL**IFS**Y**ATD**A**LM**DR**IGLD**A**D**SRGQSGN**SLFTLE**A**HINYLHEV**KL**G**TE**V**W**VQTQIL**G**FDRKRLHVYHSLH**---RAGFDE**VLAASE**Q**M**L**LHVD**LA**G**PQ**SAPFG**HT**T**VCR**L**NH**LVE**Q**Q**EG**A**

Q8CUV9_4-141 **M**FN**FK**S**EVK**PE**WVDYNGHMN**D**A**A**Y**AA**VFS**Y**AVD**S**LM**EF**FGLT**K**E**VI**E**NEK**YTLFTLE**T**HLCYLNEA**YL**G**EE**L**H**VDLQLL**D**VDSKRLHAFFTMK**---NAKEV-**VIATSE**Q**M**L**MGMD**Q**EI**G**KPAPF**LPH**V**KEN**I**DHV**WN**N**H**QW**I**

Q8CTX3_5-142 **Q**YV**FH**T**KVH**RD**WVDHNGHLN**D**AMY**NR**IFS**D**TTD**D**WL**GH**LGLT**I**N**AI**Q**SYQ**YTVFTLE**N**HV**M**FLNEM**KENED**V**I**VKVHLH**D**YDSKRLHVLMEMF**---NADDD-**LCAT**Y**E**V**M**L**MGID**T**T**SG**RPSAFP**ND**I**LNN**I**EH**YYN**I**E**NVE

Q99RS5_5-142 **L**FT**HT**Q**TVT**SE**FIDHN**N**HMH**D**A**N**Y**NI**IFS**D**VVN**R**F**NYS**HGLS**L**K**ER**E**NLA**YTLFTLE**E**H**T**TYLSEL**SL**G**DVFT**VTLYIY**D**YD**Y**KRLHLFLTL**T---KEDGT-**LA**S**TNE**V**M**M**MGI**NQ**HT**R**RSDAFP**ES**F**STQ**I**AH**YYK**N**Q**PT**I**

Q8CRB5_4-141 TFT**VT**R**TVT**EDA**ID**N**N**N**HMH**D**AYY**NI**IFS**E**V**I**N**K**F**NEV**HGLS**W**S**ER**D**RLQ**YTVFTVE**T**H**T**TFLHEL**TL**G**QEFN**IELFLY**N**YDDKR**T**HFFLRML**---IDNQE-**VVATNE**V**M**M**LGID**R**T**QR**RAAPFP**KH**Y**LNA**I**QD**YAH**K**Q**EK**I**

Q98KG3_7-144 **F**VSKPM**D**I**E**KD**WIDYNGHLN**M**AYY**NV**LFD**R**CSD**EA**F**EA**MG**M**G**L**D**YV**K**QRR**LTIYTAE**V**HVCY**V**QEL**HLDHK**V**Q**VSFQLI**D**HDEKRLRAYQEIR**---HIDGW-**LAATSE**T**L**S**LHVD**M**SG**P**KVAPFP**AD**V**MAK**V**EA**M**RAA**H**SV**L**

Q89FY9_48-186 **F**SASIM**Q**I**E**PQ**WIDYNGHLN**M**AYY**NVM**FD**R**A**I**D**E**FW**LE**LGIG**P**T**YK**K**ERH**GSTFTAE**C**HVRYLREI**HL**G**DP**V**R**I**L**VWLL**E**ADDKRLHTFEEMR**---HATEGW**L**S**ATSE**N**M**S**LHMD**M**NA**R**RVVAFP**PD**I**QQR**I**AG**IAR**A**H**ST**L**

Consensus/80% b..hp.pVp..alDaNGHbp.hbY..lFs.sss.bb..bGls.p..p...h*haThE.HlpaLpEh..G..l.lphblb.hDpKRl+hbbpbb.........hhAssE.b.bhhD.ph.+sssFs..h...l..bhp.p..h

**Acetyltransferase subfamily (13)**

Secondary St. –HHHHHH-----HHH—EEEEE------EEEE--------------HHHHHHHHHHHHHHHHHHHHHHH-----EEE-----EEE-------EEEEE---------HHHHHHH----EEEEEEEE-----EEEEE---EEEEE

P32148_176-314 **QLQQ**A**W**Y**E**H**IPLSEKMGV**R**I**Q**QYTGQKF**I**T**T**MPETGNQN**P**H**H**TLFAGSLFSLATLTGWGLIWLMLRE**RH**L**G**GTIIL**A**DAHIRY**S**KPIS**G**K**P**HA**V**A**D**L**---GA**LSG**D**LD**R**L**A**R**GR**KARV**QM**QV**E**I**F**GD**ETP**GA**V**FEGTYIVL**P

Q7MQ05_159-297 **ELQD**R**W**E**K**Q**IPI**A**DKMGI**K**I**N**QYTGYQF**E**C**C**A**Q**LNPNLN**P**H**N**TLFAGS**A**FTLATLTGWGM**A**WLLLKE**RG**L**K**GDIVL**A**DSNIRYRHPVD**K**T**P**IA**S**T**S**L**---DG**ISG**D**LD**R**L**A**S**GR**KARI**V**I**L**V**T**I**Y**SG**DV**AAV**E**FTGTYM**L**L**P

Q7N9S1_160-298 **ELQQ**A**W**Y**K**H**IPLSEKMGV**R**I**S**QYTGQSF**I**T**T**MPEAGNQN**P**H**Q**TIFAGSLFSLATLTGWGLIWLLLQE**RQ**L**G**GDIIL**A**DADIRYKKPV**IG**R**P**KS**V**A**D**L**---KN**MSG**D**L**AR**L**A**Q**GS**KARV**K**LDV**I**V**S**GD**QG**IGA**V**FTGTYMVL**P

Q7QM69_158-296 **DLTT**R**W**S**R**G**IPISEKMGV**H**I**T**HYDGQTF**H**L**K**ANLAANLN**V**H**D**TMFAGSIYSQCVL**A**GWGLIWLQLKE**AG**L**V**GD**T**VL**A**EGNIKYYRPVK**E**E**P**EA**R**V**A**R**---EG**MPA**V**LE**P**L**KAGES**AKF**S**LK**IK**L**F**SG**DK**LAA**E**F**L**GHYVV**QP

Q8E989_8-143 -**LRQ**T**W**H**S**T**IPVSEFM**Q**I**APL**SFTD**G**EL**S**V**S**APLAPNIN**L**H**H**TMFAGSIYTI**M**TLTGWGMVWLQ**Q**Q**LLN**V**D**GDIVL**A**DAHIRYL**A**PVT**SAP**E**VK**V**R**W**-----P**D**TN**LS**P**L**Q**R**GR**KAKV**K**LEV**Q**L**F**CD**GK**L**C**A**Q**FDG**L**YVSV**P

Q8KF72_4-145 **KLQE**I**L**D**S**A**IPLTQ**A**MGI**V**V**E**RYTGREL**T**I**I**APLANNFN**HLG**TAFGGSLY**IA**CVLSAWGLLYLRLRE**AG**I**K**GSIVI**R**KGN**A**EYLRPVT**G**D**IV**A**T**G**T**L**PTEEE**FAA**L**IE**SFD**R**KG**KAKM**T**ICA**V**I**E**V**EGK**VAV**K**FEGE**F**AVV**R

Q8YKV8_5-146 **Q**V**EQ**Y**L**H**Q**N**IPISQQMAV**S**V**V**S**I**D**E**K**GVI**L**A**APL**L**PNIN**H**H**G**TVFGGSI**S**NLA**I**LSAW**T**LVY**V**RLQE**LF**I**NSR**IVI**R**R**N**TVDYFQPLQ**G**D**F**QA**H**C**IAPVEDS**WSS**F**VT**N**L**D**E**KG**K**G**RI**I**LDA**E**I**T**SS**GI**CAA**K**FQGEYVAL**I

Consensus/80% pLpp.b.p.IPl*pbMtl.l.passbpb.h.hsbssNbN.H.ThFtGSlasbssL*tWGblaLbLpE..l.GsIll.ctplcYbpPlp.p.bt.s.b.....bst.lp.L.p..KA+h.lps.l.ss..hts.FpGpYhsl.

**FapR subfamily (11)**

Secondary St. -----EEEEEEEEE---HHHHHH----EEEEE----EEEE-------EEEEEEEE-------EEEEEEEEE—EEEE-----HHH

FAPR_BACHD_98-179 **F**S**RT**G**IARGHYLFAQANSLAVAIID**DD**LALTA**K**ATIRF**T**R**Q**VK**A**GERVVAKAEVQK**V**E**R**D**--**RTLV**V**VN**S**FV**E**QELVFSGDFLM**

FAPR_CLOTS_100-181 **F**L**KT**K**I**I**RGHYIYSQAESLAISVID**AE**AALIG**V**ANIKYKY**P**V**R**IGDRLVAKAEVIR**K**RGN**--**KYFVWV**MI**KV**K**NKEVFRGKFIL**

FAPR_THETN_100-181 **F**V**KT**K**IV**K**G**Q**YIYSQAESLALSLID**AP**AALIG**V**ANIKYKY**P**VKVGDRLVAKAEVIR**Q**RGN**--**KYFVWVK**I**KV**K**EKEVFRGKFIL**

FAPR_BACAA_101-184 **F**K**RN**Q**IARGHHLFAQANSLAVAVID**EE**LALTA**K**STIRYIR**P**VKLGERVVAKARVED**V**END**KG**RTVVKVR**S**FV**G**EELVFTGTFEM**

FAPR_BACCR_101-184 **F**K**RN**Q**IARGHHLFAQANSLAVAVID**EE**LALTA**K**STIRYIR**P**VKLGERVVAKARVED**V**END**KG**RTVVKVR**S**FV**G**EELVFTGTFEM**

FAPR_STAAM_98-179 **F**H**KT**G**IARGH**V**LFAQANSL**C**VALI**KQP**T**V**LT**HE**SSI**Q**FIE**K**VKL**N**D**T**V**R**A**E**ARV**VNQT**A**K--**HYYVEVK**S**YV**K**H**T**LVFKGNFKM**

Consensus/80% F.+s.IsRGHalatQApSLAltlID..hALht.tsI+abb.VKlG-RlVAKAcVbc.css..+hhVbVp.bV.pcbVFpGpFbb

**MSCP subfamily (11)**

Secondary St. -----EEEEEEE—-HHHHHHHH------HHHHHHHHHHHHHHHHHHHHHHH-----EEHHHHHHH-----------EEEEEE------EEEEEEEEE-------EEEEEEHHH—-E----HHHHHHHH-----

Q7QH28_39-166 **VTDKHTI**Y**GFCTTQDVDFLL**N**HMNNGRYLRELDF**C**RF**Y**WY**G**RT**RF**W**P--**L**GNAKNI**LQG**E**C**M**VRYRR**M**L**PI**FK**A**YKVET**Q**LVWWDDRSIYFEHKFIT**L**HD**---**GFIRTVAYSRQRAI**G**VNLL**EY**VQQF**PE**C**RQ

Q7QH30_59-188 **VTDQT**A**I**Y**GLCTTQDVDIFI**R**HMNNARYLRELDFARF**H**FY**G**LTGIY**GK**IK**A**K**R**G**G**AVQGASSVRYRR**T**I**PI**FT**A**YKITTKLVWWDEKAIYLEQQFVT**LA**D**---**GFVRAVAMSKQCIT**N**VDVL**E**LMKEF**PGAEQ

Q9W440_38-167 **VTDTTTI**Y**GLCTSQDVDIFI**R**HMNNARYLRELDFARF**H**FY**A**LTGLY**ER**IR**D**R**R**G**G**AVQGASSVRYRR**T**I**PI**FH**P**YKIQTKLIWWDDKAIYLEQQFIT**L**SD**---**GFVRAVAMSKQNIT**N**CNVL**E**VLKTY**PE**T**AQ

Q7QJ53_44-173 **ILDT**G**TV**M**GLCLTNDIDTLL**Y**HMNNARYLREIDFARV**D**FY**E**RTSLY**RT**IR**S**K**G**G**S**VVQGATTIRYRR**F**I**RP**FT**R**FTISSRIVYWD**N**QSIFMEHRFLG**G**TD**---**GFIHCIALCRQRVM**Q**CSVE**D**VM**A**TL**LK**S**GI

Q9W439_46-175 **ILDTTTV**N**GLCLTNDVDTLL**Y**HMNNARYFRELDFARV**D**FY**E**RTNLY**RT**I**TG**M**G**G**S**V**F**QGAATIRYRR**F**I**RP**FH**R**FNI**I**SRIIYWDEQSLFMEHRFV**RP**SD**---K**FVHCIAICRQRVI**D**VSME**A**VMSEL**LP**R**TS

Q9NYI2_44-172 **LLAEQR**FP**GRVLPSDLDLLL**-**HMNNARYLRE**A**DFARV**A**HL**T**RCGVL**GA**LR**E**L**R**A**H**TVLAASCAR**H**RR**S**L**RL**LE**P**FEVRTRLLGWDDRAFYLE**A**RFVS**L**RD**---**GFV**C**ALLR**F**RQHLL**G**TS**P**E**R**VVQHL**CQ**R**RV

Q8E8G6_23-153 F**LGTSRI**TY**RALPSD**C**DI**NF-**H**LT**NSRY**PAF**MD**L**ART**YM**L**A**E**M**GLL**KR**FL**K**L**K**W**M**PI**VN**AAEF**T**Y**I**R**D**I**KP**LK**K**FEIETKVVGWDEK**Y**FYIEQRFIS**E**R**G(4)**V**H**VRGVF**V**CK**G**K**--QI**PLE**V**LVKE**AGY**T**AP

Consensus/80% lhspppl.GbshspDlDhbl.HMNNtRYbREbDFARh.ab.bsslb..bb.b.h.slbtAsphRYRR.l..bp.aplp*+llhWD-ptbabEppFls.pD...sFl+slhbs+Qphh.sshb.lhppb..p..

**YbaW subfamily (10)**

Secondary St. -----E—EE---------HHHHHHHHHHHHHHHH---HHHHHHH---EEEEEE--------------EEEEEE-------EEEEEEEEEEE----EEEE—EEEEEE-------EE—HHHHHHHHHHHH--

Q7NQ84_5-134 **TRIKVRGYHLDLF**G**HVNNARYLEFLEEARWSQFEEQGGLDWFL**Q**SGLALAVVNINIDYRRPA**L**MG**E**QL**V**I**E**T**GM**KSIGNRSAVIHQR**V**LLEGTDTVVAEADVTFVV**F**D**A**K**QN**KAVVLEGQLK**A**MLE**Q**MT**A

Q9JR32_1-127 M**K**LT**VR**N**YHLD**G**Y**G**HVNNARYLEF**F**EEARW**A**FFEERG**L**LH**E**LA**--**GL**I**LIV**AR**I**D**I**R**Y**S**RPAVEGD**V**LQ**F**SCRLKT**P**GTR**RI**VLTQTITL**-**PNGKT**A**AEADIT**LM**PV**HAA**TQR**T**VSL**PA**TL**A**R**A**LE**A**LSE**

Q7N0L9_3-132 **S**I**IKV**H**GYHIDLF**Q**HVNNARYLEFLE**S**ARWEWL**H**KHSV**V**EWI**QR**NNVGFAVVNININYR**SS**AVLGDELEVDCRLTEL**R**NRSGVFSQEII**R**R**R**NNQ**L**IADAK**T**TFV**W**ID**R**KTQRALS**I**EGELREI**MGA**LVK**

P77712_3-132 **TQIKVRGYHLDVY**Q**HVNNARYLEFLEEARWD**G**LENSDSFQWMT**A**HNIAFVVVNININYRRPAVL**S**DLLTITSQLQQLNGKSG**I**LSQ**V**ITLEP**E**GQVVADA**L**ITFVCID**L**KTQKALALEGELREKLE**Q**MVK**

Q8ZC71_3-132 **THIKVRG**F**HIDVF**Q**HVNNARYLEFLEEARWEWLD**G**QPVFQWMV**E**H**Q**IAFIVVNINISYRRPAVLGDLLRIDSQL**L**KLNGKSGVMK**H**TITLEPTA**I**PV**V**DAELTFVCID**Q**RSQKALPLEGELREKLE**E**LVN**

Consensus/80% *pIKVRGYHlDla.HVNNARYLEFLEEARWpbb-ppssbpWbh.psltbhVVNINIsYRRPAVbGDbLplsspLpplss+StVbpQpIhLcssspslA-AclTFVslD.+*Q+AlsLEGpL+cbLE.bsp

**AMP-binding domain associated subfamily (9)**

Secondary St. EEEE-------------------HHHHHHHHHHHHHHHH--------HHHHHH---------EEEEEEE-----EEEEEE-------EEEE----------------

Q8FJ05_26-125 **I**V**LYLD**PM**L**YW**FNGHFA**V**QPLLPGVAQLDWVM**H**YA**T**TLL**AP**G**WR**F**R**SIQNVKFL**AP**L**I**P**E**T**T**VTL**Q**L**T**WQ**E**TS**QV**LTF**C**YQ**RH**D**GDARHT**ASSG**K**I**R**L**-------CR

Q8X6M1_18-117 **I**V**LHL**RA**DL**FW**FRGHFA**V**QPLLPGVAQIDWAM**S**YA**L**NLL**AP**G**WR**F**H**SIQNIKFQ**SP**L**L**P**E**N**R**VTL**A**L**N**WQ**E**ER**QI**LSF**S**YQ**RH**D**GDARHT**ASSG**K**I**R**L**-------CR

Q8Y2A4_482-580 **I**TFT**IP**A**D**HPALP**GHFP**G**HPI**V**PGVVLLD**Q**AI**R**R**IGAA**L**NR**P**FD**A**CR**LSS**A**KFL**SPAA**P**G**V**P**LAL**AFE-ATA**S**GA**IRF**T**VS**AG**E**RE----**VASG**V**L**A**Q**---QEAARP

Q8XSV0_466-563 YE**LRVP**P**EL**VH**FRGHFP**G**LPILPGVVLVDW**I**A**C**LA**A**EL**ADS**T**RS**I**R**SID**Q**LKFM**AP**V**P**P**A**A**L**VS**VQ**L**T**HE**P**QR**CR**VRF**R**AR**LG**T**RE----**CASG**A**L**V**Y**-----READ

Q8E9B2_21-124 **L**R**LLVA**A**DL**EY**FNGHFP**E**Q**A**VLPGVTQLDWAV**R**LG**C**EHF**GY**S**AA**V**A**NLEVLKFQ**QL**I**L**P**GQE**VTL**V**I**S**HN**A**TK**EK**LTF**A**YS**DG**D**HR----**YASG**R**I**S**F**(4)DDGAAQ

Q87V45_425-524 **L**N**LMVP**P**DL**AF**FSGHFP**KA**PVLPGV**L**QV**Q**WAI**N**LG**Q**QLL**DL**P**PD**F**A**GMEVLKFQ**QL**V**R**P**G**D**R**L**K**L**T**L**R**FD**A**TR**AK**LHF**A**FH**NS**E**NA---P**CSSG**RVV**L**----EGAHA

Q7M7K1_21-123 **L**Q**LRVD**H**DL**LD**FSGHFT**H**FPLLPGVTQIDWAM**F**YG**Q**TLL**QC**P**AA**F**K**GMEVIKFQ**EP**I**L**P**D**A**Q**VTL**S**L**V**WD**E**SK**AK**LQF**S**YT**SS**R**DGERIS**HSSG**K**M**K**L**----GASSD

Q8DG00_21-123 **L**Q**LRVD**H**DL**LD**FSGHFT**H**FPLLPGVTQIDWAM**F**YG**Q**TLL**QC**P**AA**F**K**GMEVIKFQ**EP**I**L**P**D**A**Q**VTL**S**L**V**WD**E**SK**AK**LQF**S**YT**SS**R**DGERVS**HSSG**K**M**K**L**----GASSD

Q87RA6_20-122 **L**S**LRVD**A**DI**LD**FQGHFS**H**FPLLPGVTQIDWAL**F**YA**V**QYL**NT**P**KV**F**K**GMEVIKFQ**EP**I**L**P**N**A**D**VCL**S**L**S**WD**N**ER**EK**LTF**K**YT**SK**N**GETLVT**HSSG**K**M**K**L**----GESRE

Consensus/80% l.Lbls.-l..FpGHFs.bPlLPGVsblDWsh.bt.pbb..s..h.sbpslKFb..l.P.s.lsL.l.ap.pp..lpF.hp..p......htSG.b.b.........

**4HBT class I (5)**

Secondary St. --------------------EEE---HHHHHHHHHHHHHHH------HHHHH----EEEEEEEE----EEEE-----EEEEEEHHHH-----EEEEEEEEEE-----EEEEEEE---EEEEE---------EEE-------

P56653_1-136 **M**AR**SI**T**M**Q**QRIEFGDCDPAGIVWFPNYHRWLDAASRNYFIKCGLP**PW**RQT**V**VER**-**GIVGTPIVSCNASF**V**C**T**ASYDDVLTIETCIKEW**R**RKSFVQRHSV**S**RT**TPGGDV-**QLVMRADE**I**RVFA**MN**D**---GE**RLRAI**E**VPADY**

Q9RBQ8_1-137 **M**SR**QV**T**FEQRVEFGDCDPVGIVWFPNYFRWLDAASRHFFISCG**V**P**PW**RTTLAER**-**GIVGTPLVSSNANF**S**SPVSYDDVLLIETEVREW**G**KKSFVQHHRVLRLS**NSGEAP**QLVMQADETRVFAGYQ**---EG**RLRAIPIP**E**DY**

Q8GN83_1-137 **M**PR**QV**T**FEQRVEFGDCDPVGIVWFPNYFRWLDAASRHFFISCGIP**PW**RTTLAER**-**GIVGTPLVSSNATF**S**SPVSYDDVLLIETEVREW**G**RK**R**FMQHHRVLRLS**SCGDAP**QLVMEADETRVFAGYQ**---EG**RLRAIPIPADY**

Q89CF0_1-132 **M**FV**N**R-**RD**VQ**I**QW**GDCDPA**N**IVYYP**R**YF**SM**FDD**S**T**SVL**FEKAGF**S--**KQDLV**H**K**Y**GLVGIP**M**VDT**R**AKF**YI**PSTHGDWITIETKI**ES**I**K**R**S**SF**EV**KHNVYKTA**------**DL**A**IEA**F**ETRV**LV**GRD**PANPE**KLKSAP**F**PAEM**

Q9KBC9_1-127 **M**EG**KV**-**YHFRVKFGD**T**D**A**AGIVFYPNYYKWMD**E**A**C**HHF**LT**E**L**GFP**--T**S**E**L**I**DK**--KI**GFPIV**E**AT**C**QF**K**AP**LL**FADHVFI**R**TSIREL**KD**KSFIL**E**HH**F**IK**Q**G**------**R**V**I**A**S**G**HEKRVWANFS**---NG**KL**AV**CPIPS**SV

Consensus/80% M..pl.bcbRlcFGDCDPsGIVaaPNYa+WbDsA*+paFbpsGbP..+psLs-+.GlVGhPlVsssApF.sPs*asDhlhIETpl+Eb.+KSFhb+HpVb+ht......pLlbpAcEpRVaAsbp.....+L+thPlPt-b
